# Differences Found in the Macroinvertebrate Community Composition in the Presence or Absence of the Invasive Alien Crayfish, *Orconectes hylas*

**DOI:** 10.1371/journal.pone.0150199

**Published:** 2016-03-17

**Authors:** Brandye T. Freeland-Riggert, Stefan H. Cairns, Barry C. Poulton, Christopher M. Riggert

**Affiliations:** 1 Department of Biology and Agriculture, University of Central Missouri, Warrensburg, Missouri, United States of America; 2 U.S. Geological Survey, Columbia Environmental Research Center, Columbia, Missouri, United States of America; 3 Missouri Department of Conservation, Jefferson City, Missouri, United States of America; Towson University, UNITED STATES

## Abstract

Introductions of alien species into aquatic ecosystems have been well documented, including invasions of crayfish species; however, little is known about the effects of these introductions on macroinvertebrate communities. The woodland crayfish (*Orconectes hylas* (Faxon)) has been introduced into the St. Francis River watershed in southeast Missouri and has displaced populations of native crayfish. The effects of *O*. *hylas* on macroinvertebrate community composition were investigated in a fourth-order Ozark stream at two locations, one with the presence of *O*. *hylas* and one without. Significant differences between sites and across four sampling periods and two habitats were found in five categories of benthic macroinvertebrate metrics: species richness, percent/composition, dominance/diversity, functional feeding groups, and biotic indices. In most seasons and habitat combinations, the invaded site had significantly higher relative abundance of riffle beetles (Coleoptera: Elmidae), and significantly lower Missouri biotic index values, total taxa richness, and both richness and relative abundance of midges (Diptera: Chironomidae). Overall study results indicate that some macroinvertebrate community differences due to the *O*. *hylas* invasion were not consistent between seasons and habitats, suggesting that further research on spatial and temporal habitat use and feeding ecology of Ozark crayfish species is needed to improve our understanding of the effects of these invasions on aquatic communities.

## Introduction

Aquatic macroinvertebrates continue to be widely studied because of their unique diversity and ubiquity in streams and rivers worldwide [1–3]. Among aquatic macroinvertebrates, crayfish (Arthropoda: Class Crustacea) are considered “keystone” organisms because their omnivorous feeding strategies and multiple trophic links to other organisms in the benthic community make them an essential piece of freshwater food webs in these ecosystems [4–6]. Benthic macroinvertebrates, including crayfish, provide a vital link in nutrient cycling by accelerating the decomposition of organic matter [7] and providing food to higher trophic levels [8]. Detritus makes up a large part of the diet of several crayfish species [9–11] and crayfish obtain most of their energy for growth from macroinvertebrate food sources [12, 13]. Since crayfish are most often the largest invertebrates in North American freshwater communities, such as the small Ozark stream in this study, they can influence entire food webs by acting as important consumers and prey [14].

The effects of alien crayfish on the native crayfish fauna have been documented [15]; however, relatively few studies have examined the outcomes of an introduced crayfish species on other attributes of the aquatic macroinvertebrate community. When crayfish are moved from their native range and introduced into a new environment, substantial biological and ecological results can occur. Crayfish make excellent invaders because they are agonistic [16], can exploit a variety of aquatic habitats [15], and are omnivorous [17, 18] which can result in effects on multiple trophic levels [7, 16, 19]. The introduction of alien crayfish has been cited as one of the leading causes of declines in crayfish biodiversity [15, 20–22]. Because of the omnivorous nature of crayfish species, invasions of alien crayfish often produce expansive and unpredictable food-web effects [17, 18, 23–25]. There are multiple examples of crayfish invasions causing ecological changes locally, nationally, and globally [26–29]. For example, Rodríguez et al. [30] found the introduction of *Procambarus clarkii* (Girard) into lentic waters of Chozas in León (Northwest Spain) resulted in increased turbidity by decreasing plant coverage by 99%, thus indirectly reducing macroinvertebrate populations by 71%, duck species by 75%, and amphibians by 83%. Houghton et al. [31] showed a 77% decrease in total density of aquatic invertebrates as well as significant differences in trophic guilds correlated with the invading *Orconectes rusticus* (Girard) into Prairie River, Wisconsin.

While crayfish have been moved all across the world by various vectors [32], it should be noted that an alien species need not come from another continent, country, or state. Substantial consequences can occur when fauna are moved from neighboring watersheds. There are at least 36 species of crayfish in Missouri, including 18 species endemic to the Ozark region [33, 34, R DiStefano personal communication], and there have been at least 31 documented cases of crayfish introductions in the state [35, 36]. These introductions have all involved the movement of native Missouri crayfish to regions outside their natural geographic ranges, resulting in the declines of native crayfish populations in the receiving water bodies [35–37]. Among the few studies conducted on Missouri’s crayfish fauna, most have been related to geographic distribution, habitat use, and life histories [33, 37–41]. A few studies have examined feeding preferences [9]; however, no studies within Missouri have characterized effects of crayfish invasions on other community components, making management of these taxa challenging.

A well documented example of a localized crayfish introduction is the movement of the woodland crayfish (*Orconectes hylas* (Faxon)). This species is endemic to the Black River watershed and headwaters of the Meramec and Big Rivers in Missouri and was introduced to the neighboring St. Francis River watershed over 30 years ago [33]. The introduction is thought to have occurred by bait bucket introduction or other intentional releases ([42, 43] R DiStefano personal communication). It has since spread substantially and is implicated in the decline or elimination of native crayfish populations [35, 37], possibly through reproductive advantages [38]. Declines in the relative abundances of native crayfish have been documented with the presence of *O*. *hylas*, and this invasive alien crayfish can reach relative abundances up to 25% greater than native crayfish in the invaded areas [35]. Both the Big Creek crayfish (*Orconectes peruncus* (Creaser)) and St. Francis River crayfish (*Orconectes quadruncus* (Creaser)) are endemic to the upper St. Francis River watershed, upstream of Lake Wappapello [33, 37, 42, 43]. Both endemics have also simultaneously or subsequently experienced population declines and range contractions in areas where *O*. *hylas* has invaded [33, 36, 37]. As a result, both *O*. *peruncus* and *O*. *quadruncus* are listed as imperiled in Missouri (S2) and globally (G2) [44], and as threatened by the American Fisheries Society [22].

The range expansion of *O*. *hylas* within the St. Francis River watershed in the Ozarks provided an opportunity to study the effects of an invasion on a stream system where the upstream movement of this species was ongoing. While it has been demonstrated that *O*. *hylas* is altering the native Missouri crayfish fauna, no research had been conducted to investigate what effects this invasion may have on aquatic macroinvertebrate communities. The objectives of this study were to document the differences in the benthic macroinvertebrate community composition in the presence or absence of the invading *O*. *hylas* in an Ozark stream, and to contribute spatial and temporal data of these effects to guide management and regulatory efforts of nuisance and invasive species in Missouri.

## Methods

### Ethics Statement

The study plan conforms to relevant national and international guidelines regarding ethical treatment, use, and preservation of animals. All field collections of macroinvertebrates were conducted using established sampling protocols, and access to study sites was granted by private landowners. At the time of the sample collections, the primary author and one of the co-authors were employed by the Missouri Department of Conservation, and therefore no permits for collection of macroinvertebrates were necessary.

### Bioinvasion Terminology

There are many terms for organisms that have been moved outside their native ranges. We will follow the naming protocol outlined in Occhipinti-Ambrogi and Galil [45]. *Orconectes hylas* fits the term of “invasive alien” species due to its range expansion and subsequent range contraction of the native crayfish species.

### Watershed Description

**Fig 1:**
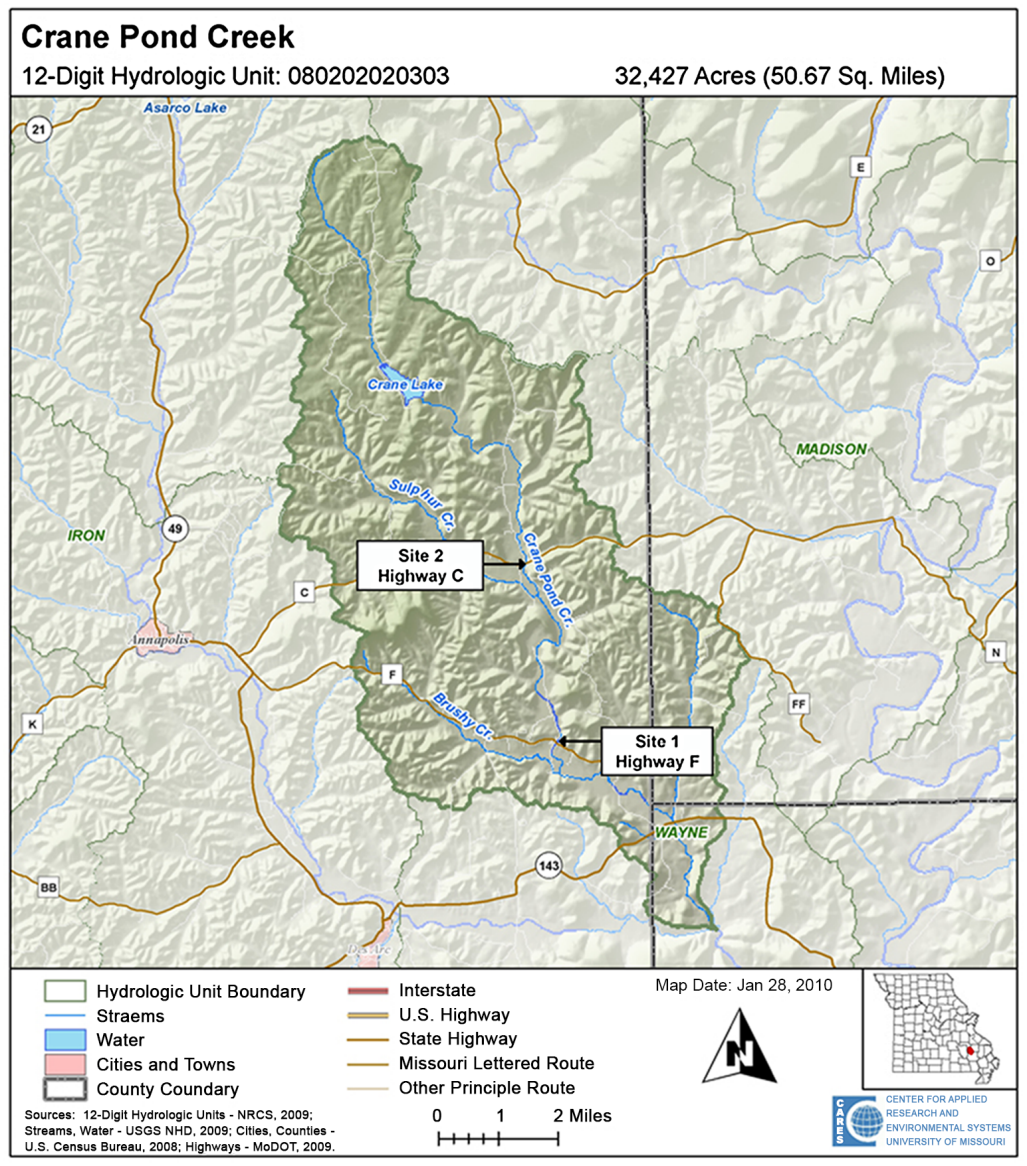
Sampling locations on Crane Pond Creek, Iron County, Missouri, USA in 2011.

Crane Pond Creek is a fourth-order Ozark stream with perennial flow located in Iron County, Missouri [46, 47]. It is 30.8 km in length with a slope of 182 m/km (elevation of 453 m at the headwaters to 130 m at the mouth) [47]. The Crane Pond Creek watershed is a 12-digit hydrologic unit and encompasses an area of 13,118 ha [48] and flows in a southerly direction before it enters Big Creek (Fig 1). The stream is part of the Ozark/Upper St. Francis/Castor Ecological Drainage Unit [49], and is typical of moderate to high-gradient riffle/pool dominated streams located in the Ozark Highlands, containing stream substrates of cherty dolomitic limestone and periodically exposed bedrock layers [50]. The watershed receives an average of 119 cm of annual rainfall [48] and an average minimum-maximum air temperature range of 6.7°C to 32.2°C [50]. The watershed is sparsely populated (2.5 people/km^2^) and is primarily forested (87.7%) with low development (2.27%) and cropland (0%) [48].

### Site Selection

Previous research [51] and reconnaissance efforts determined that Crane Pond Creek was the only stream within the St. Francis River watershed to have both allopatric populations of native *O*. *peruncus* and invading *O*. *hylas*, while also exhibiting similar habitat characteristics throughout its drainage. Since no other streams in the region had the same *O*. *hylas* invasion status, our study was conducted entirely within Crane Pond Creek. Preliminary sampling indicated that *O*. *hylas* had invaded upstream to Highway F in Crane Pond Creek, but had not yet invaded upstream to Highway C (Fig 1). Highway F was chosen as the experimental site (Site 1), and Highway C (Site 2, located 6.8 km upstream of Site 1) was chosen as the control site. UTM coordinates were taken at each location using a Garmin 76SC handheld GPS unit.

### Habitat assessment, water quality and discharge

Discharge and water quality were sampled at both sites during four sampling periods in 2011 spring (9 April), summer (24 June), late summer (29 July), and fall (30 September). Stream discharge was conducted using a Marsh-McBirney Flo-Mate 2000 flow meter at each site during each sampling season. Discharge was calculated as cubic feet per second (cfs) by following the methods listed in the Missouri Department of Natural Resources (MDNR) Flow Measurement in Open Channels Standard Operating Procedure [52]. At each site, field water chemistry parameters were taken during all seasons, and included dissolved oxygen in mg/L (YSI Model 55), temperature in degrees Celsius (YSI Model 30M), specific conductance in μS/cm (YSI Model 30M), and pH (Hach PocketPal).

Habitat quality was evaluated once in September 2011. Stream habitat quality was assessed with the Stream Habitat Assessment Project Procedure (SHAPP) [53]. The SHAPP habitat assessment is a modified version of the EPA Rapid Bioassessment Protocol [54] and has been used by MDNR for evaluating wadeable streams since the mid-1990s ([53], R Sarver personal communication). Within Missouri, this protocol is used as a tool to compare the relative quality of stream habitats, and improve the interpretation of site differences in biological communities between and among streams [R Sarver personal communication]. The protocol utilizes a combination of visual ratings (qualitative) and measurements (quantitative) of physical stream features, and includes 13 individual parameters (range 0‒20 for each), with scores split equally among optimum, suboptimum, marginal, and poor ratings. Parameters assessed included channel morphology, flow and depth, substrate condition (embeddedness and particle size), in-stream cover (within-channel features such as epifaunal substrate or sediment deposition), and riparian/bank integrity (i.e., erosion potential, buffer status, bank stability). Application of the SHAPP habitat assessment protocol results in an overall habitat score for a site that can be used for among-site comparisons; identification of causative factors affecting aquatic macroinvertebrate communities, or to rule out habitat as a controlling factor when water quality or other degradations are suspected [53].

### Benthic Macroinvertebrate Assessment

Aquatic macroinvertebrates were sampled at both sites using the Semi-Quantitative Macroinvertebrate Stream Bioassessment Project Procedure (SMSBPP) [55], which is the standard protocol utilized by the state of Missouri for evaluating the quality of wadeable Missouri streams [53]. The SMSBPP includes separate macroinvertebrate samples from three stream habitats: a) riffles (flowing water over cobble or gravel substrate; b) non-flow (depositional substrate in standing water with no flow, primarily in pools); and c) root mats (with overhanging roots from bank vegetation, and organic debris accumulated in these habitats, [55]. Because root mats were not available in Crane Pond Creek, samples were taken only from coarse substrate (CS) and non-flow (NF) habitats.

For each sampling period and habitat, three random replicates (triplicates) were taken with a D-frame rectangular aquatic kicknet (23 cm x 46 cm, with 500-μm mesh netting). Each sample replicate consisted of a composite of net samples taken at multiple stream locations (three separate 1-m^2^ areas disturbed were composited each for the CS and NF habitats). This resulted in six total samples (three CS and three NF) at each site and for each sampling season (48 total samples). Each composite sample was preserved with 80% buffered ethyl alcohol in 1-L Nalgene bottles, and labeled by habitat type, site, date, and replicate.

### Laboratory Processing

Laboratory processing of macroinvertebrate samples followed the SMSBPP protocol [55], and included subsampling from a gridded tray, random selection of grid numbers, and sorting under 10X magnification until a desired target number was reached (600 for CS habitat and 300 for NF habitat). Larval Chironomidae specimens were mounted on labeled glass slides with CMCP-10 mounting media (Masters Chemical Co., Des Plaines, IL) and allowed to cure for one month before identification with the use of a compound microscope [55]. Macroinvertebrate organisms were identified to the lowest practical taxonomic level, usually genus or species [8, 56, 57]. The level of taxonomic identification, placement of individual taxa into functional feeding groups, and the assignment of tolerance values for calculating the Missouri Biotic Index (BI_tol_) followed the *Taxonomic Levels for Macroinvertebrate Identifications* document developed by the MDNR [58]. Voucher specimens of all macroinvertebrate taxa were retained for verification by experts.

### Indicator Metrics

To provide community-level comparisons between sites, a total of 18 macroinvertebrate indicator metrics were calculated from the data (Table 1). These included core metrics utilized by MDNR for determining aquatic life impairment status of Missouri streams [55], metrics from national Rapid Bioassessment protocols commonly used to assess community-level responses to disturbance or stress [54], and metrics utilized during special studies conducted in the Ozark region [59–61].

**Table 1:** List of macroinvertebrate metrics, references, abbreviations, metric categories and predicted responses to increasing perturbation at biological sampling sites in 2011 at Crane Pond Creek, Iron County, Missouri, USA.

*Indicator Metric and reference*	*Abbreviation*	*Metric Category*	*Response to stress*
***Richness Metrics***			
Total Taxa Richness [54]	TT_rich_	Richness	Decrease
Chironomidae Taxa Richness [98]	Chir_rich_	Richness	Decrease
Ephemeroptera + Plecoptera + Trichoptera Richness [99]	EPT_rich_	Richness	Decrease
***Composition/Percentage Metrics***			
Percent Chironomidae [54]	Chir_cp_	Composition/%	Increase
Percent Elmidae [60, 100]	Elm_cp_	Composition/%	Variable
Percent Ephemeroptera [54]	Eph_cp_	Composition/%	Decrease
Percent Ephemeroptera + Plecoptera [101]	EP_cp_	Composition/%	Decrease
Percent Ephemeroptera + Plecoptera + Trichoptera [54]	EPT_cp_	Composition/%	Decrease
Percent Plecoptera [54]	Plec_cp_	Composition/%	Decrease
Percent Trichoptera [102]	Tric_cp_	Composition/%	Decrease
***Dominance/Diversity Metrics***			
Percent of Dominant taxon [54]	DT1_dd_	Dominance/Diversity	Increase
Percent of 2 Dominant taxa [54]	DT2_dd_	Dominance/Diversity	Increase
Shannon Diversity Index [103]	SDI_dd_	Dominance/Diversity	Decrease
***Functional Feeding Groups Metrics***			
Percent Collectors (Filters + Gatherers) [104]	FiGa_fh_	Functional/Habitat	Variable
Percent Predators [104]	Pred_fh_	Functional/Habitat	Variable
Percent Scrapers [54]	Sc_fh_	Functional/Habitat	Decrease
Percent Shredders [54]	Sh_fh_	Functional/Habitat	Decrease
***Tolerance Metrics***			
Missouri Biotic Index [55]	Bi_tol_	Tolerance	Increase

### Data Analysis

For each site, and within each sample period, means and standard errors were determined from CS and NF habitats. To test for significant differences between sites, a nested, non-parametric analysis of variance (ANOVA) was performed on the means of each macroinvertebrate metric (n = 3 replicates for each site, season, and habitat, α = 0.05) using version 9.3 of the Statistical Analysis System [62] and Proc GLM (General Linear Models). Non-parametric nested ANOVAs were chosen for analysis for the following reasons: 1) among-stream sample replication was not possible because no other streams in the region were known to have the same invasion status (invaded lower reaches and non-invaded upper reaches), 2) non-parametric tests do not rely on normality and equal variance assumptions (neither of which could be tested with only three replicates), and 3) nested sample designs allowed higher degrees of freedom, and as a result, a more robust test with greater statistical power for detecting significant differences between the sites. To provide spatial and temporal comparisons between the sites, two separate nested ANOVAs (*p* < 0.05) were performed for each metric and included a three-way ANOVA with data for the two stream habitats analyzed as separate samples (sampling season x habitat x site, degrees of freedom = 47 including error terms), and a two-way ANOVA with data for the two habitats pooled at each site and sample replicate within a sampling event (sampling season x site, degrees of freedom = 23 including error terms).

## Results

### Water quality, discharge, and habitat assessment

Values for water quality parameters across sampling seasons were all within water quality standards for Missouri [63] with no distinct differences between sites observed. Across seasons, air temperature was 17.3–35.0°C and water temperature ranged from 17.1–24.3°C. Ranges for dissolved oxygen were 7.5–8.8 mg/L, and dissolved oxygen saturation were 82.5–101.0%. Specific conductance ranged from 160–260 μS/cm and pH ranged from 7.4–8.3. Stream discharge ranged from 1.70–17.04 cfs and Site 1 and was 1.03–6.98 cfs at Site 2.

Based on the Missouri habitat assessment protocol [53], both study sites received similar total site scores (Site 1 = 157, Site 2 = 160) and for most individual habitat parameters, only minor differences in flow status, riffle quality, sediment deposition and epifaunal substrate/cover diversity were observed. At both study sites, each of the individual habitat parameters were scored within the optimum (score of 16–20) or sub-optimum (score of 11–15) rating categories.

### Macroinvertebrate assessment

All raw benthic macroinvertebrate data collected in Crane Pond Creek in 2011 are found in S1 Appendix. A total of 151 macroinvertebrate taxa was identified from the two sites [51]. Most taxa (132) were insects; non-insect taxa included mollusks, worms, leeches, and crustaceans. Approximately 41% of the insect taxa were in the three dominant orders of insects typically associated with healthy stream communities and are referred to as EPT taxa (Ephemeroptera, mayflies; Plecoptera, stoneflies; and Trichoptera, caddisflies). Between 21 and 38 EPT taxa occurred at each site, with samples from Site 1 having mean EPT richness of 15–19 and samples from Site 2 having mean EPT richness of 15–21. In addition to EPT taxa, other aquatic insects including midges (Diptera: Chironomidae), dragonflies and damselflies (Odonata), riffle beetles (Coleoptera: Elmidae), water pennies (Coleoptera: Psephenidae), aquatic heteropterans (Hemiptera) and hellgrammites (Megaloptera: Corydalidae) were commonly encountered in the samples. Among the crayfish specimens found in the macroinvertebrate samples across all sampling periods and habitats, there were 143 crayfish collected at Site 1 (*O*. *hylas* = 95% with *O*. *peruncus* absent), and 85 crayfish collected at Site 2 (*O*. *hylas* absent, *O*. *peruncus* = 85%). It should be noted, the sampling protocol employed does not specifically target crayfish, but rather benthic macroinvertebrates in general and therefore only presence/absence can be determined using the crayfish numbers above.

Mean taxonomic richness in data pooled by habitat (CS+NF) was similar at both sites across all seasons and ranged between 46‒63 taxa at Site 1 and 57‒70 taxa at Site 2. Individual taxa (*Stenelmis lateralis*:Coleoptera) by habitat (NF) represented a mean relative abundance of up to 52.4% of macroinvertebrates at Site 1; however, most taxa were present in low abundances and represented less than 2% of the macroinvertebrates across all habitats and sampling periods. During all four sampling seasons, riffle beetles in the genus *Stenelmis* (Elmidae) were the most dominant taxon at Site 1 in both habitats, and this was the only taxon that was among the five most dominant organisms in both habitats and at both sites. Other taxa such as the mayflies *Caenis* sp. and *Stenonema femoratum* (Say) were also dominant in the NF habitat at Site 2 during the late summer and fall sampling seasons. The midges *Tanytarsus* sp., *Cladotanytarsus* sp. and *Polypedilum aviceps* (Townes), as well as the caddisfly *Cheumatopsyche* sp. (Hydropsychidae) were also among the most dominant taxa at Site 2. Overall, there were 13 macroinvertebrate taxa present at Site 2 that were not collected at Site 1. These include two crayfish (*O*. *peruncus* and *Cambarus hubbsi* (Creaser)) and insects belonging to the orders Diptera (3 taxa, including 2 midges), Ephemeroptera (1 taxon), Plecoptera (3 taxa), Trichoptera (2 taxa), Odonata (2 taxa), and Hemiptera (1 taxon). In contrast, there were 19 taxa present at Site 1 that were not collected at Site 2. These included mollusks (3 taxa), crayfish (*O*. *hylas*), and insects belonging to the orders Diptera (3 midge taxa), Ephemeroptera (3 taxa), Plecoptera (2 taxa), Trichoptera (3 taxa), Odonata (3 taxa), and Coleoptera (2 taxa). However, none of these listed taxa found at only one of the study sites were among the five dominant taxa in any habitat or season, and in most cases made up less than 5% of sample relative abundances. Individual values and ranges across seasons for richness, relative abundance, and dominance of macroinvertebrate taxa, as well as summary statistics for the individual metric values determined from the samples, are given in Freeland-Riggert [51].

### Taxa Richness Metrics

**Fig 2:**
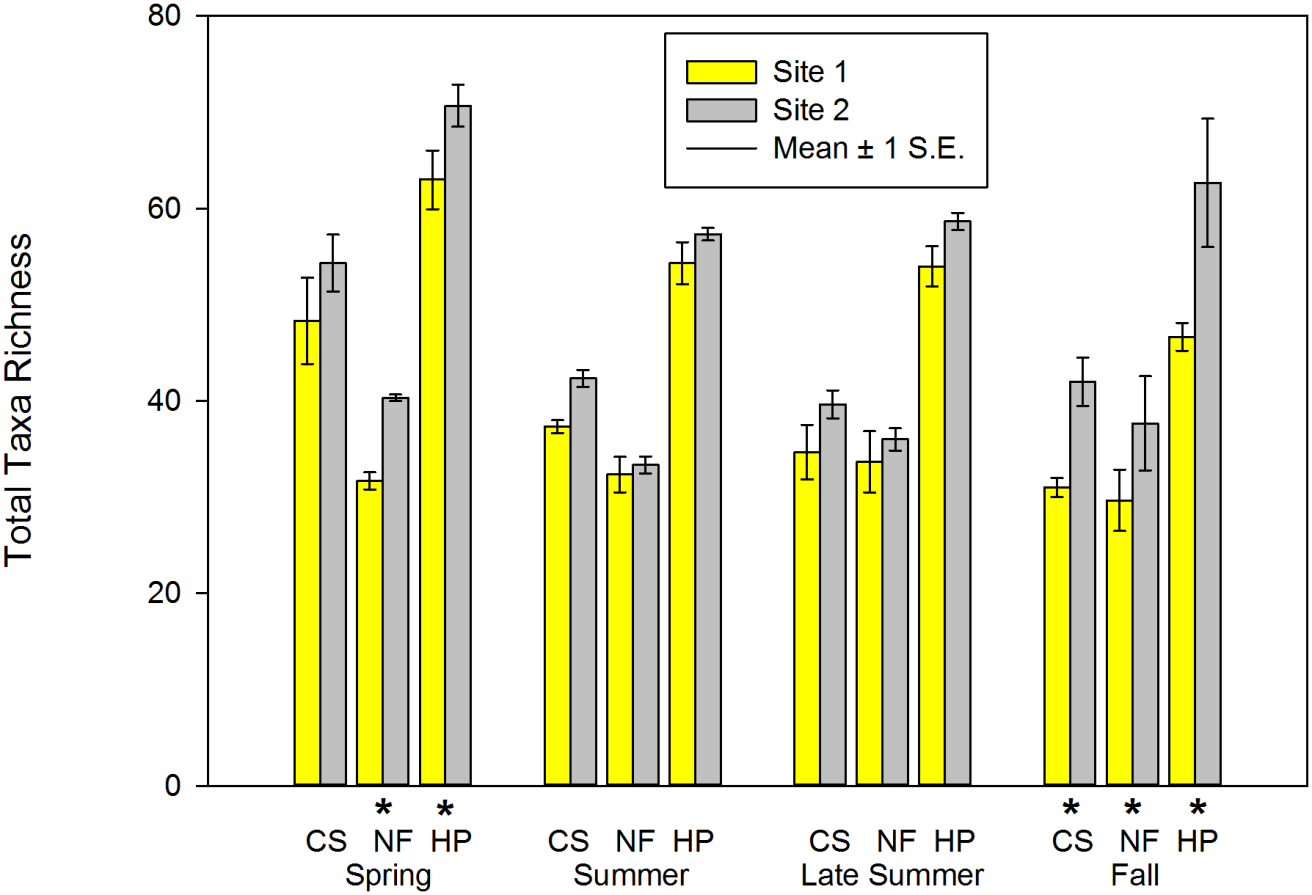
Total Taxa Richness of macroinvertebrate communities sampled during four periods in two habitats at Crane Pond Creek, Iron County, Missouri, USA in 2011. Bars represent mean and range of n = 3 samples, with ± 1 S.E. Habitats: CS = coarse substrate, NF = non-flow, HP = both habitats pooled. * = significant differences detected between sites.

**Fig 3:**
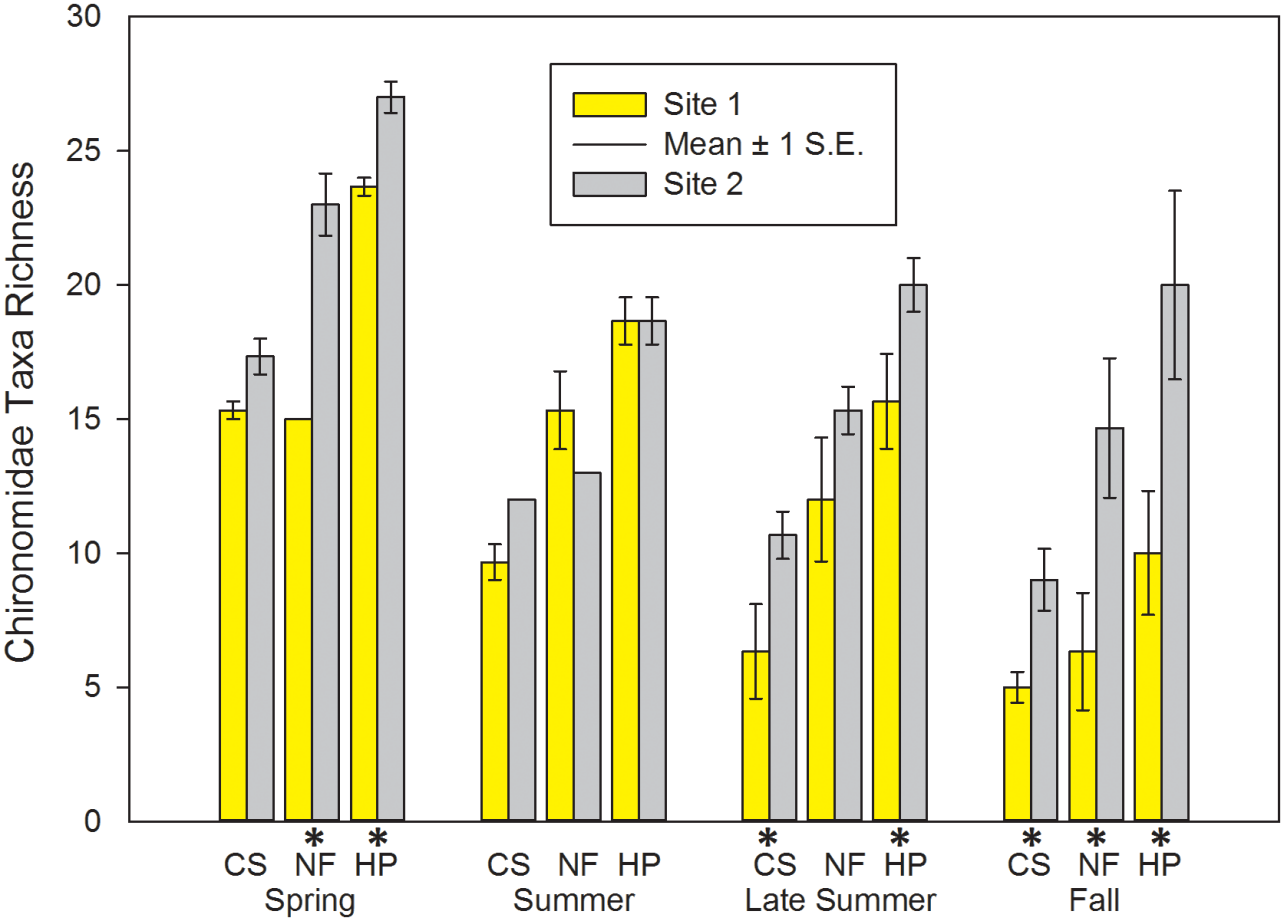
Chironomidae Taxa Richness of macroinvertebrate communities sampled during four periods in two habitats at Crane Pond Creek, Iron County, Missouri, USA in 2011. Bars represent mean and range of n = 3 samples, with ± 1 S.E. Habitats: CS = coarse substrate, NF = non-flow, HP = both habitats pooled. * = significant differences detected between sites.

In general, total richness (TT_rich_), mean EPT (EPT_rich_), and Chironomidae taxa richness (Chir_rich_) were higher at Site 2 during one or more seasons and habitats (Tables 2–5; Figs 2 and 3). Similarly, mean Chir_rich_ was significantly higher in NF at Site 2 during spring and pooled samples, during late summer in both CS and pooled samples, and during fall in all three habitat types examined (Tables 2–5; Fig 3). EPT_rich_ were significantly higher at Site 2 in summer (CS and Pooled) and fall samples (CS only); however, mean EPT_rich_ in NF was significantly higher in fall at Site 1 (Tables 2–5).

**Table 2:** Statistical significance (Pr> │+│from ANOVA, α = 0.05) in macroinvertebrate metrics between Site 1 (invaded) and Site 2 (control) in two habitats sampled from Crane Pond Creek, Iron County, Missouri, USA in Spring 2011. Metric abbreviations are defined in Table 1. NS = Not Significant.

	Coarse (CS)	Non-Flow (NF)	Pooled (CS+NF)
	Diff/p-value	Diff/p-value	Diff/p-value
*Richness Metrics*			
TT_rich_	NS	**1<2 (0.0190)**	**1<2 (0.0058)**
Chir_rich_	NS	**1<2 (<0.0001)**	**1<2 (0.0006)**
EPT_rich_	NS	NS	NS
*Composition Metrics*			
Chir_cp_	**1<2 (<0.0001)**	NS	**1<2 (<0.0001)**
Elm_cp_	**1>2 (<0.0001)**	**1>2 (0.0086)**	**1>2 (<0.0001)**
Eph_cp_	NS	NS	NS
EP_cp_	NS	NS	NS
EPT_cp_	NS	NS	NS
Plec_cp_	NS	NS	NS
Tric_cp_	NS	NS	NS
*Dominance/Diversity Metrics*			
DT1_dd_	**1>2 (0.0040)**	NS	**1>2 (0.0109)**
DT2_dd_	**1>2 (0.0069)**	NS	**1>2 (0.0041)**
SDI_dd_	NS	NS	NS
*Functional Feeding Groups Metrics*			
FiGa_fh_	NS	NS	NS
Pred_fh_	NS	NS	NS
Sc_fh_	**1>2 (0.0007**)	NS	**1>2 (0.0023)**
Sh_fh_	**1<2 (<0.0001**)	NS	**1<2 (<0.0001)**
*Tolerance Metrics*			
Bi_tol_	**1<2 (0.0009**)	NS	**1<2 (0.0013)**

**Table 3:** Statistical significance (Pr> │+│from ANOVA, α = 0.05) in macroinvertebrate metrics between Site 1 (invaded) and Site 2 (control) in two habitats sampled from Crane Pond Creek, Iron County, Missouri, USA in Summer 2011. Metric abbreviations are defined in Table 1. NS = Not Significant.

	Coarse (CS)	Non-Flow (NF)	Pooled (CS+NF)
	Diff/p-value	Diff/p-value	Diff/p-value
*Richness Metrics*			
TT_rich_	NS	NS	NS
Chir_rich_	NS	NS	NS
EPT_rich_	**1<2 (0.0022)**	NS	**1<2 (0.0114)**
*Composition Metrics*			
Chir_cp_	**1<2 (0.0308)**	NS	**1<2 (0.0106)**
Elm_cp_	**1>2 (0.0007)**	NS	**1>2 (0.0003)**
Eph_cp_	NS	NS	NS
EP_cp_	NS	NS	NS
EPT_cp_	NS	NS	NS
Plec_cp_	NS	NS	NS
Tric_cp_	NS	NS	NS
*Dominance/Diversity Metrics*			
DT1_dd_	NS	NS	NS
DT2_dd_	**1>2 (0.0377)**	NS	NS
SDI_dd_	NS	NS	NS
*Functional Feeding Groups Metrics*			
FiGa_fh_	NS	NS	NS
Pred_fh_	**1<2 (0.0406)**	NS	NS
Sc_fh_	**1>2 (0.0043)**	NS	NS
Sh_fh_	NS	NS	NS
*Tolerance Metrics*			
Bi_tol_	NS	NS	NS

**Table 4:** Statistical significance (Pr> │+│from ANOVA, α = 0.05) in macroinvertebrate metrics between Site 1 (invaded) and Site 2 (control) in two habitats sampled from Crane Pond Creek, Iron County, Missouri, USA in Late Summer 2011. Metric abbreviations are defined in Table 1. NS = Not Significant.

	Coarse (CS)	Non-Flow (NF)	Pooled (CS+NF)
	Diff/p-value	Diff/p-value	Diff/p-value
*Richness Metrics*			
TT_rich_	NS	NS	NS
Chir_rich_	**1<2 (0.0263)**	NS	**1<2 (0.0064)**
EPT_rich_	NS	NS	NS
*Composition Metrics*			
Chir_cp_	NS	NS	NS
Elm_cp_	NS	**1>2 (0.0025)**	**1>2 (0.0201)**
Eph_cp_	NS	**1<2 (<0.0001)**	**1<2 (<0.0001)**
EP_cp_	NS	**1<2 (<0.0001)**	**1<2 (<0.0001)**
EPT_cp_	NS	**1<2 (0.0002)**	**1<2 (0.0031)**
Plec_cp_	NS	NS	NS
Tric_cp_	NS	NS	NS
*Dominance/Diversity Metrics*			
DT1_dd_	NS	NS	NS
DT2_dd_	NS	NS	NS
SDI_dd_	NS	NS	NS
*Functional Feeding Groups Metrics*			
FiGa_fh_	NS	NS	NS
Pred_fh_	NS	NS	NS
Sc_fh_	NS	NS	NS
Sh_fh_	NS	NS	NS
*Tolerance Metrics*			
Bi_tol_	NS	**1<2 (<0.0001)**	**1<2 (0.0047)**

**Table 5:** Statistical significance (Pr> │+│from ANOVA, α = 0.05) in macroinvertebrate metrics between Site 1 (invaded) and Site 2 (control) in two habitats sampled from Crane Pond Creek, Iron County, Missouri, USA in Fall 2011. Metric abbreviations are defined in Table 1. NS = Not Significant.

	Coarse (CS)	Non-Flow (NF)	Pooled (CS+NF)
	Diff/p-value	Diff/p-value	Diff/p-value
*Richness Metrics*			
TT_rich_	**1<2 (0.0037)**	**1<2 (0.0294)**	**1<2 (0.0006)**
Chir_rich_	**1<2 (0.0391)**	**1<2 (<0.0001)**	**1<2 (<0.0001)**
EPT_rich_	**1<2 (0.0136)**	**1>2 (0.0136)**	NS
*Composition Metrics*			
Chir_cp_	NS	NS	NS
Elm_cp_	NS	**1>2 (0.0001)**	**1>2 (<0.0001)**
Eph_cp_	NS	**1<2 (<0.0001)**	**1<2 (<0.0001)**
EP_cp_	NS	**1<2 (<0.0001)**	**1<2 (0.0002)**
EPT_cp_	NS	**1<2 (0.0033)**	**1<2 (0.0021)**
Plec_cp_	NS	**1>2 (0.0011)**	**1>2 (0.0004)**
Tric_cp_	NS	NS	NS
*Dominance/Diversity Metrics*			
DT1_dd_	NS	**1>2 (0.0078)**	**1>2 (0.0074)**
DT2_dd_	NS	**1>2 (0.0387)**	**1>2 (0.0094)**
SDI_dd_	NS	**1<2 (0.0009)**	**1<2 (0.0013)**
*Functional Feeding Groups Metrics*			
FiGa_fh_	NS	**1<2 (0.0024)**	**1<2 (0.0023)**
Pred_fh_	NS	NS	NS
Sc_fh_	NS	**1>2 (0.0004)**	**1>2 (0.0002)**
Sh_fh_	NS	NS	NS
*Tolerance Metrics*			
Bi_tol_	**1<2 (0.0047)**	**1<2 (<0.0001)**	**1<2 (0.0001)**

### Composition/Percentage Metrics

**Fig 4:**
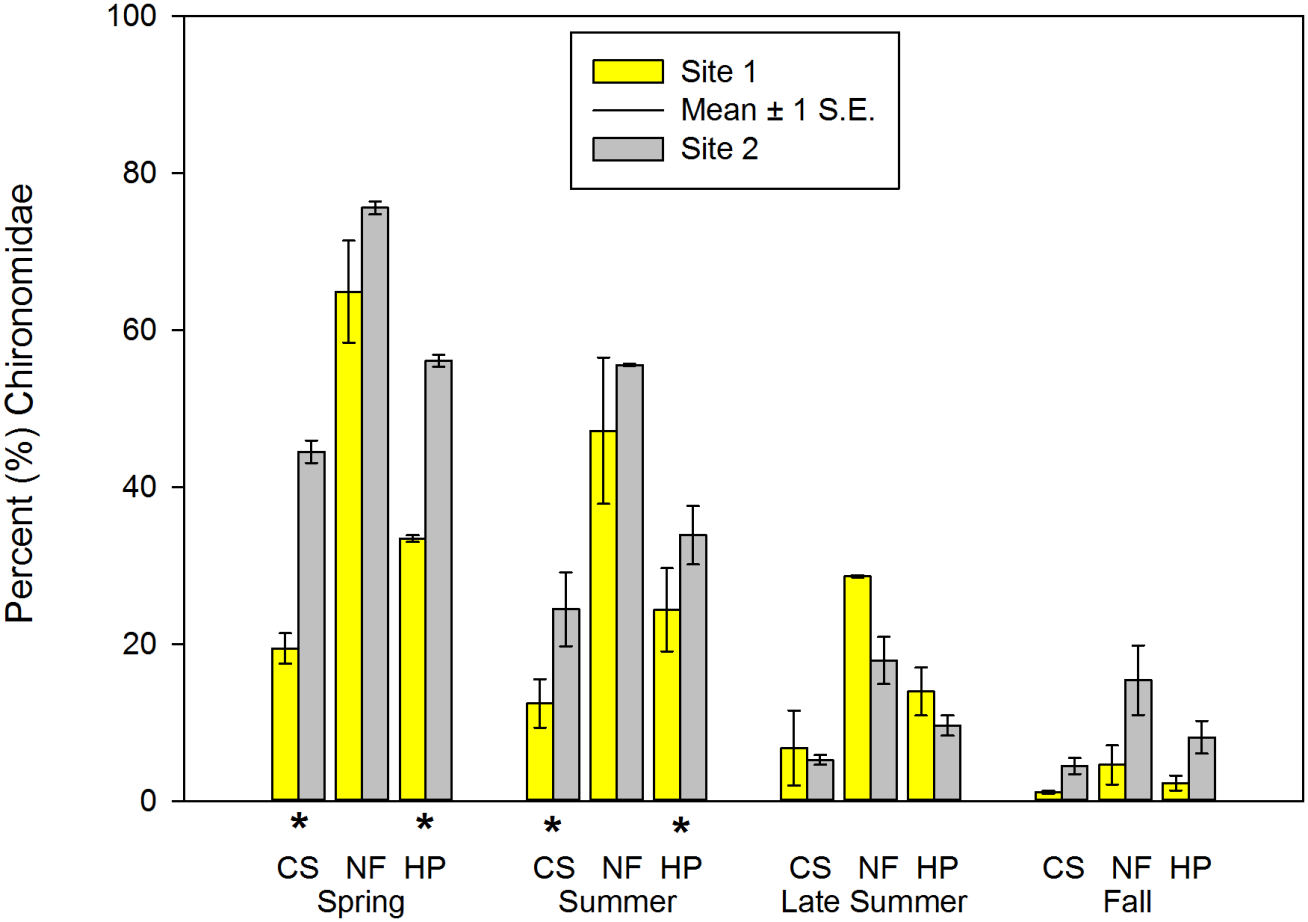
Percent (%) Chironomidae of macroinvertebrate communities sampled during four periods in two habitats at Crane Pond Creek, Iron County, Missouri, USA in 2011. Bars represent mean and range of n = 3 samples, with ± 1 S.E. Habitats: CS = coarse substrate, NF = non-flow, HP = both habitats pooled. * = significant differences detected between sites.

**Fig 5:**
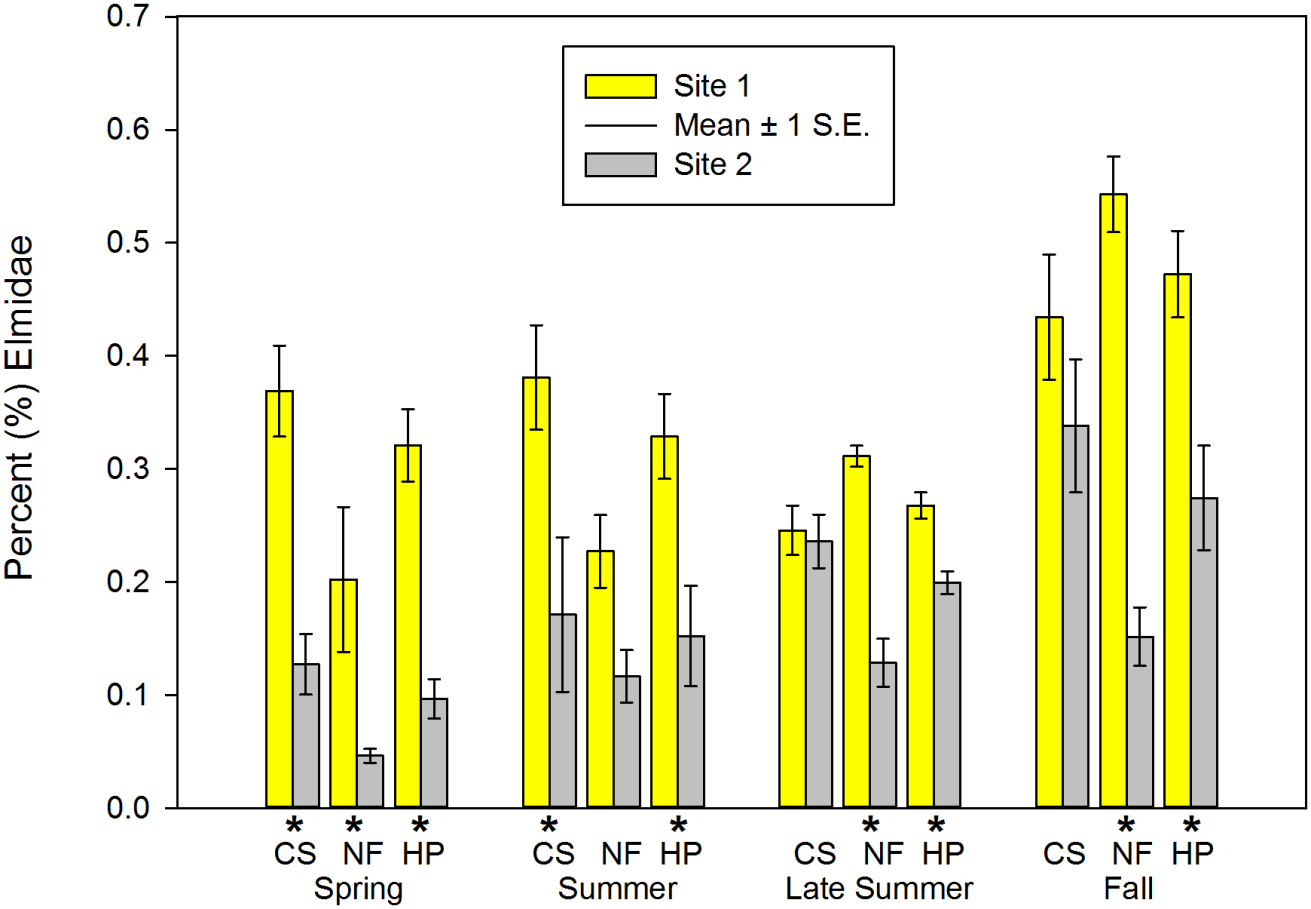
Percent (%) Elmidae of macroinvertebrate communities sampled during four periods in two habitats at Crane Pond Creek, Iron County, Missouri, USA in 2011. Bars represent mean and range of n = 3 samples, with ± 1 S.E. Habitats: CS = coarse substrate, NF = non-flow, HP = both habitats pooled. * = significant differences detected between sites.

**Fig 6:**
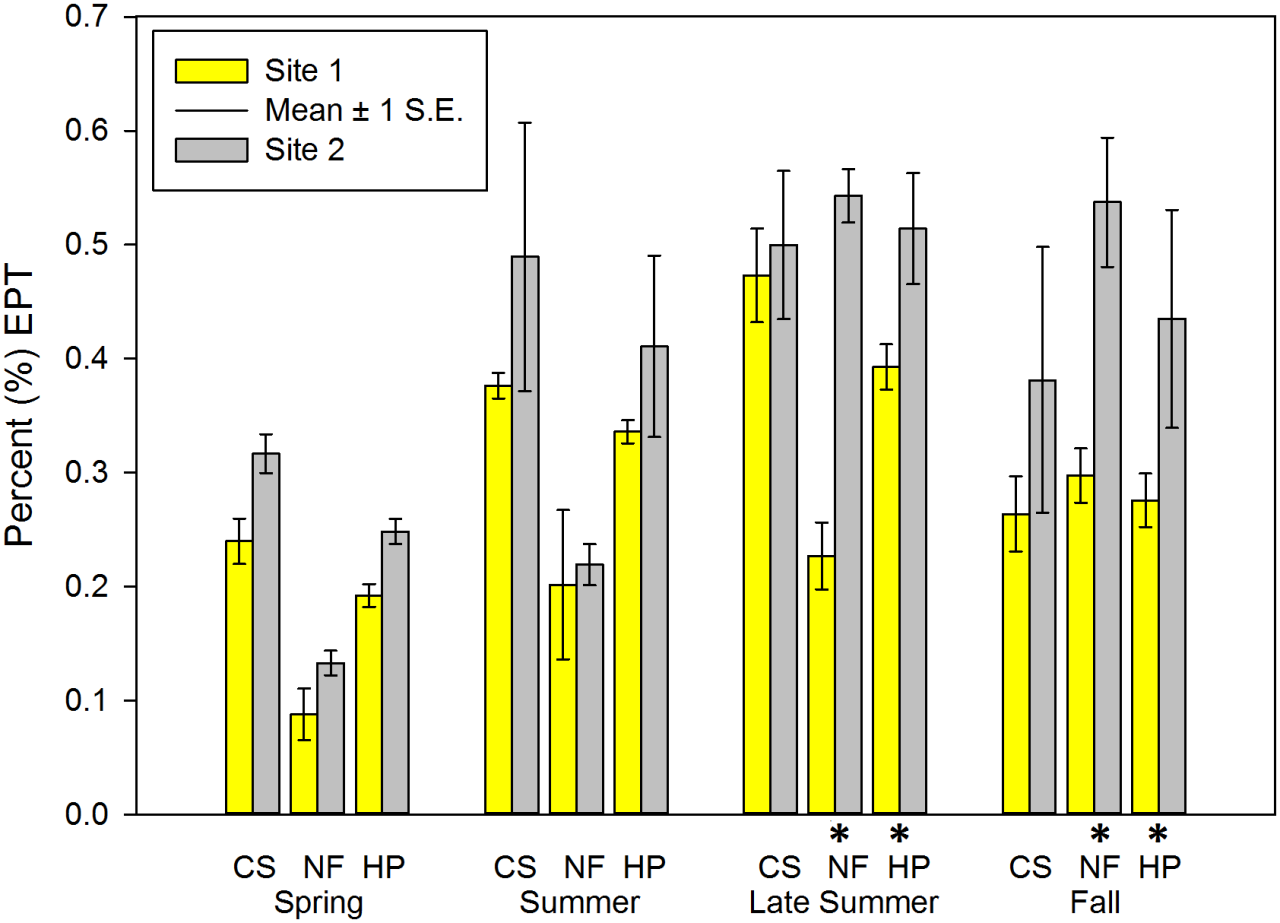
Percent (%) EPT (Ephemeroptera; Plecoptera; Trichoptera) of macroinvertebrate communities sampled during four periods in two habitats at Crane Pond Creek, Iron County, Missouri, USA in 2011. Bars represent mean and range of n = 3 samples, with ± 1 S.E. Habitats: CS = coarse substrate, NF = non-flow, HP = both habitats pooled. * = significant differences detected between sites.

Mean Chir_cp_ in CS and pooled samples was significantly higher at Site 2 in spring and summer (Tables 2 and 3 and Fig 4). Mean Elm_cp_ (spring = all three habitat types, summer = CS and pooled) was significantly higher at Site 1 (Tables 2 and 3; Fig 5). In three of the EPT-related metrics in this category (Eph_cp_, EP_p_, EPT_cp_), Site 2 had significantly higher mean values than Site 1 in both NF and pooled habitat types during late summer and fall samples (Tables 4 and 5, Fig 6). During fall, mean Ple_cp_ in NF and pooled habitat types were significantly higher at Site 1. Mean Elm_cp_ in late summer and fall was significantly higher at Site 1 at NF and pooled habitat types (Tables 4 and 5, Fig 5). No significant differences were found between sites in any habitat or season for mean Tric_cp_ (Tables 2–5).

### Dominance/Diversity Metrics

Mean values for the dominant taxa metrics DT1_dd_ and DT2_dd_, were significantly higher at Site 1 during spring (both CS and pooled habitat types) and fall (both NF and pooled habitat types; Tables 2–5). DT2_dd_ was also significantly higher at Site 1 in CS during summer (Table 3) Mean DT1_dd_ and DT2_dd_ were both significantly higher in both NF and pooled habitat types at Site 1, and mean SDI_dd_ was significantly lower at Site 1 in the fall (Table 5).

### Functional Feeding Group Metrics

Mean FiGa_fh_ were not significantly different during spring, summer, or late summer (Tables 2–4); however, mean FiGa_fh_ was significantly higher in NF and pooled habitats at Site 2 during fall (Table 5). Pred_fh_ showed no significant difference between sites during spring, late summer or fall (Tables 2–5), but mean values were significantly higher in CS at Site 1 during summer (Table 3). Mean Sc_fh_ was significantly higher in both CS and pooled habitat Site 1 during spring and summer (Tables 2 and 3); however, there were no significant differences in late summer between sites (Table 4). In fall, mean Sc_fh_ were significantly higher in NF and pooled habitats at Site 1 (Table 5). Mean Sh_fh_ showed no significant differences between sites for summer, late summer or fall samples (Tables 3–5); however mean Sh_fh_ was significantly higher in CS and pooled habitats at Site 2 during the spring (Table 2).

### Tolerance Metrics

**Fig 7:**
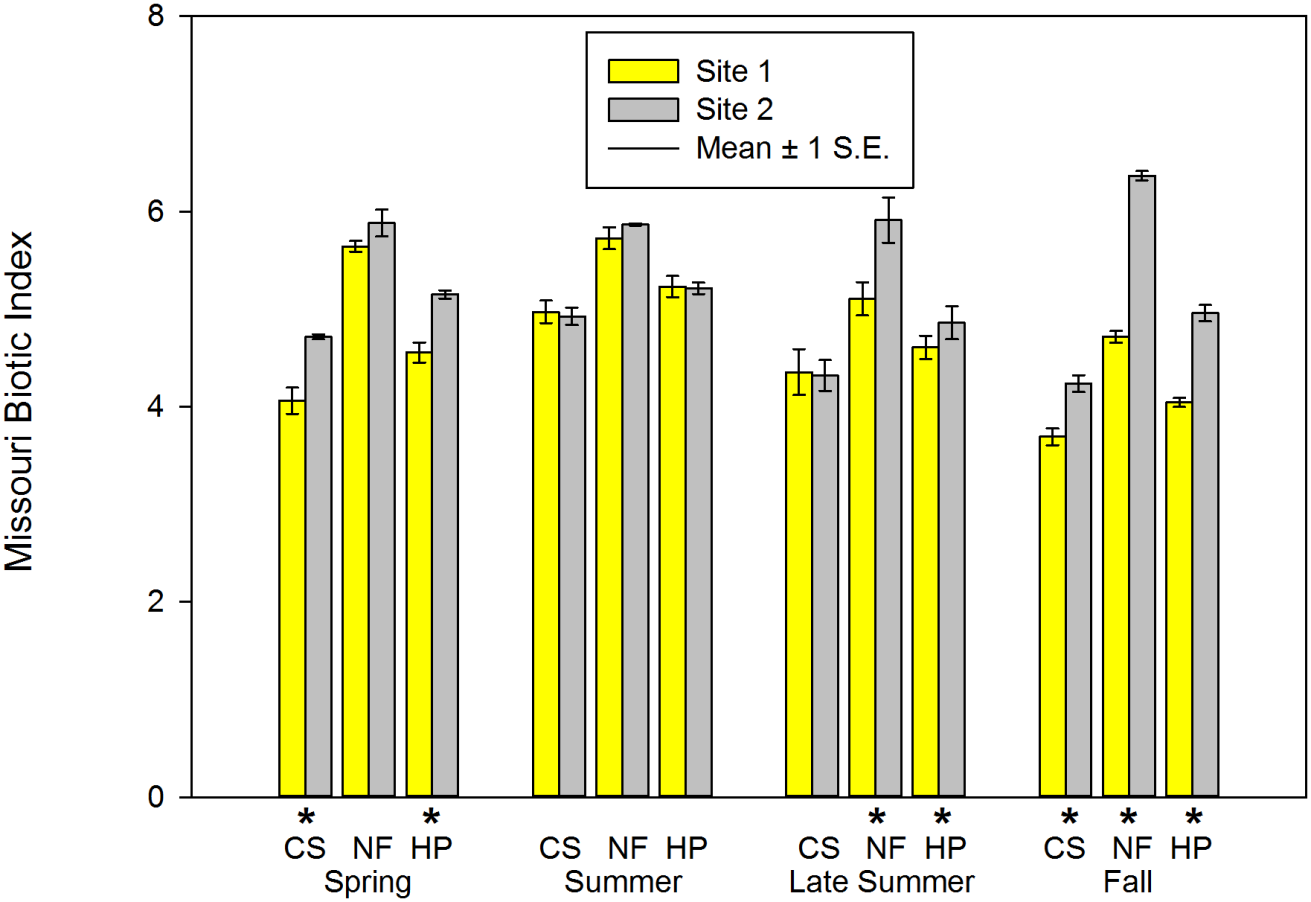
Missouri Biotic Index of macroinvertebrate communities sampled during four periods in two habitats at Crane Pond Creek, Iron County, Missouri, USA in 2011. Bars represent mean and range of n = 3 samples, with ± 1 S.E. Habitats: CS = coarse substrate, NF = non-flow, HP = both habitats pooled. * = significant differences detected between sites.

Mean BI_tol_ were significantly higher in CS and pooled habitat at Site 2 during spring (Table 2; Fig 7); in NF and pooled habitats during late summer (Table 4; Fig 7), and during fall in all three habitat types (Table 5; Fig 7). No significant differences were observed in mean BI_tol_ between sites during the summer season (Table 3; Fig 7).

### Interactions among Season, Habitat, and Site

The nested non-parametric ANOVA, which determined statistically significant differences between the two sampling sites, also generated significant interaction terms for both the two-way and three-way analyses (Table 6). Interaction terms from the overall two-way analysis (P x S) were significant for six different metrics. Interaction terms from the three-way analysis were significant for 16 metrics (P x S = 8 metrics; P x H = 12 metrics; H x S = 5 metrics; P x H x S = 7 metrics). The only two metrics significantly different between sites in at least one season or habitat that did not generate a significant interaction among the three variables were Pred_fh_ and Sc_fh_ (Tables 2, 3, 5 and 6). Conversely, all possible interaction combinations among variables were significant for Sh_fh_ and BI_tol_ (Table 6). Except for two metrics (Sh_fh_ and BI_tol_), all of the significant interactions observed from the two-way analysis (P x S, Table 6) were found in cases where Site 1 had significantly higher values than Site 2 (Tables 2–5). In most of these cases, the significant interactions were observed in the spring and fall periods. Based on the two-way analysis, significant interactions were also observed between period and site for two metrics where Site 2 was significantly higher than Site 1. These included Sh_fh_ (spring season only, Tables 2 and 6), and BI_tol_ (all periods except summer, Tables 3 and 6).

**Table 6:** Statistical significance (Pr > F values) for interaction terms from analysis of site differences in metric values (non-parametric nested ANOVA, α = 0.05) for Crane Pond Creek, Iron County, Missouri, USA. Macroinvertebrates sampled in two habitats during four time periods in 2011. Metric abbreviations are given in Table 1. NS = not significant, P = Sampling Period (season), H = Habitat, S = Site.

	^a^ 2-Way ANOVA	^b^ 3-Way ANOVA			
Metric	P*S	P*S	P*H	H*S	P*H*S
*Richness Metrics*					
TT_rich_	NS	NS	**0.0026**	NS	NS
Chir_rich_	NS	**0.0138**	NS	NS	**0.0272**
EPT_rich_	NS	NS	**0.0047**	**0.0114**	**0.0192**
*Composition Metrics*					
Chir_cp_	**0.0013**	**0.0009**	**<0.0001**	NS	NS
Elm_cp_	NS	NS	NS	NS	**0.0020**
Eph_cp_	NS	**0.0051**	**0.0002**	**0.0001**	**0.0174**
EP_cp_	NS	**0.0499**	**0.0003**	**0.0012**	**0.0117**
EPT_cp_	NS	NS	**0.0012**	NS	NS
Plec_cp_	**0.0185**	**0.0057**	NS	NS	NS
Tric_cp_	NS	NS	**0.0242**	NS	NS
*Dominance/Diversity Metrics*					
DT1_dd_	**0.0100**	NS	**0.0344**	NS	NS
DT2_dd_	**0.0046**	NS	NS	NS	NS
SDI_dd_	NS	**0.0174**	**0.0023**	NS	NS
*Functional Feeding Groups Metrics*					
FiGa_fh_	NS	NS	**0.0203**	NS	NS
Pred_fh_	NS	NS	NS	NS	NS
Sc_fh_	NS	NS	NS	NS	NS
Sh_fh_	**0.0074**	**0.0005**	**<0.0001**	**0.0123**	**0.0019**
*Tolerance Metrics*					
BI_tol_	**0.0015**	**<0.0001**	**0.0022**	**0.0019**	**0.0008**

^a^ Period by Site (habitats pooled)

^b^ Period by Habitat by Site

Significant two-way interactions were observed in the three-way nested ANOVA for P x S (eight metrics), P x H (12 metrics), and H x S (five metrics). Among these, Tric_cp_ was the only metric that was not significantly different between sites in any habitat or season (Tables 2–6). Only one significant interaction existed for each of the TT_rich_, Chir_rich_, EPT_cp_, Plec_cp_, Tric_cp_, DT1_dd_, and FiGa_fh_ metrics (Table 6) and among these, most were interactions between sampling period and habitat. Most of the significant interactions involved CS habitat in spring and summer, and NF habitat in late summer and fall (Tables 2–5). Results of the three-way analysis determined significant three-way interactions (P x H x S) in seven metrics (Table 6). Among these, there were four metrics where all possible interactions among factors were significant, including Eph_cp_, EP_cp_, Sh_fh_, and BI_tol_ (Tables 2–5). Except for the Sh_fh_, metric in spring season, all of these corresponded with one or more habitats or seasons where Site 1 was significantly higher than Site 2 (Tables 2–6).

## Discussion

We examined the macroinvertebrate community in Crane Pond Creek, Iron County, Missouri during the upstream expansion of an alien crayfish (*O*. *hylas*) which was in the process of displacing the native *O*. *peruncus* while the study was being conducted. Identification and enumeration of crayfish specimens found in macroinvertebrate samples confirmed that no *O*. *hylas* were present at the upstream site (Site 2) during the entire duration of the study. The ecology of Ozark crayfish species and their feeding and habitat preferences are relatively poorly known, but some aspects can provide further interpretation of our study results. Early research on crayfish has suggested they gain most of their nutritional needs from detritus and plant material, but this belief has been overemphasized [64]. Food choice [65] and growth experiments [66–68] conducted with a range of crayfish species fed on natural foods clearly show a preference for animal food over that from detrital sources. Many crayfish species exhibit an ontogenetic shift in diet, whereby post-hatch and juvenile crayfish feed predominantly on aquatic invertebrates and adults feed mainly on detritus [28, 69, 70]. This shift has been explained both in terms of increased protein needs for growth by juvenile crayfish [64] and inability of larger crayfish to catch fast moving invertebrate prey [71]. Gut analyses showed there is a tendency for small rapidly-growing crayfish to positively select midges (Chironomidae) before they reach this ontongenic shift in their diet as they mature [4, 9, 72, 73].

Spatial and temporal habitat use for *O*. *hylas* and *O*. *peruncus* has shown both species utilize riffles, runs, and pools [37, 38]. However, *O*. *hylas* has a presumed competitive advantage with higher fecundity, earlier egg hatching, larger size, and ability to obtain greater densities [38]. DiStefano et al. [38] further found young *O*. *hylas* at their highest densities in riffle habitat during the spring. Currently, there is no information regarding specific food habits of these Ozark crayfish, however, previous research has demonstrated that macroinvertebrates are a primary source of food for stream crayfish [12, 13, 23]. Taken together, it is likely the increased abundance of young of year crayfish, potentially having higher feeding rates, may alter some macroinvertebrate community characteristics. Therefore, our study assumes that certain macroinvertebrates could be heavily utilized as a prey item during the time that an invasive alien crayfish species is in the process of expanding its range.

### Water quality and habitat conditions

The overall water quality and habitat results from Crane Pond Creek indicate that minor differences in stream characteristics appear unlikely as major factors affecting the differences in macroinvertebrate communities between the sites. Our study sites had very similar overall channel conditions, bank and riparian conditions, and availability of instream habitat. Habitat differences were either represented by a one or two point difference in scores of the individual habitat parameters, or were based on supplemental visual observations (e.g. vegetative canopy cover, algae and periphyton growth, relative size of cobble substrate in riffles). Among the parameters that showed a greater difference between sites, flow status scored lower at the downstream site based on a greater relative percentage of exposed gravel in the stream channel. However, this parameter may be highly variable because large exposed gravel bars are a common feature of Ozark streams [74], and therefore this parameter may not be a good indicator of flow status in the region. The lower score for riffle quality at the upstream site was only due to the differences in relative length and width of riffles, and not due to the slight differences in the size of cobble substrate that we observed. Similarly, water quality and flow characteristics between the sites were minor, except for the higher discharge at the invaded site during spring. Water temperature and associated levels of dissolved oxygen are known to influence the distribution of macroinvertebrate taxa because of their effects on organism metabolism, growth, development, reproduction, and food availability [1, 75–78]. However, no notable site differences were observed for any of these parameters measured in Crane Pond Creek and all values fell within normal ranges according to Missouri State Water Quality Standards [63].

As previously mentioned, it has been documented *O*. *peruncus* [37] and *O*. *hylas* [38] utilize both pool (equivalent to our NF habitat) and riffle (equivalent to our CS habitat) habitats in the upper St. Francis drainage. In our study, most crayfish found in the macroinvertebrate samples were from the CS habitat, with only 5.9% (Site 1) and 16.4% (Site 2) collected in the NF habitat. However, the sampling protocol and relatively small mesh size of the D-frame kick net (500 μm) we used for sampling does not provide efficient estimates of crayfish abundance due to their high mobility and larger size as compared to other macroinvertebrates. Although smaller-scale habitat preferences are not known for *O*. *hylas*, the minor differences in habitat between our sites did not provide any evidence that habitat was a factor affecting our observed differences in crayfish abundance. This follows previous Missouri studies in watersheds where introduced crayfish have invaded or native species have been displaced. For example, studies conducted with both *O*. *hylas* (introduced into the St. Francis watershed) and one subspecies of the ringed crayfish (*Orconectes neglectus chaenodactyleus* (Williams) introduced into the Spring River watershed) have shown that overall stream habitat is not limiting [37, 38, 41, 79]. Even though the habitat evaluation we utilized was not comprehensive, it is possible that the minor localized site differences we observed in canopy cover and periphyton growth may have had some influence on benthic macroinvertebrate communities, including crayfish.

### Macroinvertebrate Composition/Percentage

Several macroinvertebrate indicators showed significant differences between sites where *O*. *hylas* was present compared to where it was absent. In addition to the 13 macroinvertebrate taxa that were present at Site 2 and absent at the invaded site, at least one of the taxa richness metrics was significantly higher at the non-invaded site during each season and habitat type. Only EPT richness during fall in the NF habitat showed the opposite pattern where richness was significantly higher at the invaded site. Significant site differences were more frequently observed in abundance/composition metrics than in the richness metrics in Crane Pond Creek. During one or more seasons and in one or more habitat types, significantly lower relative abundances of several taxa were observed at the invaded site, including midges (Chir_cp_) and three metrics related to EPT organisms (Eph_cp_, EP_cp_, EPT_cp_). Similarly, an increase in relative abundance of riffle beetles in the family Elmidae (Elm_cp_) was also observed at the invaded site. This overall result is similar to McCarthy et al. [25], who used across-Order comparisons to demonstrate that non-native *O*. *rusticus* crayfish had the greatest negative effects on abundance and composition of benthic macroinvertebrates.

Our data also suggest that the invasion of *O*. *hylas* may have caused a decline in community evenness. In at least one season and habitat, the invaded site had significantly higher values for one or both of the dominant taxa metrics (DT1_dd_, DT2_dd_) and significantly lower Shannon Diversity Index values (SDI_dd_). These results might be expected considering the high abundances of *Stenelmis lateralis* (Family Elmidae), which was the most dominant taxon at the invaded site during all seasons and in each habitat type [51].

### EPT Abundance

The results of this study demonstrate the value of including multiple indicator metrics associated with each of the EPT orders of macroinvertebrates when studying the effects of introduced crayfish species. It is known that macroinvertebrates belonging to these insect orders are commonly ingested by crayfish [4, 9, 17, 23–25, 29], but it is not known whether invasive alien crayfish may increase their feeding rate on these organisms as they have invaded stream reaches. Abundances of both EPT (EPT_cp_) and mayflies (Eph_cp_) were significantly lower in NF and pooled habitats in late summer and fall. Previous literature suggests that effects of invasive alien crayfish on mayfly abundance may be attributed to their behavior. For example, Nyström [5] indicated that the behavioral traits of different mayfly families may affect their vulnerability as crayfish prey items. Poor swimmers (e.g. Heptageniidae) cling tightly to rock surfaces, while strong swimmers (e.g. Isonychiidae) and species that burrow into silt substrates (e.g. Caenidae) may be less likely to be captured or consumed as food items by crayfish [80, 81]. Conversely, slow moving crawlers (e.g. Leptohyphidae: *Tricorythodes* sp.) may be more vulnerable to predation [8, 80]. This may partially explain why Heptageniidae mayflies were the most abundant of the EPT organisms in all habitats across all seasons at the invaded site (Site 1). In contrast, Plecoptera abundance (Ple_cp_) was significantly higher in NF habitat at the invaded site during fall. Some species in this order are known to migrate to certain habitats during various parts of their life cycle [82], which may result in seasonal changes in habitat use. The higher relative abundance of Plecoptera at Site 1 is counter to results from other studies; Crawford et al. [24] found a decrease in stonefly abundance in the presence of the introduced crayfish *Pacifastacus leniusculus*. Trichoptera abundance (Trich_cp_) was lower in CS and pooled habitats during summer, late summer and fall. This is similar to McCarthy et al. [25] who showed *O*. *rusticus* reduced numbers of Trichoptera. However, Whiteledge and Rabeni [9] found that Trichoptera larvae were rare in gut contents of Ozark crayfish species, and the differences in caddisfly abundance we detected in our study were not statistically significant.

### Chironomidae Richness and Abundance

As previously mentioned, midges (Chironomidae) are a key food item for crayfish. In our study, both richness (Chir_rich_) and relative percent abundance (Chir_cp_) of this group of insects were significantly lower at the invaded site, with abundance lower in CS and pooled habitats during spring and early summer, and richness lower in one or more habitat types in spring, late summer, and fall. Lower abundance and richness of Chironomidae has been previously documented following the introductions of two other invasive alien crayfish into U.S. waters: the signal crayfish (*Pacifastacus leniusculus* (Dana)) and the red swamp crayfish (*Procambarus clarkii* (Girard)) [24, 28, 72, 83]. Midges are soft bodied and ubiquitous in nearly every stream habitat, and have multivoltive life cycles [8], making them readily available as crayfish prey items throughout the year [5, 9, 84–86]. Comparisons of *O*. *hylas* and the native and closely related *O*. *peruncus* may provide some insight to reduced abundances of midges at the downstream site during the spring and early summer. As previously discussed, *O*. *hylas* has been shown to have reproductive advantages over *O*. *peruncus*, as well as *O*. *hylas* young exhibiting higher densities in riffle habitat during the spring [38]. This suggests that *O*. *hylas* may feed on these soft-bodied invertebrates at a higher rate as compared to the native crayfish species present in the stream, and that their competitive advantage may result in reduced abundances of Chironomidae during some seasons, as we observed in our study.

### Elmidae Abundance

Significant declines in this family of beetles have been associated with the presence of introduced *P*. *leniusculus* in Finland [86]. Even though that study was conducted in a lentic system, we observed a significantly higher abundance of Elmidae in the presence of *O*. *hylas* within both habitats during all four sampling seasons. The riffle beetle *Stenelmis* spp. was the most dominant member of the family Elmidae, and overall, among the most dominant taxa at the invaded site for every season and habitat except NF in the spring. Few studies have examined Elmidae as a prey item for crayfish, however this family is known to exploit multiple habitats in streams because both larvae and adults are aquatic while occupying more than one trophic feeding strategy (larvae = scrapers, adults = gathering collectors) [8]. A majority of Elmidae collected in our study were larvae, which have a thick, leathery integument [8], possibly making them less palatable as a food source for *O*. *hylas*. Studies conducted on North American adult Elmidae have confirmed that crayfish, turtles and some fish find them unpalatable as a food item [87]. Adult and larval riffle beetles also appear to be rarely preyed upon by predatory aquatic insects [88, 89], and previous research has not shown Coleoptera in crayfish diet analysis [9, 24]. In addition to the aforementioned Chironomidae, scrapers such as snails (Mollusca: Gastropoda) are a common food resource for crayfish because they are slow-moving and provide calcium for rebuilding exoskeleton components [26, 29, 90, 91]. Therefore, it is possible that decreases of other taxa preyed upon by crayfish may allow scrapers such as larval Elmidae to exploit algae food resources, resulting in their increased abundance.

### Study Interactions

The Crane Pond Creek data were analyzed as a nested study design because all factors were intentionally left as independent variables, and comparable habitats were sampled at both sites independently during the same time period to isolate effects that may result from the crayfish invasion. We did not expect nor anticipate interactions between these factors; therefore, we presented and interpreted our study results for each season and habitat independently. In contrast, a factorial study design would have been more appropriate if our factors were intentionally treated as co-dependent on one another, because interactions are an expected and planned result [92] and interaction terms are interpreted differently between these two designs. Further, not all significant interactions or site differences observed in our study may be ecologically significant. Statistically significant interactions associated with individual metrics may indicate that the crayfish invasion (main treatment effect) was not the only factor affecting differences in the macroinvertebrate community between sites, because interacting factors such as habitat and sampling season may create interference and false-positives in the study results. Our analysis also did not measure the relative importance of different factors affecting macroinvertebrates, nor did we measure spatial or temporal crayfish movement or feeding ecology among habitat types. Therefore, we have assumed that the most ecologically meaningful and easily interpreted effects of the main treatment (crayfish invasion) on macroinvertebrate indicator metrics, would occur in cases where significant differences between sites are observed without a corresponding interaction that is statistically significant. We have also assumed that significant effects of the *O*. *hylas* invasion (i.e. declines in richness or changes in abundance) would most likely be the result of macroinvertebrate taxa or functional group being utilized as a food source, rather than effects from competition between invasive alien crayfish and other macroinvertebrates.

A significant interaction between period and habitat is expected if a certain habitat is being utilized more or less by invasive alien crayfish across different seasons, or if invasive alien crayfish are utilizing a different food source across different habitats. For both nested ANOVA results (2-way and 3-way), this interaction was not significant in three metrics with significant site differences, including Chir_rich_ (1<2), Elm_cp_ (1>2), and Sc_fh_ (1>2). Significant interactions between habitat and site are expected if a habitat type has different availability to crayfish at one site or another, or if foraging habitat or diel feeding preferences of invasive alien crayfish are different than the native crayfish at one site or another. For both nested ANOVA results (2-way and 3-way), this interaction was not significant in five metrics with significant site differences, including Chir_rich_ (1<2), Elm_cp_ (1>2), EPT_cp_ (1<2), SDI_dd_ (1<2), and Sc_fh_ (1>2). Significant interactions between period and site are expected if invasive alien crayfish are utilizing both sites differently depending on the season, or if they are feeding uniformly at each site but differently across seasons. For both nested ANOVA results (2-way and 3-way), this interaction was not significant in five metrics with significant site differences, including TT_rich_ (1<2), EPT_rich_ (1<2), Elm_cp_ (1>2), EPT_cp_,(1<2) and Sc_fh_ (1>2). We also might expect significant three-way interactions (Period x Habitat x Site) if invasive alien crayfish are affecting the macroinvertebrate community at the two study sites differently, depending on all combinations of season (4 periods of collection), and habitat type (CS, NF, pooled). For both nested ANOVA results (2-way and 3-way), this three-way interaction was not significant for only three metrics, including EPT_cp_ (1<2), SDI_dd_ (1<2), and Sc_fh_ (1>2). From the 3-way ANOVA results (CS and NF habitats not pooled), other metrics with significant site differences included Chir_cp_ (1<2, CS = spring and early summer, pooled = spring), Plec_cp_ (1>2, NF = fall), and the two dominant taxa metrics as indicators of community evenness DT1_dd_ (1>2, CS = spring, NF = fall) and DT2 _dd_ (1>2, CS = spring, summer, NF = fall). Significant site differences in these indicator metrics were not consistent across habitat types, seasons or interactions, therefore these differences could be caused by the presence of either invasive alien crayfish, other unknown factors that were not measured during the study (i.e. diel crayfish movement or changes in habitat use), or both. However, most site differences we observed in the indicator metrics were consistent across seasons and habitats (1>2 or 1<2), indicating that these macroinvertebrate community attributes are the ones most likely being affected by the presence of *O*. *hylas* crayfish at the invaded site.

Since the habitat assessment we conducted during this study did not include measurements of seasonal habitat availability or spatial/temporal changes in habitat use by crayfish or other macroinvertebrates, the causes of these interactions cannot be determined with any certainty. Literature suggests that some crayfish species forage in different habitats during different seasons and/or times of the day [93–95]. However, if a specific foraging habitat is utilized by *O*. *hylas* at the invaded site but not utilized by native crayfish (i.e. *O*. *peruncus*) at the upstream, non-invaded site, this might explain why significant interactions were not present for some indicator metrics. These interactions can more easily be explained in relation to the crayfish invasion, and appear to indicate that both sampling season and habitat are affecting the observed differences in macroinvertebrate metrics between sites. Considering our overall results and interpretation of interactions, the presence of *O*. *hylas* at the invaded site (Site 1) resulted in decreased richness of total, EPT and Chironomidae, decreases in Chironomidae abundances, changes in functional groups (scrapers) and overall community evenness, increases in Elmidae, and declines in overall diversity. However, our data also suggests that other factors are influencing macroinvertebrate community composition at the study sites at specific habitats or during some seasons, and the relative degree to which these other factors are affecting the macroinvertebrate communities at both sites cannot be determined.

### Implications of Results

Results from our study highlight differences in macroinvertebrate community composition between an invaded and non-invaded site in Crane Pond Creek, where *O*. *hylas* crayfish have replaced the native *O*. *peruncus*. The invaded site had significantly different total, midge (Chironomidae) and EPT taxa richness, abundances of riffle beetles (Elmidae) and EPT organisms, and community evenness compared to the non-invaded site during more than one sampling season and in more than one habitat. We also observed significant site differences even in cases where interactions between variables were not significant. Because we observed changes in the same macroinvertebrates utilized as prey items by crayfish, it is possible that the invasion has the potential to cause ecological harm to resident macroinvertebrate communities. Overall, literature suggests that the competitive advantages of alien *O*. *hylas* over the native *O*. *peruncus* (earlier hatching, higher fecundity and population size noted earlier) may reduce macroinvertebrate abundances and richness or cause shifts in community dominance.

The effects we observed have potential implications for streams in the Ozark region of Missouri. In particular, newly hatched *O*. *hylas* obtain their highest densities in riffle habitat during spring and early summer [38], the same habitat and time periods where we observed statistically significant declines in abundances and richness of crayfish food items such as EPT taxa and midges. Previous studies have shown that crayfish may select protein-rich animal matter as food sources to support fast growth and energy maintenance [9, 12, 73]. Not all of the observed site differences could be fully interpreted since no seasonal habitat preference or feeding ecology studies exist for *O*. *hylas*, and very few of these studies have been conducted on Ozark crayfish species in general [9]. Our results suggest that increased numbers of invasive alien crayfish appear likely as the cause of reduced densities and taxa richness in midges and other macroinvertebrates that are being utilized as a food source, especially during spring and early summer seasons when densities are potentially the highest. Because we observed significant interactions among factors (site, habitat and season), our results also suggest that other poorly studied aspects of crayfish ecology such as diel or seasonal shifts in habitat use or foraging strategies may be confounding factors affecting the results. It is also possible that both reductions in Chironomidae and increases in Elmidae in the presence of *O*. *hylas* may cause changes in the trophic interactions and ecological functions that are beyond the scope of this study.

We also observed significant site differences in macroinvertebrate indicator metrics that are commonly used to evaluate the quality of aquatic life in Ozark streams. We utilized the same macroinvertebrate protocol and habitats included in these assessments, and our spring and fall data coincides with their recommended index periods for sampling [52]. Total (TT_rich_) and EPT (EPT_rich_) richness, Shannon Diversity Index (SDI_dd_), and the Missouri Biotic Index (Bitol) are the four core metrics used to evaluate wadeable stream quality. In particular, we did not expect a significant difference between our study sites in the BI_tol_, which is a pollution-based indicator that is sensitive to nutrient enrichment [55, 96]. Values for this metric were significantly lower (i.e., lower nutrient enrichment) at the invaded site in one or more habitats during each sampling season. We did not measure nutrient levels during this study, but no obvious signs of nutrient enrichment were observed at either site (e.g. lack of excessive algal growth throughout the sampling seasons, or observable differences in algal coverage between sites). All four of the core metrics utilized for stream quality assessments by MDNR were significantly different between sites during either spring or fall seasons (or both) in at least one habitat. Even though we could not compare our metric values with reference stream criteria in Crane Pond Creek (due to the lack of rootmat habitat), the presence of invasive alien crayfish species at a stream site may be an important consideration when interpreting future biological assessment results. If an invasive alien crayfish alters the macroinvertebrate community composition, it may shift values for these metrics lower or higher than expected, and in turn alter the accuracy in classification of stream impairment for meeting aquatic life expectations as part of Clean Water Act goals [55, 63, 97]. The presence of invasive alien crayfish species is not currently treated as a factor in pollution assessments conducted with macroinvertebrates in Missouri streams. Our results for these four biotic assessment metrics suggest that the presence of invasive alien species should be considered when evaluating stream quality in regions throughout their invasion and after established populations exist. Frequent follow-up sampling at reference sites that have been invaded would also provide additional interpretations when sites are being compared. Additionally, further research characterizing feeding ecology and spatial/temporal habitat use by invasive crayfish is needed to provide further understanding of these effects on stream macroinvertebrate communities.

## Supporting Information

S1 Appendix Raw benthic macroinvertebrate data from Crane Pond Creek, Iron County, Missouri, USA. (PDF)
