# Supplementary material for: Differences Found in the Macroinvertebrate Community Composition in the Presence or Absence of the Invasive Alien Crayfish, Orconectes hylas
Source: PLoS One. 2016 Mar 17;11(3):e0150199. doi: 10.1371/journal.pone.0150199 (PMC4795676; doi:10.1371/journal.pone.0150199)
Supplement: S1 Appendix — (PDF) [file pone.0150199.s001.pdf]

S1 Appendix. Raw benthic macroinvertebrate data from Crane Pond Creek, Iron County, Missouri, USA.

Date	Site	Habitat	Sample	Taxa	Family	Order	Qty	Notes
4/9/2011	1	CS	1	Isoperla sp.	Perlodidae	Plecoptera	2	L/R
4/9/2011	1	CS	1	Plecoptera		Plecoptera	1	
4/9/2011	1	CS	1	Eurylophella sp.	Ephemerellidae	Ephemeroptera	1	L/R
4/9/2011	1	CS	1	Rheotanytarsus sp.	Chironominae	Diptera	6	
4/9/2011	1	CS	1	Fallceon sp.	Baetidae	Ephemeroptera	2	
4/9/2011	1	CS	1	Gomphidae	Gomphidae	Odonata	1	
4/9/2011	1	CS	1	Helichus sp.	Dryopidae	Coleoptera	3	
4/9/2011	1	CS	1	Helicopsyche sp.	Helicopsychidae	Tricoptera	1	L/R
4/9/2011	1	CS	1	Rheocricotopus sp.	Orthocladiinae	Diptera	1	
4/9/2011	1	CS	1	Hemerodromia sp.	Empididae	Diptera	2	
4/9/2011	1	CS	1	Hydropsyche sp.	Hydropsychidae	Tricoptera	6	
4/9/2011	1	CS	1	Isonychia bicolor	Isonychiidae	Ephemeroptera	2	
4/9/2011	1	CS	1	Acroneuria frisoni	Perlidae	Plecoptera	8	L/R
4/9/2011	1	CS	1	Eurylophella sp.	Ephemerellidae	Ephemeroptera	2	
4/9/2011	1	CS	1	Isoperla signata	Perlodidae	Plecoptera	4	
4/9/2011	1	CS	1	Ephemeroptera		Ephemeroptera	2	
4/9/2011	1	CS	1	Maccaffertium mediopunctatum	Heptageniidae	Ephemeroptera	19	
4/9/2011	1	CS	1	Maccaffertium mediopunctatum	Heptageniidae	Ephemeroptera	5	L/R
4/9/2011	1	CS	1	Tanytarsus sp.	Chironominae	Diptera	1	
4/9/2011	1	CS	1	Maccaffertium terminatum	Heptageniidae	Ephemeroptera	7	
4/9/2011	1	CS	1	Neoperla falayah	Perlidae	Plecoptera	1	
4/9/2011	1	CS	1	Neoperla sp.	Perlidae	Plecoptera	8	
4/9/2011	1	CS	1	Oecetis sp.	Leptoceridae	Tricoptera	12	
4/9/2011	1	CS	1	Optioservus sp.	Elmidae	Coleoptera	28	
4/9/2011	1	CS	1	Optioservus sandersoni	Elmidae	Coleoptera	1	
4/9/2011	1	CS	1	Orconectes hylas	Cambaridae	Decapoda	6	L/R
4/9/2011	1	CS	1	Orconectes luteus	Cambaridae	Decapoda	3	L/R
4/9/2011	1	CS	1	Lumbriculidae	Lumbriculidae	Lumbriculida	1	L/R
4/9/2011	1	CS	1	Perlidae	Perlidae	Plecoptera	3	
4/9/2011	1	CS	1	Acroneuria frisoni	Perlidae	Plecoptera	2	
4/9/2011	1	CS	1	Chironomidae Pupae	Chironomidae	Diptera	9	
4/9/2011	1	CS	1	Argia sp.	Coengrionidae	Odonata	3	L/R
4/9/2011	1	CS	1	Baetidae	Baetidae	Ephemeroptera	1	
4/9/2011	1	CS	1	Parachironomus sp.	Chironominae	Diptera	1	
4/9/2011	1	CS	1	Argia sp.	Coengrionidae	Odonata	7	
4/9/2011	1	CS	1	Bezzia sp.	Ceratopogonidae	Diptera	2	

S1 Appendix. Raw benthic macroinvertebrate data from Crane Pond Creek, Iron County, Missouri, USA.

4/9/2011	1	CS	1	Orthocladius sp. (yellow dome tooth)	Orthocladiinae	Diptera	11	
4/9/2011	1	CS	1	Chimarra sp.	Philopotamidae	Tricoptera	1	L/R
4/9/2011	1	CS	1	Nanocladius sp.	Orthocladiinae	Diptera	5	
4/9/2011	1	CS	1	Ancyronyx sp.	Elmidae	Coleoptera	1	Larvae
4/9/2011	1	CS	1	Microtendipes sp.	Chironominae	Diptera	1	
4/9/2011	1	CS	1	Amphinemura sp.	Nemouridae	Plecoptera	1	
4/9/2011	1	CS	1	Chironomidae	Chironomidae	Diptera	99	
4/9/2011	1	CS	1	Polypedilum fallax	Chironominae	Diptera	3	
4/9/2011	1	CS	1	Polypedilum (sp. A)	Chironominae	Diptera	26	
4/9/2011	1	CS	1	Helicopsyche sp.	Helicopsychidae	Tricoptera	40	
4/9/2011	1	CS	1	Corydalis sp.	Corydalidae	Megaloptera	5	L/R
4/9/2011	1	CS	1	Cricotopus isocladius	Orthocladiinae	Diptera	3	
4/9/2011	1	CS	1	Corydalis sp.	Corydalidae	Megaloptera	2	
4/9/2011	1	CS	1	Cheumatopsyche sp.	Hydropsychidae	Tricoptera	12	
4/9/2011	1	CS	1	Agnetina flavescens	Perlidae	Plecoptera	4	
4/9/2011	1	CS	1	Polypedilum convictum	Chironominae	Diptera	20	
4/9/2011	1	CS	1	Elimia potosensis	Pleuroceridae	Gastropoda	3	L/R
4/9/2011	1	CS	1	Elimia potosensis	Pleuroceridae	Gastropoda	12	
4/9/2011	1	CS	1	Elimia potosensis	Pleuroceridae	Gastropoda	3	
4/9/2011	1	CS	1	Ablabesmyia sp.	Tanypodinae	Diptera	2	
4/9/2011	1	CS	1	Elmidae sp.	Elmidae	Coleoptera	1	Larvae
4/9/2011	1	CS	1	Tubificidae	Tubificidae	Tubificida	1	
4/9/2011	1	CS	1	Ephemerellidae	Ephemerellidae	Ephemeroptera	3	
4/9/2011	1	CS	1	Eukiefferiella sp.	Orthocladiinae	Diptera	7	
4/9/2011	1	CS	1	Tipula sp.	Tipulidae	Diptera	1	
4/9/2011	1	CS	1	Psephenus herricki	Psephenidae	Coleoptera	40	
4/9/2011	1	CS	1	Stenonema femoratum	Heptageniidae	Ephemeroptera	1	
4/9/2011	1	CS	1	Stylogomphus albistylus	Gomphidae	Odonata	1	L/R
4/9/2011	1	CS	1	Tabanus sp.	Tabanidae	Diptera	2	
4/9/2011	1	CS	1	Stenelmis sp.	Elmidae	Coleoptera	144	
4/9/2011	1	CS	1	Stenelmis lateralis	Elmidae	Coleoptera	21	
4/9/2011	1	CS	1	Stenelmis sp.	Elmidae	Coleoptera	1	L/R
4/9/2011	1	CS	1	Simulium sp.	Simuliidae	Diptera	4	
4/9/2011	1	CS	1	Thienemannimyia grp	Tanypodinae	Diptera	8	
4/9/2011	1	CS	1	Thienemanniella sp.	Orthocladiinae	Diptera	3	
4/9/2011	1	CS	1	Tricoptera		Tricoptera	1	
4/9/2011	1	CS	1	Siphlonurus sp.	Siphlonuridae	Ephemeroptera	2	

S1 Appendix. Raw benthic macroinvertebrate data from Crane Pond Creek, Iron County, Missouri, USA.

4/9/2011	1	CS	1	Viviparus sp.	Viviparidae	Mesogastropoda	1	L/R
4/9/2011	1	CS	1	Viviparus sp.	Viviparidae	Mesogastropoda	3	
4/9/2011	1	CS	1	Psephenus herricki	Psephenidae	Coleoptera	1	L/R
4/9/2011	1	CS	2	Rheocricotopus sp.	Orthocladiinae	Diptera	1	
4/9/2011	1	CS	2	Agapetus sp.	Glossosomatidae	Tricoptera	1	
4/9/2011	1	CS	2	Stylogomphus albistylus	Gomphidae	Odonata	3	
4/9/2011	1	CS	2	Isoperla signata	Perlodidae	Plecoptera	1	
4/9/2011	1	CS	2	Isoperla signata	Perlodidae	Plecoptera	2	L/R
4/9/2011	1	CS	2	Isonychia bicolor	Isonychiidae	Ephemeroptera	5	
4/9/2011	1	CS	2	Polypedilum fallax	Chironominae	Diptera	1	
4/9/2011	1	CS	2	Fallceon sp.	Baetidae	Ephemeroptera	5	
4/9/2011	1	CS	2	Stenelmis sp.	Elmidae	Coleoptera	1	L/R
4/9/2011	1	CS	2	Helopicus natalus	Perlodidae	Plecoptera	1	L/R
4/9/2011	1	CS	2	Isonychia bicolor	Isonychiidae	Ephemeroptera	1	L/R
4/9/2011	1	CS	2	Helicopsyche sp.	Helicopsychidae	Tricoptera	52	
4/9/2011	1	CS	2	Argia sp.	Coengrionidae	Odonata	2	
4/9/2011	1	CS	2	Agapetus sp.	Glossosomatidae	Tricoptera	1	L/R
4/9/2011	1	CS	2	Chironomidae	Chironomidae	Diptera	1	
4/9/2011	1	CS	2	Hemerodromia sp.	Empididae	Diptera	1	
4/9/2011	1	CS	2	Rheotanytarsus sp.	Chironominae	Diptera	5	
4/9/2011	1	CS	2	Polypedilum (sp. A)	Chironominae	Diptera	21	
4/9/2011	1	CS	2	Asellidae	Asellidae	Isopoda	1	
4/9/2011	1	CS	2	Paratanytarsus sp.	Chironominae	Diptera	2	
4/9/2011	1	CS	2	Thienemannimyia grp	Tanypodinae	Diptera	8	
4/9/2011	1	CS	2	Bezzia sp.	Ceratopogonidae	Diptera	1	
4/9/2011	1	CS	2	Argia sp.	Coengrionidae	Odonata	1	L/R
4/9/2011	1	CS	2	Orthocladius sp. (yellow dome tooth)	Orthocladiinae	Diptera	3	
4/9/2011	1	CS	2	Nanocladius sp.	Orthocladiinae	Diptera	5	
4/9/2011	1	CS	2	Chimarra sp.	Philopotamidae	Tricoptera	1	
4/9/2011	1	CS	2	Eukiefferiella sp.	Orthocladiinae	Diptera	3	
4/9/2011	1	CS	2	Agnetina flavescens	Perlidae	Plecoptera	5	L/R
4/9/2011	1	CS	2	Chironomidae	Chironomidae	Diptera	97	
4/9/2011	1	CS	2	Elimia potosensis	Pleuroceridae	Gastropoda	1	
4/9/2011	1	CS	2	Chironomidae Pupae	Chironomidae	Diptera	11	
4/9/2011	1	CS	2	Corydalis sp.	Corydalidae	Megaloptera	5	L/R
4/9/2011	1	CS	2	Tabanus sp.	Tabanidae	Diptera	1	L/R
4/9/2011	1	CS	2	Cricotopus bicinctus	Orthocladiinae	Diptera	1	

S1 Appendix. Raw benthic macroinvertebrate data from Crane Pond Creek, Iron County, Missouri, USA.

4/9/2011	1	CS	2	Agnetina flavescens	Perlidae	Plecoptera	1	
4/9/2011	1	CS	2	Cheumatopsyche sp.	Hydropsychidae	Tricoptera	12	
4/9/2011	1	CS	2	Polypedilum convictum	Chironominae	Diptera	29	
4/9/2011	1	CS	2	Ablabesmyia sp.	Tanypodinae	Diptera	1	
4/9/2011	1	CS	2	Acarina sp.	Hydracarina	Arachnoidea	31	
4/9/2011	1	CS	2	Elimia potosensis	Pleuroceridae	Gastropoda	20	L/R
4/9/2011	1	CS	2	Eukiefferiella claripennis grp	Orthocladiinae	Diptera	1	
4/9/2011	1	CS	2	Orconectes hylas	Cambaridae	Decapoda	8	L/R
4/9/2011	1	CS	2	Perlodidae	Perlodidae	Plecoptera	1	
4/9/2011	1	CS	2	Orconectes luteus	Cambaridae	Decapoda	1	L/R
4/9/2011	1	CS	2	Simulium sp.	Simuliidae	Diptera	1	
4/9/2011	1	CS	2	Tanytarsus sp.	Chironominae	Diptera	3	
4/9/2011	1	CS	2	Oecetis sp.	Leptoceridae	Tricoptera	6	
4/9/2011	1	CS	2	Neoperla osage	Perlidae	Plecoptera	7	
4/9/2011	1	CS	2	Optioservus sp.	Elmidae	Coleoptera	11	
4/9/2011	1	CS	2	Thienemanniella sp.	Orthocladiinae	Diptera	1	
4/9/2011	1	CS	2	Maccaffertium sp.	Heptageniidae	Ephemeroptera	9	
4/9/2011	1	CS	2	Psephenus herricki	Psephenidae	Coleoptera	40	
4/9/2011	1	CS	2	Maccaffertium mediopunctatum	Heptageniidae	Ephemeroptera	2	L/R
4/9/2011	1	CS	2	Stenelmis sp.	Elmidae	Coleoptera	126	
4/9/2011	1	CS	2	Maccaffertium mediopunctatum	Heptageniidae	Ephemeroptera	10	
4/9/2011	1	CS	2	Optioservus sandersoni	Elmidae	Coleoptera	1	
4/9/2011	1	CS	2	Stenelmis lateralis	Elmidae	Coleoptera	13	
4/9/2011	1	CS	2	Orconectes hylas	Cambaridae	Decapoda	2	
4/9/2011	1	CS	3	Ephemerella sp.	Ephemerellidae	Ephemeroptera	3	
4/9/2011	1	CS	3	Gordiidae	Gordiidae	Gordioidea	1	L/R
4/9/2011	1	CS	3	Optioservus sp.	Elmidae	Coleoptera	12	
4/9/2011	1	CS	3	Oecetis sp.	Leptoceridae	Tricoptera	2	
4/9/2011	1	CS	3	Lumbriculidae	Lumbriculidae	Lumbriculida	2	
4/9/2011	1	CS	3	Chironomidae	Chironomidae	Diptera	174	
4/9/2011	1	CS	3	Chaetocladius sp.	Orthocladiinae	Diptera	3	
4/9/2011	1	CS	3	Tabanus sp.	Tabanidae	Diptera	2	L/R
4/9/2011	1	CS	3	Neoperla sp.	Perlidae	Plecoptera	2	
4/9/2011	1	CS	3	Cheumatopsyche sp.	Hydropsychidae	Tricoptera	1	
4/9/2011	1	CS	3	Eukiefferiella claripennis grp	Orthocladiinae	Diptera	15	
4/9/2011	1	CS	3	Thienemannimyia grp	Tanypodinae	Diptera	3	
4/9/2011	1	CS	3	Chimarra sp.	Philopotamidae	Tricoptera	7	

S1 Appendix. Raw benthic macroinvertebrate data from Crane Pond Creek, Iron County, Missouri, USA.

4/9/2011	1	CS	3	Helicopsyche sp.	Helicopsychidae	Tricoptera	10	
4/9/2011	1	CS	3	Eurylophella sp.	Ephemerellidae	Ephemeroptera	2	
4/9/2011	1	CS	3	Cricotopus isocladius	Orthocladiinae	Diptera	2	
4/9/2011	1	CS	3	Chironomus sp.	Chironominae	Diptera	1	
4/9/2011	1	CS	3	Chironomidae	Chironomidae	Diptera	1	
4/9/2011	1	CS	3	Polypedilum convictum	Chironominae	Diptera	56	
4/9/2011	1	CS	3	Corydalis sp.	Corydalidae	Megaloptera	3	
4/9/2011	1	CS	3	Agnetina flavescens	Perlidae	Plecoptera	1	L/R
4/9/2011	1	CS	3	Orconectes hylas	Cambaridae	Decapoda	2	
4/9/2011	1	CS	3	Orconectes hylas	Cambaridae	Decapoda	2	L/R
4/9/2011	1	CS	3	Corydalis sp.	Corydalidae	Megaloptera	2	
4/9/2011	1	CS	3	Tabanus sp.	Tabanidae	Diptera	1	
4/9/2011	1	CS	3	Tabanus sp.	Tabanidae	Diptera	2	
4/9/2011	1	CS	3	Orconectes hylas	Cambaridae	Decapoda	4	
4/9/2011	1	CS	3	Agnetina flavescens	Perlidae	Plecoptera	4	
4/9/2011	1	CS	3	Tipula sp.	Tipulidae	Diptera	1	L/R
4/9/2011	1	CS	3	Cricotopus bicinctus	Orthocladiinae	Diptera	1	
4/9/2011	1	CS	3	Stenelmis sp.	Elmidae	Coleoptera	255	
4/9/2011	1	CS	3	Plecoptera		Plecoptera	5	
4/9/2011	1	CS	3	Tricoptera		Tricoptera	1	
4/9/2011	1	CS	3	Orthocladius sp. (yellow dome tooth)	Orthocladiinae	Diptera	4	
4/9/2011	1	CS	3	Simulium sp.	Simuliidae	Diptera	32	
4/9/2011	1	CS	3	Maccaffertium sp.	Heptageniidae	Ephemeroptera	15	
4/9/2011	1	CS	3	Heptageniidae	Heptageniidae	Ephemeroptera	12	
4/9/2011	1	CS	3	Acarina sp.	Hydracarina	Arachnoidea	6	
4/9/2011	1	CS	3	Isonychia bicolor	Isonychiidae	Ephemeroptera	11	
4/9/2011	1	CS	3	Rheocricotopus sp.	Orthocladiinae	Diptera	3	
4/9/2011	1	CS	3	Isoperla namata	Perlodidae	Plecoptera	1	
4/9/2011	1	CS	3	Isoperla signata	Perlodidae	Plecoptera	5	
4/9/2011	1	CS	3	Polycentropus sp.	Polycentropodidae	Tricoptera	1	
4/9/2011	1	CS	3	Elimia potosensis	Pleuroceridae	Gastropoda	2	L/R
4/9/2011	1	CS	3	Elimia potosensis	Pleuroceridae	Gastropoda	6	
4/9/2011	1	CS	3	Isoperla sp.	Perlodidae	Plecoptera	1	
4/9/2011	1	CS	3	Isoperla sp.	Perlodidae	Plecoptera	2	
4/9/2011	1	CS	3	Stenelmis sp.	Elmidae	Coleoptera	8	L/R
4/9/2011	1	CS	3	Neoperla osage	Perlidae	Plecoptera	6	
4/9/2011	1	CS	3	Polypedilum (sp. A)	Chironominae	Diptera	48	

S1 Appendix. Raw benthic macroinvertebrate data from Crane Pond Creek, Iron County, Missouri, USA.

4/9/2011	1	CS	3	Psephenus herricki	Psephenidae	Coleoptera	6	
4/9/2011	1	CS	3	Neoperla osage	Perlidae	Plecoptera	1	
4/9/2011	1	CS	3	Fallceon sp.	Baetidae	Ephemeroptera	12	
4/9/2011	1	CS	3	Helopicus natalus	Perlodidae	Plecoptera	1	
4/9/2011	1	CS	3	Cheumatopsyche sp.	Hydropsychidae	Tricoptera	24	
4/9/2011	1	CS	3	Nanocladius sp.	Orthocladiinae	Diptera	1	
4/9/2011	1	CS	3	Paratanytarsus sp.	Chironominae	Diptera	1	
4/9/2011	1	CS	3	Thienemanniella sp.	Orthocladiinae	Diptera	2	
4/9/2011	1	CS	3	Rheotanytarsus sp.	Chironominae	Diptera	3	
4/9/2011	1	NF	1	Psephenus herricki	Psephenidae	Coleoptera	1	L/R
4/9/2011	1	NF	1	Parakiefferiella sp.	Orthocladiinae	Diptera	22	
4/9/2011	1	NF	1	Tricoptera		Tricoptera	1	
4/9/2011	1	NF	1	Paratanytarsus sp.	Chironominae	Diptera	1	
4/9/2011	1	NF	1	Microtendipes sp.	Chironominae	Diptera	7	
4/9/2011	1	NF	1	Microchironomus sp.	Chironominae	Diptera	1	(?)
4/9/2011	1	NF	1	Cricotopus bicinctus	Orthocladiinae	Diptera	9	
4/9/2011	1	NF	1	Dicrotendipes sp.	Chironominae	Diptera	3	
4/9/2011	1	NF	1	Argia sp.	Coengrionidae	Odonata	2	
4/9/2011	1	NF	1	Elimia potosensis	Pleuroceridae	Gastropoda	1	L/R
4/9/2011	1	NF	1	Chimarra sp.	Philopotamidae	Tricoptera	1	L/R
4/9/2011	1	NF	1	Chironomidae	Chironomidae	Diptera	282	
4/9/2011	1	NF	1	Thienemannimyia grp	Tanypodinae	Diptera	20	
4/9/2011	1	NF	1	Gyraulus sp.	Planorbidae	Gastropoda	1	
4/9/2011	1	NF	1	Bezzia sp.	Ceratopogonidae	Diptera	9	
4/9/2011	1	NF	1	Orthocladius sp. (yellow dome tooth)	Orthocladiinae	Diptera	23	
4/9/2011	1	NF	1	Lauterborniella sp.	Chironominae	Diptera	11	
4/9/2011	1	NF	1	Neoperla sp.	Perlidae	Plecoptera	3	
4/9/2011	1	NF	1	Polypedilum illinoense	Chironominae	Diptera	1	
4/9/2011	1	NF	1	Ablabesmyia sp.	Tanypodinae	Diptera	3	
4/9/2011	1	NF	1	Mystacides sp.	Leptoceridae	Tricoptera	1	
4/9/2011	1	NF	1	Stenonema femoratum	Heptageniidae	Ephemeroptera	1	
4/9/2011	1	NF	1	Stenonema femoratum	Heptageniidae	Ephemeroptera	2	L/R
4/9/2011	1	NF	1	Chaetocladius sp.	Orthocladiinae	Diptera	1	
4/9/2011	1	NF	1	Eurylophella sp.	Ephemerellidae	Ephemeroptera	2	
4/9/2011	1	NF	1	Stenelmis sp.	Elmidae	Coleoptera	14	
4/9/2011	1	NF	1	Thienemanniella sp.	Orthocladiinae	Diptera	3	
4/9/2011	1	NF	1	Neoperla falayah	Perlidae	Plecoptera	1	L/R

S1 Appendix. Raw benthic macroinvertebrate data from Crane Pond Creek, Iron County, Missouri, USA.

4/9/2011	1	NF	1	Optioservus sp.	Elmidae	Coleoptera	2	
4/9/2011	1	NF	1	Hemerodromia sp.	Empididae	Diptera	2	
4/9/2011	1	NF	1	Acarina sp.	Hydracarina	Arachnoidea	1	
4/9/2011	1	NF	1	Cladotanytarsus sp.	Chironominae	Diptera	28	
4/9/2011	1	NF	1	Helicopsyche sp.	Helicopsychidae	Tricoptera	2	
4/9/2011	1	NF	1	Tanytarsus sp.	Chironominae	Diptera	29	
4/9/2011	1	NF	2	Neoperla sp.	Perlidae	Plecoptera	1	
4/9/2011	1	NF	2	Chironomidae	Chironomidae	Diptera	151	
4/9/2011	1	NF	2	Lauterborniella sp.	Chironominae	Diptera	5	
4/9/2011	1	NF	2	Amphiagrion sp.	Coengrionidae	Odonata	1	L/R
4/9/2011	1	NF	2	Argia sp.	Coengrionidae	Odonata	1	
4/9/2011	1	NF	2	Thienemannimyia grp	Tanypodinae	Diptera	11	
4/9/2011	1	NF	2	Microtendipes sp.	Chironominae	Diptera	9	
4/9/2011	1	NF	2	Perlidae	Perlidae	Plecoptera	1	
4/9/2011	1	NF	2	Perlodidae	Perlodidae	Plecoptera	1	
4/9/2011	1	NF	2	Stenonema femoratum	Heptageniidae	Ephemeroptera	4	L/R
4/9/2011	1	NF	2	Procladius sp.	Tanypodinae	Diptera	1	
4/9/2011	1	NF	2	Elimia potosensis	Pleuroceridae	Gastropoda	1	
4/9/2011	1	NF	2	Fallceon sp.	Baetidae	Ephemeroptera	1	
4/9/2011	1	NF	2	Stenonema femoratum	Heptageniidae	Ephemeroptera	4	
4/9/2011	1	NF	2	Helichus sp.	Dryopidae	Coleoptera	1	
4/9/2011	1	NF	2	Orthocladius sp. (yellow dome tooth)	Orthocladiinae	Diptera	41	
4/9/2011	1	NF	2	Dicrotendipes sp.	Chironominae	Diptera	2	
4/9/2011	1	NF	2	Lype diversa	Psychomyiidae	Tricoptera	1	
4/9/2011	1	NF	2	Cricotopus isocladius	Orthocladiinae	Diptera	3	
4/9/2011	1	NF	2	Thienemanniella sp.	Orthocladiinae	Diptera	1	
4/9/2011	1	NF	2	Parakiefferiella sp.	Orthocladiinae	Diptera	6	
4/9/2011	1	NF	2	Tabanus sp.	Tabanidae	Diptera	2	
4/9/2011	1	NF	2	Stenelmis sp.	Elmidae	Coleoptera	75	
4/9/2011	1	NF	2	Phaenopsectra sp.	Chironominae	Diptera	2	
4/9/2011	1	NF	2	Dubiraphia sp.	Elmidae	Coleoptera	6	
4/9/2011	1	NF	2	Psephenus herricki	Psephenidae	Coleoptera	2	
4/9/2011	1	NF	2	Ablabesmyia sp.	Tanypodinae	Diptera	13	
4/9/2011	1	NF	2	Oecetis sp.	Leptoceridae	Tricoptera	2	
4/9/2011	1	NF	2	Cladotanytarsus sp.	Chironominae	Diptera	35	
4/9/2011	1	NF	2	Stylogomphus albistylus	Gomphidae	Odonata	1	
4/9/2011	1	NF	2	Psephenus herricki	Psephenidae	Coleoptera	1	L/R

S1 Appendix. Raw benthic macroinvertebrate data from Crane Pond Creek, Iron County, Missouri, USA.

4/9/2011	1	NF	2	Tanytarsus sp.	Chironominae	Diptera	22	
4/9/2011	1	NF	2	Paratanytarsus sp.	Chironominae	Diptera	1	
4/9/2011	1	NF	2	Eurylophella sp.	Ephemerellidae	Ephemeroptera	4	
4/9/2011	1	NF	2	Acarina sp.	Hydracarina	Arachnoidea	5	
4/9/2011	1	NF	2	Cricotopus bicinctus	Orthocladiinae	Diptera	13	
4/9/2011	1	NF	3	Orthocladius sp. (yellow dome tooth)	Orthocladiinae	Diptera	68	
4/9/2011	1	NF	3	Stenelmis sp.	Elmidae	Coleoptera	58	
4/9/2011	1	NF	3	Baetisca sp.	Baetiscidae	Ephemeroptera	1	
4/9/2011	1	NF	3	Psephenus herricki	Psephenidae	Coleoptera	2	L/R
4/9/2011	1	NF	3	Helicopsyche sp.	Helicopsychidae	Tricoptera	1	
4/9/2011	1	NF	3	Polycentropodidae	Polycentropodidae	Tricoptera	3	
4/9/2011	1	NF	3	Parakiefferiella sp.	Orthocladiinae	Diptera	4	
4/9/2011	1	NF	3	Psephenus herricki	Psephenidae	Coleoptera	3	
4/9/2011	1	NF	3	Cricotopus bicinctus	Orthocladiinae	Diptera	19	
4/9/2011	1	NF	3	Chironomus sp.	Chironominae	Diptera	1	
4/9/2011	1	NF	3	Cricotopus isocladius	Orthocladiinae	Diptera	1	
4/9/2011	1	NF	3	Tabanus sp.	Tabanidae	Diptera	2	
4/9/2011	1	NF	3	Dubiraphia sp.	Elmidae	Coleoptera	7	
4/9/2011	1	NF	3	Ablabesmyia sp.	Tanypodinae	Diptera	12	
4/9/2011	1	NF	3	Stylogomphus albistylus	Gomphidae	Odonata	1	L/R
4/9/2011	1	NF	3	Stylogomphus albistylus	Gomphidae	Odonata	1	
4/9/2011	1	NF	3	Eurylophella sp.	Ephemerellidae	Ephemeroptera	6	
4/9/2011	1	NF	3	Ephemerellidae	Ephemerellidae	Ephemeroptera	3	
4/9/2011	1	NF	3	Stenonema femoratum	Heptageniidae	Ephemeroptera	11	
4/9/2011	1	NF	3	Eukiefferiella sp.	Orthocladiinae	Diptera	2	
4/9/2011	1	NF	3	Chironomidae	Chironomidae	Diptera	228	
4/9/2011	1	NF	3	Perlidae	Perlidae	Plecoptera	2	
4/9/2011	1	NF	3	Cheumatopsyche sp.	Hydropsychidae	Tricoptera	3	
4/9/2011	1	NF	3	Lauterborniella sp.	Chironominae	Diptera	5	
4/9/2011	1	NF	3	Cladotanytarsus sp.	Chironominae	Diptera	14	
4/9/2011	1	NF	3	Stenonema femoratum	Heptageniidae	Ephemeroptera	3	L/R
4/9/2011	1	NF	3	Eurylophella sp.	Ephemerellidae	Ephemeroptera	2	L/R
4/9/2011	1	NF	3	Microtendipes sp.	Chironominae	Diptera	5	
4/9/2011	1	NF	3	Dicrotendipes sp.	Chironominae	Diptera	3	
4/9/2011	1	NF	3	Tanytarsus sp.	Chironominae	Diptera	6	
4/9/2011	1	NF	3	Argia sp.	Coengrionidae	Odonata	1	L/R
4/9/2011	1	NF	3	Polypedilum convictum	Chironominae	Diptera	1	

S1 Appendix. Raw benthic macroinvertebrate data from Crane Pond Creek, Iron County, Missouri, USA.

4/9/2011	1	NF	3	Agnetina flavescens	Perlidae	Plecoptera	1	
4/9/2011	1	NF	3	Acarina sp.	Hydracarina	Arachnoidea	2	
4/9/2011	1	NF	3	Dashyheleinae sp.	Ceratopogonidae	Diptera	1	
4/9/2011	1	NF	3	Polypedilum (sp. A)	Chironominae	Diptera	1	
4/9/2011	1	NF	3	Thienemannimyia grp	Tanypodinae	Diptera	15	
4/10/2011	1	NF	3	Gyraulus sp.	Planorbidae	Gastropoda	1	
4/10/2011	1	NF	3	Tubificidae	Tubificidae	Tubificida	1	
4/10/2011	2	CS	1	Muscidae	Muscidae	Diptera	1	
4/10/2011	2	CS	1	Tricoptera Pupa		Tricoptera	4	
4/10/2011	2	CS	1	Thienemannimyia grp	Tanypodinae	Diptera	40	
4/10/2011	2	CS	1	Polypedilum convictum	Chironominae	Diptera	30	
4/10/2011	2	CS	1	Optioservus sp.	Elmidae	Coleoptera	6	
4/10/2011	2	CS	1	Oecetis sp.	Leptoceridae	Tricoptera	4	
4/10/2011	2	CS	1	Neoperla sp.	Perlidae	Plecoptera	1	L/R
4/10/2011	2	CS	1	Chimarra sp.	Philopotamidae	Tricoptera	9	
4/10/2011	2	CS	1	Chaetocladius sp.	Orthocladiinae	Diptera	4	
4/10/2011	2	CS	1	Ectopria nervosa	Psephenidae	Coleoptera	1	
4/10/2011	2	CS	1	Chironomus sp.	Chironominae	Diptera	2	
4/10/2011	2	CS	1	Amphinemura sp.	Nemouridae	Plecoptera	2	
4/10/2011	2	CS	1	Tanytarsus sp.	Chironominae	Diptera	3	
4/10/2011	2	CS	1	Agnetina flavescens	Perlidae	Plecoptera	2	
4/10/2011	2	CS	1	Maccaffertium sp.	Heptageniidae	Ephemeroptera	35	
4/10/2011	2	CS	1	Acarina sp.	Hydracarina	Arachnoidea	14	
4/10/2011	2	CS	1	Maccaffertium mediopunctatum	Heptageniidae	Ephemeroptera	3	L/R
4/10/2011	2	CS	1	Nanocladius sp.	Orthocladiinae	Diptera	1	
4/10/2011	2	CS	1	Cambarus hubbsi	Cambaridae	Decapoda	2	
4/10/2011	2	CS	1	Maccaffertium mediopunctatum	Heptageniidae	Ephemeroptera	1	
4/10/2011	2	CS	1	Nilotanypus sp.	Tanypodinae	Diptera	1	
4/10/2011	2	CS	1	Neoperla sp.	Perlidae	Plecoptera	6	
4/10/2011	2	CS	1	Petrophilia sp.	Pyralidae	Lepidoptera	3	
4/10/2011	2	CS	1	Cricotopus isocladius	Orthocladiinae	Diptera	8	
4/10/2011	2	CS	1	Thienemanniella sp.	Orthocladiinae	Diptera	4	
4/10/2011	2	CS	1	Polypedilum (sp. A)	Chironominae	Diptera	63	
4/10/2011	2	CS	1	Stenelmis sp.	Elmidae	Coleoptera	88	
4/10/2011	2	CS	1	Stenelmis lateralis	Elmidae	Coleoptera	9	
4/10/2011	2	CS	1	Hemerodromia sp.	Empididae	Diptera	11	
4/10/2011	2	CS	1	Corydalis sp.	Corydalidae	Megaloptera	2	L/R

S1 Appendix. Raw benthic macroinvertebrate data from Crane Pond Creek, Iron County, Missouri, USA.

4/10/2011	2	CS	1	Psephenus herricki	Psephenidae	Coleoptera	8	
4/10/2011	2	CS	1	Psephenus herricki	Psephenidae	Coleoptera	2	L/R
4/10/2011	2	CS	1	Cricotopus bicinctus	Orthocladiinae	Diptera	20	
4/10/2011	2	CS	1	Tipula sp.	Tipulidae	Diptera	2	L/R
4/10/2011	2	CS	1	Cheumatopsyche sp.	Hydropsychidae	Tricoptera	2	L/R
4/10/2011	2	CS	1	Chironomidae	Chironomidae	Diptera	293	
4/10/2011	2	CS	1	Agnetina flavescens	Perlidae	Plecoptera	2	L/R
4/10/2011	2	CS	1	Cheumatopsyche sp.	Hydropsychidae	Tricoptera	23	
4/10/2011	2	CS	1	Petrophilia sp.	Pyralidae	Lepidoptera	1	L/R
4/10/2011	2	CS	1	Orconectes peruncus	Cambaridae	Decapoda	6	
4/10/2011	2	CS	1	Orconectes luteus	Cambaridae	Decapoda	3	
4/10/2011	2	CS	1	Phaenopsectra sp.	Chironominae	Diptera	1	
4/10/2011	2	CS	1	Stylogomphus albistylus	Gomphidae	Odonata	2	
4/10/2011	2	CS	1	Eukiefferiella sp.	Orthocladiinae	Diptera	16	
4/10/2011	2	CS	1	Ochrotrichia sp.	Hydroptilidae	Tricoptera	3	
4/10/2011	2	CS	1	Tipula sp.	Tipulidae	Diptera	1	
4/10/2011	2	CS	1	Isoperla sp.	Perlodidae	Plecoptera	11	
4/10/2011	2	CS	1	Physella sp.	Physidae	Gastropoda	2	L/R
4/10/2011	2	CS	1	Agapetus sp.	Glossosomatidae	Tricoptera	1	L/R
4/10/2011	2	CS	1	Helicopsyche sp.	Helicopsychidae	Tricoptera	32	
4/10/2011	2	CS	1	Eurylophella sp.	Ephemerellidae	Ephemeroptera	4	
4/10/2011	2	CS	1	Isonychia bicolor	Isonychiidae	Ephemeroptera	8	
4/10/2011	2	CS	1	Argia sp.	Coengrionidae	Odonata	10	
4/10/2011	2	CS	1	Calopteryx sp.	Calopterygidae	Odonata	1	L/R
4/10/2011	2	CS	1	Baetidae	Baetidae	Ephemeroptera	5	
4/10/2011	2	CS	1	Argia sp.	Coengrionidae	Odonata	1	L/R
4/10/2011	2	CS	1	Rheocricotopus sp.	Orthocladiinae	Diptera	21	
4/10/2011	2	CS	1	Isonychia bicolor	Isonychiidae	Ephemeroptera	3	L/R
4/10/2011	2	CS	1	Haploperla brevius	Chloroperlidae	Plecoptera	8	
4/10/2011	2	CS	1	Orthocladius sp. (yellow dome tooth)	Orthocladiinae	Diptera	27	
4/10/2011	2	CS	1	Paratanytarsus sp.	Chironominae	Diptera	7	
4/10/2011	2	CS	1	Parachironomus sp.	Chironominae	Diptera	1	
4/10/2011	2	CS	1	Fallceon sp.	Baetidae	Ephemeroptera	1	
4/10/2011	2	CS	1	Baetis sp.	Baetidae	Ephemeroptera	5	
4/10/2011	2	CS	1	Hemerodromia sp.	Empididae	Diptera	2	
4/10/2011	2	CS	1	Rheotanytarsus sp.	Chironominae	Diptera	7	
4/10/2011	2	CS	2	Lumbriculidae	Lumbriculidae	Lumbriculida	2	

S1 Appendix. Raw benthic macroinvertebrate data from Crane Pond Creek, Iron County, Missouri, USA.

4/10/2011	2	CS	2	Haploperla brevius	Chloroperlidae	Plecoptera	13	
4/10/2011	2	CS	2	Asellidae	Asellidae	Isopoda	1	
4/10/2011	2	CS	2	Stylogomphus albistylus	Gomphidae	Odonata	4	
4/10/2011	2	CS	2	Cladotanytarsus sp.	Chironominae	Diptera	2	
4/10/2011	2	CS	2	Hemerodromia sp.	Empididae	Diptera	7	
4/10/2011	2	CS	2	Rheocricotopus sp.	Orthocladiinae	Diptera	34	
4/10/2011	2	CS	2	Tabanus sp.	Tabanidae	Diptera	2	
4/10/2011	2	CS	2	Acroneuria frisoni	Perlidae	Plecoptera	1	
4/10/2011	2	CS	2	Polypedilum (sp. A)	Chironominae	Diptera	118	
4/10/2011	2	CS	2	Simulium sp.	Simuliidae	Diptera	13	
4/10/2011	2	CS	2	Physella sp.	Physidae	Gastropoda	2	L/R
4/10/2011	2	CS	2	Optioservus sp.	Elmidae	Coleoptera	7	
4/10/2011	2	CS	2	Polypedilum convictum	Chironominae	Diptera	40	
4/10/2011	2	CS	2	Paratanytarsus sp.	Chironominae	Diptera	1	
4/10/2011	2	CS	2	Chironomidae	Chironomidae	Diptera	346	
4/10/2011	2	CS	2	Thienemannimyia grp	Tanypodinae	Diptera	15	
4/10/2011	2	CS	2	Tabanus sp.	Tabanidae	Diptera	2	L/R
4/10/2011	2	CS	2	Cricotopus bicinctus	Orthocladiinae	Diptera	18	
4/10/2011	2	CS	2	Corydalis sp.	Corydalidae	Megaloptera	2	L/R
4/10/2011	2	CS	2	Helicopsyche sp.	Helicopsychidae	Tricoptera	4	
4/10/2011	2	CS	2	Petrophilia sp.	Pyralidae	Lepidoptera	1	
4/10/2011	2	CS	2	Cheumatopsyche sp.	Hydropsychidae	Tricoptera	51	
4/10/2011	2	CS	2	Petrophilia sp.	Pyralidae	Lepidoptera	1	L/R
4/10/2011	2	CS	2	Orconectes luteus	Cambaridae	Decapoda	1	L/R
4/10/2011	2	CS	2	Tubicidae	Tubicidae	Tubicida	1	L/R
4/10/2011	2	CS	2	Psephenus herricki	Psephenidae	Coleoptera	1	L/R
4/10/2011	2	CS	2	Stenelmis sp.	Elmidae	Coleoptera	79	
4/10/2011	2	CS	2	Psephenus herricki	Psephenidae	Coleoptera	6	
4/10/2011	2	CS	2	Stenelmis lateralis	Elmidae	Coleoptera	8	
4/10/2011	2	CS	2	Thienemanniella sp.	Orthocladiinae	Diptera	2	
4/10/2011	2	CS	2	Agnetina flavescens	Perlidae	Plecoptera	1	L/R
4/10/2011	2	CS	2	Orconectes peruncus	Cambaridae	Decapoda	4	L/R
4/10/2011	2	CS	2	Corydalis sp.	Corydalidae	Megaloptera	2	
4/10/2011	2	CS	2	Rheotanytarsus sp.	Chironominae	Diptera	11	
4/10/2011	2	CS	2	Maccaffertium mediopunctatum	Heptageniidae	Ephemeroptera	1	L/R
4/10/2011	2	CS	2	Maccaffertium sp.	Heptageniidae	Ephemeroptera	55	
4/10/2011	2	CS	2	Eukiefferiella sp.	Orthocladiinae	Diptera	14	

S1 Appendix. Raw benthic macroinvertebrate data from Crane Pond Creek, Iron County, Missouri, USA.

4/10/2011	2	CS	2	Lauterborniella sp.	Chironominae	Diptera	3	
4/10/2011	2	CS	2	Maccaffertium mediopunctatum	Heptageniidae	Ephemeroptera	2	
4/10/2011	2	CS	2	Acarina sp.	Hydracarina	Arachnoidea	14	
4/10/2011	2	CS	2	Ephemerella sp.	Ephemerellidae	Ephemeroptera	1	
4/10/2011	2	CS	2	Neoperla sp.	Perlidae	Plecoptera	1	L/R
4/10/2011	2	CS	2	Neoperla sp.	Perlidae	Plecoptera	3	
4/10/2011	2	CS	2	Tanytarsus sp.	Chironominae	Diptera	9	
4/10/2011	2	CS	2	Nanocladius sp.	Orthocladiinae	Diptera	12	
4/10/2011	2	CS	2	Acroneuria frisoni	Perlidae	Plecoptera	1	L/R
4/10/2011	2	CS	2	Isoperla sp.	Perlodidae	Plecoptera	1	L/R
4/10/2011	2	CS	2	Chaetocladius sp.	Orthocladiinae	Diptera	2	
4/10/2011	2	CS	2	Orthocladius sp. (yellow dome tooth)	Orthocladiinae	Diptera	24	
4/10/2011	2	CS	2	Amphinemura sp.	Nemouridae	Plecoptera	14	
4/10/2011	2	CS	2	Tipula sp.	Tipulidae	Diptera	1	L/R
4/10/2011	2	CS	2	Glossiphoniidae	Rhynchobdellida	Hirudinea	1	
4/10/2011	2	CS	2	Chimarra sp.	Philopotamidae	Tricoptera	36	
4/10/2011	2	CS	2	Tipula sp.	Tipulidae	Diptera	1	
4/10/2011	2	CS	2	Cricotopus isocladius	Orthocladiinae	Diptera	8	
4/10/2011	2	CS	2	Isonychia bicolor	Isonychiidae	Ephemeroptera	30	
4/10/2011	2	CS	2	Cambarus hubbsi	Cambaridae	Decapoda	1	L/R
4/10/2011	2	CS	2	Agapetus sp.	Glossosomatidae	Tricoptera	1	
4/10/2011	2	CS	2	Fallceon sp.	Baetidae	Ephemeroptera	17	
4/10/2011	2	CS	3	Psephenus herricki	Psephenidae	Coleoptera	2	L/R
4/10/2011	2	CS	3	Plauditus sp.	Baetidae	Ephemeroptera	1	
4/10/2011	2	CS	3	Psephenus herricki	Psephenidae	Coleoptera	3	
4/10/2011	2	CS	3	Maccaffertium sp.	Heptageniidae	Ephemeroptera	60	
4/10/2011	2	CS	3	Rheocricotopus sp.	Orthocladiinae	Diptera	41	
4/10/2011	2	CS	3	Acroneuria frisoni	Perlidae	Plecoptera	3	L/R
4/10/2011	2	CS	3	Rheotanytarsus sp.	Chironominae	Diptera	16	
4/10/2011	2	CS	3	Sialis sp.	Sialidae	Megaloptera	1	
4/10/2011	2	CS	3	Polypedilum (sp. A)	Chironominae	Diptera	90	
4/10/2011	2	CS	3	Hemerodromia sp.	Empididae	Diptera	7	
4/10/2011	2	CS	3	Maccaffertium mediopunctatum	Heptageniidae	Ephemeroptera	6	L/R
4/10/2011	2	CS	3	Isoperla signata	Perlodidae	Plecoptera	1	L/R
4/10/2011	2	CS	3	Agapetus sp.	Glossosomatidae	Tricoptera	1	
4/10/2011	2	CS	3	Acarina sp.	Hydracarina	Arachnoidea	16	
4/10/2011	2	CS	3	Elmidae sp.	Elmidae	Coleoptera	1	Larvae

S1 Appendix. Raw benthic macroinvertebrate data from Crane Pond Creek, Iron County, Missouri, USA.

4/10/2011	2	CS	3	Lumbriculidae	Lumbriculidae	Lumbriculida	3	
4/10/2011	2	CS	3	Simulium sp. Pupa	Simuliidae	Diptera	1	
4/10/2011	2	CS	3	Helopicus natalus	Perlodidae	Plecoptera	1	L/R
4/10/2011	2	CS	3	Simulium sp.	Simuliidae	Diptera	4	
4/10/2011	2	CS	3	Cricotopus bicinctus	Orthocladiinae	Diptera	25	
4/10/2011	2	CS	3	Cladotanytarsus sp.	Chironominae	Diptera	2	
4/10/2011	2	CS	3	Chironomus sp.	Chironominae	Diptera	2	
4/10/2011	2	CS	3	Isonychia bicolor	Isonychiidae	Ephemeroptera	9	L/R
4/10/2011	2	CS	3	Polypedilum convictum	Chironominae	Diptera	61	
4/10/2011	2	CS	3	Ectopria nervosa	Psephenidae	Coleoptera	1	
4/10/2011	2	CS	3	Cheumatopsyche sp.	Hydropsychidae	Tricoptera	32	
4/10/2011	2	CS	3	Optioservus sandersoni	Elmidae	Coleoptera	6	
4/10/2011	2	CS	3	Haploperla brevius	Chloroperlidae	Plecoptera	2	L/R
4/10/2011	2	CS	3	Cheumatopsyche sp.	Hydropsychidae	Tricoptera	5	L/R
4/10/2011	2	CS	3	Optioservus sp.	Elmidae	Coleoptera	1	
4/10/2011	2	CS	3	Orconectes luteus	Cambaridae	Decapoda	1	L/R
4/10/2011	2	CS	3	Haploperla brevius	Chloroperlidae	Plecoptera	8	
4/10/2011	2	CS	3	Tanytarsus sp.	Chironominae	Diptera	7	
4/10/2011	2	CS	3	Thienemanniella sp.	Orthocladiinae	Diptera	3	
4/10/2011	2	CS	3	Serratella sp.	Ephemerellidae	Ephemeroptera	2	
4/10/2011	2	CS	3	Corydalis sp.	Corydalidae	Megaloptera	3	
4/10/2011	2	CS	3	Fallceon sp.	Baetidae	Ephemeroptera	6	
4/10/2011	2	CS	3	Petrophilia sp.	Pyralidae	Lepidoptera	2	
4/10/2011	2	CS	3	Eurylophella sp.	Ephemerellidae	Ephemeroptera	1	
4/10/2011	2	CS	3	Neoperla sp.	Perlidae	Plecoptera	3	L/R
4/10/2011	2	CS	3	Neoperla osage	Perlidae	Plecoptera	5	
4/10/2011	2	CS	3	Petrophilia sp.	Pyralidae	Lepidoptera	1	L/R
4/10/2011	2	CS	3	Eurylophella sp.	Ephemerellidae	Ephemeroptera	1	L/R
4/10/2011	2	CS	3	Isonychia bicolor	Isonychiidae	Ephemeroptera	9	
4/10/2011	2	CS	3	Helicopsyche sp.	Helicopsychidae	Tricoptera	9	
4/10/2011	2	CS	3	Maccaffertium sp.	Heptageniidae	Ephemeroptera	6	L/R
4/10/2011	2	CS	3	Isoperla decepta	Perlodidae	Plecoptera	7	L/R
4/10/2011	2	CS	3	Corydalis sp.	Corydalidae	Megaloptera	2	L/R
4/10/2011	2	CS	3	Chaetocladius sp.	Orthocladiinae	Diptera	6	
4/10/2011	2	CS	3	Antocha sp.	Tipulidae	Diptera	1	
4/10/2011	2	CS	3	Microtendipes sp.	Chironominae	Diptera	2	
4/10/2011	2	CS	3	Agnetina flavescens	Perlidae	Plecoptera	1	L/R

S1 Appendix. Raw benthic macroinvertebrate data from Crane Pond Creek, Iron County, Missouri, USA.

4/10/2011	2	CS	3	Tipula sp.	Tipulidae	Diptera	1	
4/10/2011	2	CS	3	Thienemannimyia grp	Tanypodinae	Diptera	13	
4/10/2011	2	CS	3	Nigronia sp.	Corydalidae	Megaloptera	1	L/R
4/10/2011	2	CS	3	Stylogomphus albistylus	Gomphidae	Odonata	12	
4/10/2011	2	CS	3	Nanocladius sp.	Orthocladiinae	Diptera	1	
4/10/2011	2	CS	3	Cambarus hubbsi	Cambaridae	Decapoda	1	L/R
4/10/2011	2	CS	3	Stenelmis lateralis	Elmidae	Coleoptera	7	
4/10/2011	2	CS	3	Amphinemura sp.	Nemouridae	Plecoptera	11	
4/10/2011	2	CS	3	Chimarra sp.	Philopotamidae	Tricoptera	15	
4/10/2011	2	CS	3	Eukiefferiella sp.	Orthocladiinae	Diptera	11	
4/10/2011	2	CS	3	Chironomidae	Chironomidae	Diptera	337	
4/10/2011	2	CS	3	Tricorythodes sp.	Leptohyphidae	Ephemeroptera	6	
4/10/2011	2	CS	3	Stenelmis sp.	Elmidae	Coleoptera	32	
4/10/2011	2	CS	3	Baetis sp.	Baetidae	Ephemeroptera	1	
4/10/2011	2	CS	3	Asellidae	Asellidae	Isopoda	1	L/R
4/10/2011	2	CS	3	Cricotopus isocladius	Orthocladiinae	Diptera	6	
4/10/2011	2	CS	3	Phaenopsectra sp.	Chironominae	Diptera	1	
4/10/2011	2	CS	3	Orthocladius sp. (yellow dome tooth)	Orthocladiinae	Diptera	13	
4/10/2011	2	CS	3	Polycentropus sp.	Polycentropodidae	Tricoptera	4	
4/10/2011	2	CS	3	Lauterborniella sp.	Chironominae	Diptera	1	
4/10/2011	2	CS	3	Tabanus sp.	Tabanidae	Diptera	2	L/R
4/10/2011	2	CS	3	Argia sp.	Coengrionidae	Odonata	3	
4/10/2011	2	CS	3	Stenelmis sp.	Elmidae	Coleoptera	2	L/R
4/10/2011	2	CS	3	Stenelmis sp.	Elmidae	Coleoptera	1	L/R
4/10/2011	2	CS	3	Tipula sp.	Tipulidae	Diptera	2	L/R
4/10/2011	2	NF	1	Eukiefferiella sp.	Orthocladiinae	Diptera	2	
4/10/2011	2	NF	1	Lauterborniella sp.	Chironominae	Diptera	15	
4/10/2011	2	NF	1	Optioservus sandersoni	Elmidae	Coleoptera	1	
4/10/2011	2	NF	1	Phaenopsectra sp.	Chironominae	Diptera	1	
4/10/2011	2	NF	1	Cladotanytarsus sp.	Chironominae	Diptera	93	
4/10/2011	2	NF	1	Polypedilum convictum	Chironominae	Diptera	1	
4/10/2011	2	NF	1	Chaetocladius sp.	Orthocladiinae	Diptera	1	
4/10/2011	2	NF	1	Tanytarsus sp.	Chironominae	Diptera	55	
4/10/2011	2	NF	1	Stylogomphus albistylus	Gomphidae	Odonata	1	L/R
4/10/2011	2	NF	1	Acarina sp.	Hydracarina	Arachnoidea	9	
4/10/2011	2	NF	1	Hydropsyche sp.	Hydropsychidae	Tricoptera	1	L/R
4/10/2011	2	NF	1	Rheocricotopus sp.	Orthocladiinae	Diptera	1	

S1 Appendix. Raw benthic macroinvertebrate data from Crane Pond Creek, Iron County, Missouri, USA.

4/10/2011	2	NF	1	Helicopsyche sp.	Helicopsychidae	Tricoptera	3	
4/10/2011	2	NF	1	Parakiefferiella sp.	Orthocladiinae	Diptera	4	
4/10/2011	2	NF	1	Haploperla brevius	Chloroperlidae	Plecoptera	2	
4/10/2011	2	NF	1	Bezzia sp.	Ceratopogonidae	Diptera	1	
4/10/2011	2	NF	1	Polypedilum illinoense	Chironominae	Diptera	2	
4/10/2011	2	NF	1	Eurylophella sp.	Ephemerellidae	Ephemeroptera	14	
4/10/2011	2	NF	1	Agapetus sp.	Glossosomatidae	Tricoptera	2	
4/10/2011	2	NF	1	Orthocladius sp. (yellow dome tooth)	Orthocladiinae	Diptera	35	
4/10/2011	2	NF	1	Tricorythodes sp.	Leptohyphidae	Ephemeroptera	15	
4/10/2011	2	NF	1	Nilotanypus sp.	Tanypodinae	Diptera	2	
4/10/2011	2	NF	1	Tanypus sp.	Tanypodinae	Diptera	2	
4/10/2011	2	NF	1	Paratanytarsus sp.	Chironominae	Diptera	1	
4/10/2011	2	NF	1	Ablabesmyia sp.	Tanypodinae	Diptera	14	
4/10/2011	2	NF	1	Ectopria nervosa	Psephenidae	Coleoptera	1	
4/10/2011	2	NF	1	Pycnopsyche sp.	Limnephilidae	Tricoptera	1	L/R
4/10/2011	2	NF	1	Microtendipes sp.	Chironominae	Diptera	8	
4/10/2011	2	NF	1	Ochrotrichia sp.	Hydroptilidae	Tricoptera	1	
4/10/2011	2	NF	1	Dubiraphia sp.	Elmidae	Coleoptera	1	
4/10/2011	2	NF	1	Eurylophella sp.	Ephemerellidae	Ephemeroptera	1	L/R
4/10/2011	2	NF	1	Gyraulus sp.	Planorbidae	Gastropoda	2	
4/10/2011	2	NF	1	Cricotopus isocladius	Orthocladiinae	Diptera	3	
4/10/2011	2	NF	1	Stenonema femoratum	Heptageniidae	Ephemeroptera	13	
4/10/2011	2	NF	1	Stenelmis sp.	Elmidae	Coleoptera	13	
4/10/2011	2	NF	1	Chironomidae	Chironomidae	Diptera	215	
4/10/2011	2	NF	1	Cryptochironomus sp.	Chironominae	Diptera	2	
4/10/2011	2	NF	1	Dicrotendipes sp.	Chironominae	Diptera	3	
4/10/2011	2	NF	1	Stenonema femoratum	Heptageniidae	Ephemeroptera	3	L/R
4/10/2011	2	NF	1	Thienemannimyia grp	Tanypodinae	Diptera	17	
4/10/2011	2	NF	1	Stenelmis lateralis	Elmidae	Coleoptera	2	
4/10/2011	2	NF	1	Cricotopus bicinctus	Orthocladiinae	Diptera	17	
4/10/2011	2	NF	1	Stylogomphus albistylus	Gomphidae	Odonata	3	
4/10/2011	2	NF	1	Psephenus herricki	Psephenidae	Coleoptera	1	L/R
4/10/2011	2	NF	1	Thienemanniella sp.	Orthocladiinae	Diptera	2	
4/10/2011	2	NF	1	Polypedilum (sp. A)	Chironominae	Diptera	1	
4/10/2011	2	NF	1	Psephenus herricki	Psephenidae	Coleoptera	2	
4/10/2011	2	NF	2	Ablabesmyia sp.	Tanypodinae	Diptera	28	
4/10/2011	2	NF	2	Acarina sp.	Hydracarina	Arachnoidea	15	

S1 Appendix. Raw benthic macroinvertebrate data from Crane Pond Creek, Iron County, Missouri, USA.

4/10/2011	2	NF	2	Haploperla brevius	Chloroperlidae	Plecoptera	1	L/R
4/10/2011	2	NF	2	Haploperla brevius	Chloroperlidae	Plecoptera	2	
4/10/2011	2	NF	2	Cricotopus bicinctus	Orthocladiinae	Diptera	11	
4/10/2011	2	NF	2	Polypedilum (sp. A)	Chironominae	Diptera	1	
4/10/2011	2	NF	2	Psephenus herricki	Psephenidae	Coleoptera	8	
4/10/2011	2	NF	2	Hagenius brevistylus	Gomphidae	Odonata	1	L/R
4/10/2011	2	NF	2	Dicrotendipes sp.	Chironominae	Diptera	14	
4/10/2011	2	NF	2	Fallceon sp.	Baetidae	Ephemeroptera	1	
4/10/2011	2	NF	2	Ectopria nervosa	Psephenidae	Coleoptera	1	
4/10/2011	2	NF	2	Eurylophella sp.	Ephemerellidae	Ephemeroptera	16	
4/10/2011	2	NF	2	Paratendipes sp.	Chironominae	Diptera	1	
4/10/2011	2	NF	2	Thienemanniella sp.	Orthocladiinae	Diptera	1	
4/10/2011	2	NF	2	Polypedilum illinoense	Chironominae	Diptera	3	
4/10/2011	2	NF	2	Psephenus herricki	Psephenidae	Coleoptera	2	L/R
4/10/2011	2	NF	2	Orconectes peruncus	Cambaridae	Decapoda	1	L/R
4/10/2011	2	NF	2	Polypedilum fallax	Chironominae	Diptera	1	
4/10/2011	2	NF	2	Nilotanypus sp.	Tanypodinae	Diptera	1	
4/10/2011	2	NF	2	Orthocladius sp. (yellow dome tooth)	Orthocladiinae	Diptera	43	
4/10/2011	2	NF	2	Stenochironomus sp.	Chironominae	Diptera	12	
4/10/2011	2	NF	2	Paratanytarsus sp.	Chironominae	Diptera	7	
4/10/2011	2	NF	2	Chironomidae	Chironomidae	Diptera	307	
4/10/2011	2	NF	2	Cladotanytarsus sp.	Chironominae	Diptera	55	
4/10/2011	2	NF	2	Tanytarsus sp.	Chironominae	Diptera	55	
4/10/2011	2	NF	2	Cryptochironomus sp.	Chironominae	Diptera	4	
4/10/2011	2	NF	2	Microtendipes sp.	Chironominae	Diptera	13	
4/10/2011	2	NF	2	Amphinemura sp.	Nemouridae	Plecoptera	1	
4/10/2011	2	NF	2	Polypedilum convictum	Chironominae	Diptera	1	
4/10/2011	2	NF	2	Stenelmis sp.	Elmidae	Coleoptera	1	L/R
4/10/2011	2	NF	2	Stenelmis sp.	Elmidae	Coleoptera	25	
4/10/2011	2	NF	2	Rheocricotopus sp.	Orthocladiinae	Diptera	2	
4/10/2011	2	NF	2	Parakiefferiella sp.	Orthocladiinae	Diptera	1	
4/10/2011	2	NF	2	Chironomus sp.	Chironominae	Diptera	1	
4/10/2011	2	NF	2	Stylogomphus albistylus	Gomphidae	Odonata	1	
4/10/2011	2	NF	2	Helicopsyche sp.	Helicopsychidae	Tricoptera	9	
4/10/2011	2	NF	2	Cricotopus isocladius	Orthocladiinae	Diptera	12	
4/10/2011	2	NF	2	Stenonema femoratum	Heptageniidae	Ephemeroptera	16	
4/10/2011	2	NF	2	Stenonema femoratum	Heptageniidae	Ephemeroptera	4	L/R

S1 Appendix. Raw benthic macroinvertebrate data from Crane Pond Creek, Iron County, Missouri, USA.

4/10/2011	2	NF	2	Stylogomphus albistylus	Gomphidae	Odonata	3	L/R
4/10/2011	2	NF	2	Tricorythodes sp.	Leptohyphidae	Ephemeroptera	1	
4/10/2011	2	NF	2	Phaenopsectra sp.	Chironominae	Diptera	26	
4/10/2011	2	NF	2	Lauterborniella sp.	Chironominae	Diptera	31	
4/10/2011	2	NF	2	Eukiefferiella sp.	Orthocladiinae	Diptera	1	
4/10/2011	2	NF	2	Thienemannimyia grp	Tanypodinae	Diptera	13	
4/10/2011	2	NF	2	Argia sp.	Coengrionidae	Odonata	7	
4/10/2011	2	NF	3	Polypedilum convictum	Chironominae	Diptera	1	
4/10/2011	2	NF	3	Polypedilum fallax	Chironominae	Diptera	1	
4/10/2011	2	NF	3	Thienemanniella sp.	Orthocladiinae	Diptera	3	
4/10/2011	2	NF	3	Polypedilum illinoense	Chironominae	Diptera	1	
4/10/2011	2	NF	3	Hemerodromia sp.	Empididae	Diptera	1	
4/10/2011	2	NF	3	Parakiefferiella sp.	Orthocladiinae	Diptera	8	
4/10/2011	2	NF	3	Eurylophella sp.	Ephemerellidae	Ephemeroptera	1	
4/10/2011	2	NF	3	Chironomidae	Chironomidae	Diptera	290	
4/10/2011	2	NF	3	Rheocricotopus sp.	Orthocladiinae	Diptera	3	
4/10/2011	2	NF	3	Baetidae	Baetidae	Ephemeroptera	1	L/R
4/10/2011	2	NF	3	Paratanytarsus sp.	Chironominae	Diptera	6	
4/10/2011	2	NF	3	Stenelmis sp.	Elmidae	Coleoptera	1	L/R
4/10/2011	2	NF	3	Baetidae	Baetidae	Ephemeroptera	1	
4/10/2011	2	NF	3	Haploperla brevius	Chloroperlidae	Plecoptera	2	
4/10/2011	2	NF	3	Bezzia sp.	Ceratopogonidae	Diptera	1	
4/10/2011	2	NF	3	Baetisca sp.	Baetiscidae	Ephemeroptera	1	L/R
4/10/2011	2	NF	3	Stenelmis sp.	Elmidae	Coleoptera	10	
4/10/2011	2	NF	3	Helicopsyche sp.	Helicopsychidae	Tricoptera	2	
4/10/2011	2	NF	3	Orthocladius sp. (yellow dome tooth)	Orthocladiinae	Diptera	36	
4/10/2011	2	NF	3	Cryptochironomus sp.	Chironominae	Diptera	2	
4/10/2011	2	NF	3	Dubiraphia sp.	Elmidae	Coleoptera	1	Larvae
4/10/2011	2	NF	3	Dicrotendipes sp.	Chironominae	Diptera	3	
4/10/2011	2	NF	3	Ochrotrichia sp.	Hydroptilidae	Tricoptera	1	
4/10/2011	2	NF	3	Ablabesmyia sp.	Tanypodinae	Diptera	22	
4/10/2011	2	NF	3	Cricotopus isocladius	Orthocladiinae	Diptera	7	
4/10/2011	2	NF	3	Psephenus herricki	Psephenidae	Coleoptera	4	L/R
4/10/2011	2	NF	3	Acarina sp.	Hydracarina	Arachnoidea	8	
4/10/2011	2	NF	3	Gyraulus sp.	Planorbidae	Gastropoda	1	
4/10/2011	2	NF	3	Ectopria nervosa	Psephenidae	Coleoptera	1	
4/10/2011	2	NF	3	Stenonema femoratum	Heptageniidae	Ephemeroptera	9	

S1 Appendix. Raw benthic macroinvertebrate data from Crane Pond Creek, Iron County, Missouri, USA.

4/10/2011	2	NF	3	Tricorythodes sp.	Leptohyphidae	Ephemeroptera	15	
4/10/2011	2	NF	3	Argia sp.	Coengrionidae	Odonata	2	
4/10/2011	2	NF	3	Tabanus sp.	Tabanidae	Diptera	1	L/R
4/10/2011	2	NF	3	Eukiefferiella sp.	Orthocladiinae	Diptera	2	
4/10/2011	2	NF	3	Phaenopsectra sp.	Chironominae	Diptera	34	
4/10/2011	2	NF	3	Nilotanypus sp.	Tanypodinae	Diptera	3	
4/10/2011	2	NF	3	Tanytarsus sp.	Chironominae	Diptera	35	
4/10/2011	2	NF	3	Stenonema femoratum	Heptageniidae	Ephemeroptera	10	L/R
4/10/2011	2	NF	3	Microtendipes sp.	Chironominae	Diptera	4	
4/10/2011	2	NF	3	Chimarra sp.	Philopotamidae	Tricoptera	3	
4/10/2011	2	NF	3	Lauterborniella sp.	Chironominae	Diptera	17	
4/10/2011	2	NF	3	Thienemannimyia grp	Tanypodinae	Diptera	4	
4/10/2011	2	NF	3	Cricotopus bicinctus	Orthocladiinae	Diptera	13	
4/10/2011	2	NF	3	Cladotanytarsus sp.	Chironominae	Diptera	55	
6/24/2011	1	CS	1	Acarina sp.	Hydracarina	Arachnoidea	14	
6/24/2011	1	CS	1	Dashyheleinae sp.	Ceratopogonidae	Diptera	1	
6/24/2011	1	CS	1	Maccaffertium sp.	Heptageniidae	Ephemeroptera	27	
6/24/2011	1	CS	1	Orconectes hylas	Cambaridae	Decapoda	5	L/R
6/24/2011	1	CS	1	Psephenus herricki	Psephenidae	Coleoptera	4	
6/24/2011	1	CS	1	Elimia potosensis	Pleuroceridae	Gastropoda	2	L/R
6/24/2011	1	CS	1	Rheotanytarsus sp.	Chironominae	Diptera	3	
6/24/2011	1	CS	1	Chironomidae Pupae	Chironomidae	Diptera	2	
6/24/2011	1	CS	1	Optioservus sp.	Elmidae	Coleoptera	4	
6/24/2011	1	CS	1	Bezzia sp.	Ceratopogonidae	Diptera	1	
6/24/2011	1	CS	1	Hydropsyche sp. Pupa	Hydropsychidae	Tricoptera	1	
6/24/2011	1	CS	1	Elimia potosensis	Pleuroceridae	Gastropoda	1	
6/24/2011	1	CS	1	Tanytarsus sp.	Chironominae	Diptera	63	
6/24/2011	1	CS	1	Nigronia sp.	Corydalidae	Megaloptera	2	
6/24/2011	1	CS	1	Tricorythodes sp.	Leptohyphidae	Ephemeroptera	22	
6/24/2011	1	CS	1	Optioservus sandersoni	Elmidae	Coleoptera	15	
6/24/2011	1	CS	1	Thienemanniella sp.	Orthocladiinae	Diptera	2	
6/24/2011	1	CS	1	Fallceon sp.	Baetidae	Ephemeroptera	35	
6/24/2011	1	CS	1	Stenelmis lateralis	Elmidae	Coleoptera	1	L/R
6/24/2011	1	CS	1	Helicopsyche sp.	Helicopsychidae	Tricoptera	1	L/R
6/24/2011	1	CS	1	Tabanus sp.	Tabanidae	Diptera	1	L/R
6/24/2011	1	CS	1	Stenelmis sp.	Elmidae	Coleoptera	116	
6/24/2011	1	CS	1	Tabanus sp.	Tabanidae	Diptera	1	

S1 Appendix. Raw benthic macroinvertebrate data from Crane Pond Creek, Iron County, Missouri, USA.

6/24/2011	1	CS	1	Thienemannimyia grp	Tanypodinae	Diptera	1	
6/24/2011	1	CS	1	Orconectes hylas	Cambaridae	Decapoda	5	
6/24/2011	1	CS	1	Isonychia bicolor	Isonychiidae	Ephemeroptera	3	L/R
6/24/2011	1	CS	1	Corydalis sp.	Corydalidae	Megaloptera	3	L/R
6/24/2011	1	CS	1	Chimarra sp.	Philopotamidae	Tricoptera	11	
6/24/2011	1	CS	1	Corydalis sp.	Corydalidae	Megaloptera	1	
6/24/2011	1	CS	1	Neoperla sp.	Perlidae	Plecoptera	8	
6/24/2011	1	CS	1	Marilia sp.	Odontoceridae	Tricoptera	1	
6/24/2011	1	CS	1	Hemerodromia sp.	Empididae	Diptera	1	
6/24/2011	1	CS	1	Neoperla harpi	Perlidae	Plecoptera	5	L/R
6/24/2011	1	CS	1	Simulium sp.	Simuliidae	Diptera	6	
6/24/2011	1	CS	1	Neoperla osage	Perlidae	Plecoptera	6	L/R
6/24/2011	1	CS	1	Diptera Pupa		Diptera	1	
6/24/2011	1	CS	1	Neoperla robisoni	Perlidae	Plecoptera	1	L/R
6/24/2011	1	CS	1	Maccaffertium mediopunctatum	Heptageniidae	Ephemeroptera	3	L/R
6/24/2011	1	CS	1	Cheumatopsyche sp.	Hydropsychidae	Tricoptera	148	
6/24/2011	1	CS	1	Atherix sp.	Athericidae	Diptera	1	L/R
6/24/2011	1	CS	1	Stenelmis lateralis	Elmidae	Coleoptera	55	
6/24/2011	1	CS	1	Maccaffertium mediopunctatum	Heptageniidae	Ephemeroptera	22	
6/24/2011	1	CS	1	Psephenus herricki	Psephenidae	Coleoptera	2	L/R
6/24/2011	1	CS	1	Cricotopus bicinctus	Orthocladiinae	Diptera	6	
6/24/2011	1	CS	1	Rheocricotopus sp.	Orthocladiinae	Diptera	8	
6/24/2011	1	CS	1	Orthocladius sp. (yellow dome tooth)	Orthocladiinae	Diptera	3	
6/24/2011	1	CS	1	Tubificidae	Tubificidae	Tubificida	1	
6/24/2011	1	CS	1	Polypedilum (sp. A)	Chironominae	Diptera	8	
6/24/2011	1	CS	1	Polypedilum convictum	Chironominae	Diptera	26	
6/24/2011	1	CS	1	Chironomidae	Chironomidae	Diptera	108	
6/24/2011	1	CS	2	Chironomidae Pupae	Chironomidae	Diptera	10	
6/24/2011	1	CS	2	Dashyheleinae sp.	Ceratopogonidae	Diptera	1	
6/24/2011	1	CS	2	Elimia potosensis	Pleuroceridae	Gastropoda	3	L/R
6/24/2011	1	CS	2	Elimia potosensis	Pleuroceridae	Gastropoda	3	
6/24/2011	1	CS	2	Optioservus sandersoni	Elmidae	Coleoptera	11	
6/24/2011	1	CS	2	Thienemanniella sp.	Orthocladiinae	Diptera	3	
6/24/2011	1	CS	2	Stenelmis sp.	Elmidae	Coleoptera	235	
6/24/2011	1	CS	2	Cricotopus bicinctus	Orthocladiinae	Diptera	5	
6/24/2011	1	CS	2	Cheumatopsyche sp.	Hydropsychidae	Tricoptera	18	
6/24/2011	1	CS	2	Bezzia sp.	Ceratopogonidae	Diptera	1	

S1 Appendix. Raw benthic macroinvertebrate data from Crane Pond Creek, Iron County, Missouri, USA.

6/24/2011	1	CS	2	Cladotanytarsus sp.	Chironominae	Diptera	1	
6/24/2011	1	CS	2	Maccaffertium sp.	Heptageniidae	Ephemeroptera	28	
6/24/2011	1	CS	2	Tricorythodes sp.	Leptohyphidae	Ephemeroptera	31	
6/24/2011	1	CS	2	Tanytarsus sp.	Chironominae	Diptera	53	
6/24/2011	1	CS	2	Neoperla harpi	Perlidae	Plecoptera	2	
6/24/2011	1	CS	2	Psephenus herricki	Psephenidae	Coleoptera	6	
6/24/2011	1	CS	2	Chimarra sp.	Philopotamidae	Tricoptera	4	
6/24/2011	1	CS	2	Dashyheleinae sp.	Ceratopogonidae	Diptera	2	
6/24/2011	1	CS	2	Argia sp.	Coengrionidae	Odonata	1	L/R
6/24/2011	1	CS	2	Eukiefferiella sp.	Orthocladiinae	Diptera	2	
6/24/2011	1	CS	2	Polypedilum (sp. A)	Chironominae	Diptera	2	
6/24/2011	1	CS	2	Chironomidae	Chironomidae	Diptera	75	
6/24/2011	1	CS	2	Orthocladius sp. (yellow dome tooth)	Orthocladiinae	Diptera	1	
6/24/2011	1	CS	2	Thienemannimyia grp	Tanypodinae	Diptera	2	
6/24/2011	1	CS	2	Orconectes hylas	Cambaridae	Decapoda	4	L/R
6/24/2011	1	CS	2	Orconectes hylas	Cambaridae	Decapoda	1	
6/24/2011	1	CS	2	Neoperla harpi	Perlidae	Plecoptera	1	L/R
6/24/2011	1	CS	2	Rheocricotopus sp.	Orthocladiinae	Diptera	1	
6/24/2011	1	CS	2	Corydalis sp.	Corydalidae	Megaloptera	4	L/R
6/24/2011	1	CS	2	Fallceon sp.	Baetidae	Ephemeroptera	33	
6/24/2011	1	CS	2	Isonychia bicolor	Isonychiidae	Ephemeroptera	8	
6/24/2011	1	CS	2	Corydalis sp.	Corydalidae	Megaloptera	1	
6/24/2011	1	CS	2	Stenelmis lateralis	Elmidae	Coleoptera	100	
6/24/2011	1	CS	2	Amphinemura sp.	Nemouridae	Plecoptera	3	
6/24/2011	1	CS	2	Stenelmis lateralis	Elmidae	Coleoptera	1	L/R
6/24/2011	1	CS	2	Hydropsyche sp.	Hydropsychidae	Tricoptera	7	
6/24/2011	1	CS	2	Maccaffertium mediopunctatum	Heptageniidae	Ephemeroptera	15	
6/24/2011	1	CS	2	Haploperla brevius	Chloroperlidae	Plecoptera	1	
6/24/2011	1	CS	2	Acarina sp.	Hydracarina	Arachnoidea	9	
6/24/2011	1	CS	2	Stenelmis sp.	Elmidae	Coleoptera	1	L/R
6/24/2011	1	CS	2	Tubificidae	Tubificidae	Tubificida	1	
6/24/2011	1	CS	2	Tabanus sp.	Tabanidae	Diptera	1	L/R
6/24/2011	1	CS	2	Atherix sp.	Athericidae	Diptera	2	L/R
6/24/2011	1	CS	2	Rheotanytarsus sp.	Chironominae	Diptera	1	
6/24/2011	1	CS	2	Cheumatopsyche sp.	Hydropsychidae	Tricoptera	136	
6/24/2011	1	CS	2	Lumbriculidae	Lumbriculidae	Lumbriculida	1	
6/24/2011	1	CS	2	Neoperla osage	Perlidae	Plecoptera	5	

S1 Appendix. Raw benthic macroinvertebrate data from Crane Pond Creek, Iron County, Missouri, USA.

6/24/2011	1	CS	2	Polypedilum convictum	Chironominae	Diptera	19	
6/24/2011	1	CS	2	Heptageniidae	Heptageniidae	Ephemeroptera	1	L/R
6/24/2011	1	CS	2	Hydropsyche sp.	Hydropsychidae	Tricoptera	2	L/R
6/24/2011	1	CS	2	Neoperla osage	Perlidae	Plecoptera	6	L/R
6/24/2011	1	CS	3	Cricotopus bicinctus	Orthocladiinae	Diptera	3	
6/24/2011	1	CS	3	Elimia potosensis	Pleuroceridae	Gastropoda	4	L/R
6/24/2011	1	CS	3	Lumbriculidae	Lumbriculidae	Lumbriculida	2	
6/24/2011	1	CS	3	Bezzia sp.	Ceratopogonidae	Diptera	2	
6/24/2011	1	CS	3	Atherix sp.	Athericidae	Diptera	2	
6/24/2011	1	CS	3	Tabanus sp.	Tabanidae	Diptera	1	
6/24/2011	1	CS	3	Cheumatopsyche sp.	Hydropsychidae	Tricoptera	74	
6/24/2011	1	CS	3	Thienemanniella sp.	Orthocladiinae	Diptera	3	
6/24/2011	1	CS	3	Simulium sp.	Simuliidae	Diptera	10	
6/24/2011	1	CS	3	Optioservus sandersoni	Elmidae	Coleoptera	28	
6/24/2011	1	CS	3	Isonychia bicolor	Isonychiidae	Ephemeroptera	2	
6/24/2011	1	CS	3	Fallceon sp.	Baetidae	Ephemeroptera	63	
6/24/2011	1	CS	3	Stenelmis lateralis	Elmidae	Coleoptera	43	
6/24/2011	1	CS	3	Tricorythodes sp.	Leptohyphidae	Ephemeroptera	15	
6/24/2011	1	CS	3	Corynoneura sp.	Orthocladiinae	Diptera	1	
6/24/2011	1	CS	3	Maccaffertium mediopunctatum	Heptageniidae	Ephemeroptera	32	
6/24/2011	1	CS	3	Psephenus herricki	Psephenidae	Coleoptera	1	
6/24/2011	1	CS	3	Glossiphoniidae	Rhynchobdellida	Hirudinea	1	
6/24/2011	1	CS	3	Neoperla osage	Perlidae	Plecoptera	2	L/R
6/24/2011	1	CS	3	Maccaffertium mediopunctatum	Heptageniidae	Ephemeroptera	3	L/R
6/24/2011	1	CS	3	Hydropsychidae Pupa	Hydropsychidae	Tricoptera	1	L/R
6/24/2011	1	CS	3	Baetidae	Baetidae	Ephemeroptera	2	L/R
6/24/2011	1	CS	3	Eukiefferiella sp.	Orthocladiinae	Diptera	4	
6/24/2011	1	CS	3	Atherix sp.	Athericidae	Diptera	2	L/R
6/24/2011	1	CS	3	Stenelmis sp.	Elmidae	Coleoptera	2	L/R
6/24/2011	1	CS	3	Neoperla harpi	Perlidae	Plecoptera	4	L/R
6/24/2011	1	CS	3	Isonychia bicolor	Isonychiidae	Ephemeroptera	1	L/R
6/24/2011	1	CS	3	Tabanus sp.	Tabanidae	Diptera	3	L/R
6/24/2011	1	CS	3	Baetidae	Baetidae	Ephemeroptera	21	
6/24/2011	1	CS	3	Chironomidae	Chironomidae	Diptera	61	
6/24/2011	1	CS	3	Hydropsyche sp.	Hydropsychidae	Tricoptera	8	
6/24/2011	1	CS	3	Chironomidae Pupae	Chironomidae	Diptera	6	
6/24/2011	1	CS	3	Cheumatopsyche sp.	Hydropsychidae	Tricoptera	14	

S1 Appendix. Raw benthic macroinvertebrate data from Crane Pond Creek, Iron County, Missouri, USA.

6/24/2011	1	CS	3	Cheumatopsyche sp.	Hydropsychidae	Tricoptera	1	L/R
6/24/2011	1	CS	3	Corydalis sp.	Corydalidae	Megaloptera	1	L/R
6/24/2011	1	CS	3	Hemerodromia sp.	Empididae	Diptera	2	
6/24/2011	1	CS	3	Polypedilum illinoense	Chironominae	Diptera	2	
6/24/2011	1	CS	3	Rheotanytarsus sp.	Chironominae	Diptera	6	
6/24/2011	1	CS	3	Neoperla harpi	Perlidae	Plecoptera	6	
6/24/2011	1	CS	3	Helicopsyche sp.	Helicopsychidae	Tricoptera	2	
6/24/2011	1	CS	3	Hexatoma sp.	Tipulidae	Diptera	2	
6/24/2011	1	CS	3	Orconectes hylas	Cambaridae	Decapoda	8	L/R
6/24/2011	1	CS	3	Acarina sp.	Hydracarina	Arachnoidea	22	
6/24/2011	1	CS	3	Polypedilum (sp. A)	Chironominae	Diptera	10	
6/24/2011	1	CS	3	Psephenus herricki	Psephenidae	Coleoptera	1	L/R
6/24/2011	1	CS	3	Optioservus sp.	Elmidae	Coleoptera	6	
6/24/2011	1	CS	3	Stenelmis sp.	Elmidae	Coleoptera	196	
6/24/2011	1	CS	3	Neoperla osage	Perlidae	Plecoptera	2	
6/24/2011	1	CS	3	Hydropsyche sp. Pupa	Hydropsychidae	Tricoptera	1	
6/24/2011	1	CS	3	Perlidae	Perlidae	Plecoptera	1	
6/24/2011	1	CS	3	Polypedilum convictum	Chironominae	Diptera	11	
6/24/2011	1	CS	3	Nigronia sp.	Corydalidae	Megaloptera	3	
6/24/2011	1	CS	3	Simulium sp. Pupa	Simuliidae	Diptera	6	
6/24/2011	1	CS	3	Tanytarsus sp.	Chironominae	Diptera	11	
6/24/2011	1	CS	3	Maccaffertium sp.	Heptageniidae	Ephemeroptera	7	
6/24/2011	1	NF	1	Hagenius brevistylus	Gomphidae	Odonata	1	L/R
6/24/2011	1	NF	1	Dashyheleinae sp.	Ceratopogonidae	Diptera	6	
6/24/2011	1	NF	1	Paratanytarsus sp.	Chironominae	Diptera	4	
6/24/2011	1	NF	1	Polypedilum convictum	Chironominae	Diptera	3	
6/24/2011	1	NF	1	Neoperla osage	Perlidae	Plecoptera	1	L/R
6/24/2011	1	NF	1	Stylogomphus albistylus	Gomphidae	Odonata	2	
6/24/2011	1	NF	1	Baetidae	Baetidae	Ephemeroptera	5	
6/24/2011	1	NF	1	Neoperla sp.	Perlidae	Plecoptera	3	
6/24/2011	1	NF	1	Neoperla osage	Perlidae	Plecoptera	1	
6/24/2011	1	NF	1	Lauterborniella sp.	Chironominae	Diptera	1	
6/24/2011	1	NF	1	Stenonema femoratum	Heptageniidae	Ephemeroptera	8	
6/24/2011	1	NF	1	Chironomidae Pupae	Chironomidae	Diptera	2	
6/24/2011	1	NF	1	Dubiraphia sp.	Elmidae	Coleoptera	7	Larvae
6/24/2011	1	NF	1	Microtendipes sp.	Chironominae	Diptera	1	
6/24/2011	1	NF	1	Tanytarsus sp.	Chironominae	Diptera	53	

S1 Appendix. Raw benthic macroinvertebrate data from Crane Pond Creek, Iron County, Missouri, USA.

6/24/2011	1	NF	1	Dicrotendipes sp.	Chironominae	Diptera	28	
6/24/2011	1	NF	1	Cryptochironomus sp.	Chironominae	Diptera	6	
6/24/2011	1	NF	1	Chironomidae	Chironomidae	Diptera	228	
6/24/2011	1	NF	1	Acarina sp.	Hydracarina	Arachnoidea	15	
6/24/2011	1	NF	1	Stenelmis sp.	Elmidae	Coleoptera	44	
6/24/2011	1	NF	1	Cryptotendipes sp.	Chironominae	Diptera	1	
6/24/2011	1	NF	1	Stenelmis lateralis	Elmidae	Coleoptera	14	
6/24/2011	1	NF	1	Psephenus herricki	Psephenidae	Coleoptera	1	
6/24/2011	1	NF	1	Orthocladius sp. (yellow dome tooth)	Orthocladiinae	Diptera	23	
6/24/2011	1	NF	1	Tricorythodes sp.	Leptohyphidae	Ephemeroptera	1	
6/24/2011	1	NF	1	Phaenopsectra sp.	Chironominae	Diptera	40	
6/24/2011	1	NF	1	Polypedilum (sp. A)	Chironominae	Diptera	1	
6/24/2011	1	NF	1	Ablabesmyia sp.	Tanypodinae	Diptera	6	
6/24/2011	1	NF	1	Stenonema femoratum	Heptageniidae	Ephemeroptera	3	L/R
6/24/2011	1	NF	1	Thienemanniella sp.	Orthocladiinae	Diptera	1	
6/24/2011	1	NF	1	Cricotopus bicinctus	Orthocladiinae	Diptera	11	
6/24/2011	1	NF	1	Orconectes sp.	Cambaridae	Decapoda	1	L/R
6/24/2011	1	NF	1	Orconectes luteus	Cambaridae	Decapoda	1	L/R
6/24/2011	1	NF	1	Lumbriculidae	Lumbriculidae	Lumbriculida	1	
6/24/2011	1	NF	1	Paralauterborniella sp.	Chironominae	Diptera	1	
6/24/2011	1	NF	1	Parakiefferiella sp.	Orthocladiinae	Diptera	19	
6/24/2011	1	NF	1	Argia sp.	Coengrionidae	Odonata	1	L/R
6/24/2011	1	NF	1	Fallceon sp.	Baetidae	Ephemeroptera	1	
6/24/2011	1	NF	1	Caenis sp.	Caenidae	Ephemeroptera	1	
6/24/2011	1	NF	1	Thienemannimyia grp	Tanypodinae	Diptera	6	
6/24/2011	1	NF	1	Neoperla harpi	Perlidae	Plecoptera	1	
6/24/2011	1	NF	1	Cladotanytarsus sp.	Chironominae	Diptera	26	
6/24/2011	1	NF	2	Orthocladius sp. (yellow dome tooth)	Orthocladiinae	Diptera	1	
6/24/2011	1	NF	2	Orconectes sp.	Cambaridae	Decapoda	1	
6/24/2011	1	NF	2	Cladotanytarsus sp.	Chironominae	Diptera	5	
6/24/2011	1	NF	2	Thienemannimyia grp	Tanypodinae	Diptera	5	
6/24/2011	1	NF	2	Argia sp.	Coengrionidae	Odonata	1	L/R
6/24/2011	1	NF	2	Baetidae	Baetidae	Ephemeroptera	5	
6/24/2011	1	NF	2	Polypedilum (sp. A)	Chironominae	Diptera	5	
6/24/2011	1	NF	2	Chironomidae Pupae	Chironomidae	Diptera	20	
6/24/2011	1	NF	2	Helicopsyche sp.	Helicopsychidae	Tricoptera	1	
6/24/2011	1	NF	2	Phaenopsectra sp.	Chironominae	Diptera	5	

S1 Appendix. Raw benthic macroinvertebrate data from Crane Pond Creek, Iron County, Missouri, USA.

6/24/2011	1	NF	2	Argia sp.	Coengrionidae	Odonata	1	
6/24/2011	1	NF	2	Dicrotendipes sp.	Chironominae	Diptera	7	
6/24/2011	1	NF	2	Optioservus sp.	Elmidae	Coleoptera	1	
6/24/2011	1	NF	2	Polypedilum convictum	Chironominae	Diptera	9	
6/24/2011	1	NF	2	Neoperla osage	Perlidae	Plecoptera	12	
6/24/2011	1	NF	2	Stenelmis lateralis	Elmidae	Coleoptera	22	
6/24/2011	1	NF	2	Dashyheleinae sp.	Ceratopogonidae	Diptera	1	
6/24/2011	1	NF	2	Cheumatopsyche sp.	Hydropsychidae	Tricoptera	8	
6/24/2011	1	NF	2	Psephenus herricki	Psephenidae	Coleoptera	3	
6/24/2011	1	NF	2	Stylogomphus albistylus	Gomphidae	Odonata	1	
6/24/2011	1	NF	2	Stenonema femoratum	Heptageniidae	Ephemeroptera	42	
6/24/2011	1	NF	2	Chironomidae	Chironomidae	Diptera	178	
6/24/2011	1	NF	2	Cricotopus bicinctus	Orthocladiinae	Diptera	2	
6/24/2011	1	NF	2	Orconectes sp.	Cambaridae	Decapoda	2	L/R
6/24/2011	1	NF	2	Paratanytarsus sp.	Chironominae	Diptera	1	
6/24/2011	1	NF	2	Chimarra sp.	Philopotamidae	Tricoptera	27	
6/24/2011	1	NF	2	Tricorythodes sp.	Leptohyphidae	Ephemeroptera	11	
6/24/2011	1	NF	2	Stenelmis sp.	Elmidae	Coleoptera	58	
6/24/2011	1	NF	2	Lauterborniella sp.	Chironominae	Diptera	6	
6/24/2011	1	NF	2	Tanytarsus sp.	Chironominae	Diptera	94	
6/24/2011	1	NF	2	Stenonema femoratum	Heptageniidae	Ephemeroptera	1	L/R
6/24/2011	1	NF	2	Stylogomphus albistylus	Gomphidae	Odonata	1	L/R
6/24/2011	1	NF	2	Ochrotrichia sp.	Hydroptilidae	Tricoptera	2	
6/24/2011	1	NF	2	Ablabesmyia sp.	Tanypodinae	Diptera	16	
6/24/2011	1	NF	2	Acarina sp.	Hydracarina	Arachnoidea	13	
6/24/2011	1	NF	2	Microtendipes sp.	Chironominae	Diptera	3	
6/24/2011	1	NF	3	Lauterborniella sp.	Chironominae	Diptera	5	
6/24/2011	1	NF	3	Microtendipes sp.	Chironominae	Diptera	2	
6/24/2011	1	NF	3	Thienemannimyia grp	Tanypodinae	Diptera	2	
6/24/2011	1	NF	3	Stenelmis sp.	Elmidae	Coleoptera	87	
6/24/2011	1	NF	3	Baetidae	Baetidae	Ephemeroptera	4	
6/24/2011	1	NF	3	Stylogomphus albistylus	Gomphidae	Odonata	1	
6/24/2011	1	NF	3	Tricorythodes sp.	Leptohyphidae	Ephemeroptera	6	
6/24/2011	1	NF	3	Caenis sp.	Caenidae	Ephemeroptera	1	
6/24/2011	1	NF	3	Ochrotrichia sp.	Hydroptilidae	Tricoptera	2	
6/24/2011	1	NF	3	Polypedilum (sp. A)	Chironominae	Diptera	2	
6/24/2011	1	NF	3	Psephenus herricki	Psephenidae	Coleoptera	2	

S1 Appendix. Raw benthic macroinvertebrate data from Crane Pond Creek, Iron County, Missouri, USA.

6/24/2011	1	NF	3	Neoperla sp.	Perlidae	Plecoptera	2	L/R
6/24/2011	1	NF	3	Cheumatopsyche sp.	Hydropsychidae	Tricoptera	8	
6/24/2011	1	NF	3	Chimarra sp.	Philopotamidae	Tricoptera	12	
6/24/2011	1	NF	3	Acarina sp.	Hydracarina	Arachnoidea	9	
6/24/2011	1	NF	3	Cladotanytarsus sp.	Chironominae	Diptera	4	
6/24/2011	1	NF	3	Helicopsyche sp.	Helicopsychidae	Tricoptera	2	
6/24/2011	1	NF	3	Orconectes sp.	Cambaridae	Decapoda	1	
6/24/2011	1	NF	3	Tanytarsus sp.	Chironominae	Diptera	66	
6/24/2011	1	NF	3	Ablabesmyia sp.	Tanypodinae	Diptera	17	
6/24/2011	1	NF	3	Polypedilum convictum	Chironominae	Diptera	9	
6/24/2011	1	NF	3	Rheocricotopus sp.	Orthocladiinae	Diptera	1	
6/24/2011	1	NF	3	Stenonema femoratum	Heptageniidae	Ephemeroptera	35	
6/24/2011	1	NF	3	Cricotopus bicinctus	Orthocladiinae	Diptera	1	
6/24/2011	1	NF	3	Phaenopsectra sp.	Chironominae	Diptera	1	
6/24/2011	1	NF	3	Chironomidae Pupae	Chironomidae	Diptera	19	
6/24/2011	1	NF	3	Orthocladius sp. (yellow dome tooth)	Orthocladiinae	Diptera	1	
6/24/2011	1	NF	3	Cryptochironomus sp.	Chironominae	Diptera	1	
6/24/2011	1	NF	3	Ectopria nervosa	Psephenidae	Coleoptera	1	
6/24/2011	1	NF	3	Stenelmis lateralis	Elmidae	Coleoptera	15	
6/24/2011	1	NF	3	Thienemanniella sp.	Orthocladiinae	Diptera	3	
6/24/2011	1	NF	3	Chironomidae	Chironomidae	Diptera	159	
6/24/2011	1	NF	3	Stenonema femoratum	Heptageniidae	Ephemeroptera	1	L/R
6/24/2011	1	NF	3	Neoperla osage	Perlidae	Plecoptera	1	L/R
6/24/2011	1	NF	3	Tricorythodes sp.	Leptohyphidae	Ephemeroptera	1	L/R
6/24/2011	1	NF	3	Orconectes sp.	Cambaridae	Decapoda	2	L/R
6/24/2011	1	NF	3	Neoperla sp.	Perlidae	Plecoptera	15	
6/24/2011	1	NF	3	Dicrotendipes sp.	Chironominae	Diptera	10	
6/24/2011	2	CS	1	Stenelmis lateralis	Elmidae	Coleoptera	21	
6/24/2011	2	CS	1	Chimarra sp.	Philopotamidae	Tricoptera	1	L/R
6/24/2011	2	CS	1	Orconectes luteus	Cambaridae	Decapoda	3	L/R
6/24/2011	2	CS	1	Chironomidae	Chironomidae	Diptera	204	
6/24/2011	2	CS	1	Caenis sp.	Caenidae	Ephemeroptera	10	
6/24/2011	2	CS	1	Psephenus herricki	Psephenidae	Coleoptera	2	L/R
6/24/2011	2	CS	1	Tanytarsus sp.	Chironominae	Diptera	114	
6/24/2011	2	CS	1	Oecetis sp.	Leptoceridae	Tricoptera	4	
6/24/2011	2	CS	1	Hydropsyche sp.	Hydropsychidae	Tricoptera	2	
6/24/2011	2	CS	1	Psephenus herricki	Psephenidae	Coleoptera	9	

S1 Appendix. Raw benthic macroinvertebrate data from Crane Pond Creek, Iron County, Missouri, USA.

6/24/2011	2	CS	1	Hydropsychidae Pupa	Hydropsychidae	Tricoptera	1	
6/24/2011	2	CS	1	Helicopsyche sp.	Helicopsychidae	Tricoptera	9	
6/24/2011	2	CS	1	Polypedilum (sp. A)	Chironominae	Diptera	18	
6/24/2011	2	CS	1	Cricotopus bicinctus	Orthocladiinae	Diptera	2	
6/24/2011	2	CS	1	Thienemanniella sp.	Orthocladiinae	Diptera	1	
6/24/2011	2	CS	1	Cladotanytarsus sp.	Chironominae	Diptera	6	
6/24/2011	2	CS	1	Tricorythodes sp.	Leptohyphidae	Ephemeroptera	9	
6/24/2011	2	CS	1	Chaetocladius sp.	Orthocladiinae	Diptera	1	
6/24/2011	2	CS	1	Neoperla osage	Perlidae	Plecoptera	5	
6/24/2011	2	CS	1	Acarina sp.	Hydracarina	Arachnoidea	17	
6/24/2011	2	CS	1	Isonychia bicolor	Isonychiidae	Ephemeroptera	2	
6/24/2011	2	CS	1	Argia sp.	Coengrionidae	Odonata	2	L/R
6/24/2011	2	CS	1	Maccaffertium sp.	Heptageniidae	Ephemeroptera	1	L/R
6/24/2011	2	CS	1	Ablabesmyia sp.	Tanypodinae	Diptera	2	
6/24/2011	2	CS	1	Polypedilum convictum	Chironominae	Diptera	14	
6/24/2011	2	CS	1	Lumbriculidae	Lumbriculidae	Lumbriculida	2	
6/24/2011	2	CS	1	Stylogomphus albistylus	Gomphidae	Odonata	1	L/R
6/24/2011	2	CS	1	Rheocricotopus sp.	Orthocladiinae	Diptera	2	
6/24/2011	2	CS	1	Orconectes peruncus	Cambaridae	Decapoda	2	L/R
6/24/2011	2	CS	1	Neoperla harpi	Perlidae	Plecoptera	5	
6/24/2011	2	CS	1	Cheumatopsyche sp.	Hydropsychidae	Tricoptera	37	
6/24/2011	2	CS	1	Microtendipes sp.	Chironominae	Diptera	3	
6/24/2011	2	CS	1	Phaenopsectra sp.	Chironominae	Diptera	20	
6/24/2011	2	CS	1	Corydalis sp.	Corydalidae	Megaloptera	1	L/R
6/24/2011	2	CS	1	Thienemannimyia grp	Tanypodinae	Diptera	14	
6/24/2011	2	CS	1	Orconectes peruncus	Cambaridae	Decapoda	3	
6/24/2011	2	CS	1	Stenacron sp.	Heptageniidae	Ephemeroptera	1	
6/24/2011	2	CS	1	Chironomidae Pupae	Chironomidae	Diptera	6	
6/24/2011	2	CS	1	Fallceon sp.	Baetidae	Ephemeroptera	4	
6/24/2011	2	CS	1	Maccaffertium sp.	Heptageniidae	Ephemeroptera	37	
6/24/2011	2	CS	1	Optioservus sp.	Elmidae	Coleoptera	21	
6/24/2011	2	CS	1	Chimarra sp.	Philopotamidae	Tricoptera	6	
6/24/2011	2	CS	1	Tubicidae	Tubicidae	Tubicidae	1	L/R
6/24/2011	2	CS	1	Stenonema femoratum	Heptageniidae	Ephemeroptera	2	
6/24/2011	2	CS	1	Simulium sp.	Simuliidae	Diptera	1	
6/24/2011	2	CS	1	Alloperla sp.	Chloroperlidae	Plecoptera	12	
6/24/2011	2	CS	1	Stenelmis sp.	Elmidae	Coleoptera	150	

S1 Appendix. Raw benthic macroinvertebrate data from Crane Pond Creek, Iron County, Missouri, USA.

6/24/2011	2	CS	1	Maccaffertium mediopunctatum	Heptageniidae	Ephemeroptera	14	
6/24/2011	2	CS	1	Stylogomphus albistylus	Gomphidae	Odonata	11	
6/24/2011	2	CS	1	Argia sp.	Coengrionidae	Odonata	1	
6/24/2011	2	CS	1	Nigronia sp.	Corydalidae	Megaloptera	4	
6/24/2011	2	CS	1	Corydalis sp.	Corydalidae	Megaloptera	1	
6/24/2011	2	CS	1	Lirceus sp.	Asellidae	Isopoda	2	
6/24/2011	2	CS	1	Baetidae	Baetidae	Ephemeroptera	4	
6/24/2011	2	CS	2	Orconectes sp.	Cambaridae	Decapoda	1	L/R
6/24/2011	2	CS	2	Maccaffertium sp.	Heptageniidae	Ephemeroptera	1	L/R
6/24/2011	2	CS	2	Stylogomphus albistylus	Gomphidae	Odonata	2	L/R
6/24/2011	2	CS	2	Isonychia bicolor	Isonychiidae	Ephemeroptera	4	L/R
6/24/2011	2	CS	2	Cheumatopsyche sp.	Hydropsychidae	Tricoptera	2	L/R
6/24/2011	2	CS	2	Nigronia sp.	Corydalidae	Megaloptera	3	L/R
6/24/2011	2	CS	2	Stenelmis lateralis	Elmidae	Coleoptera	1	L/R
6/24/2011	2	CS	2	Neoperla osage	Perlidae	Plecoptera	1	L/R
6/24/2011	2	CS	2	Thienemannimyia grp	Tanypodinae	Diptera	6	
6/24/2011	2	CS	2	Corydalis sp.	Corydalidae	Megaloptera	1	L/R
6/24/2011	2	CS	2	Chimarra sp.	Philopotamidae	Tricoptera	1	L/R
6/24/2011	2	CS	2	Hydropsyche sp.	Hydropsychidae	Tricoptera	3	
6/24/2011	2	CS	2	Alloperla sp.	Chloroperlidae	Plecoptera	68	ref
6/24/2011	2	CS	2	Orconectes peruncus	Cambaridae	Decapoda	1	
6/24/2011	2	CS	2	Neoperla sp.	Perlidae	Plecoptera	1	L/R
6/24/2011	2	CS	2	Chironomidae	Chironomidae	Diptera	219	
6/24/2011	2	CS	2	Chironomidae Pupae	Chironomidae	Diptera	7	
6/24/2011	2	CS	2	Thienemanniella sp.	Orthocladiinae	Diptera	1	
6/24/2011	2	CS	2	Cricotopus bicinctus	Orthocladiinae	Diptera	3	
6/24/2011	2	CS	2	Polypedilum (sp. A)	Chironominae	Diptera	17	
6/24/2011	2	CS	2	Cladotanytarsus sp.	Chironominae	Diptera	3	
6/24/2011	2	CS	2	Chaetocladius sp.	Orthocladiinae	Diptera	1	
6/24/2011	2	CS	2	Tanytarsus sp.	Chironominae	Diptera	112	
6/24/2011	2	CS	2	Ablabesmyia sp.	Tanypodinae	Diptera	1	
6/24/2011	2	CS	2	Polypedilum convictum	Chironominae	Diptera	20	
6/24/2011	2	CS	2	Rheotanytarsus sp.	Chironominae	Diptera	3	
6/24/2011	2	CS	2	Rheocricotopus sp.	Orthocladiinae	Diptera	9	
6/24/2011	2	CS	2	Neoperla harpi	Perlidae	Plecoptera	1	L/R
6/24/2011	2	CS	2	Maccaffertium mediopunctatum	Heptageniidae	Ephemeroptera	13	
6/24/2011	2	CS	2	Orconectes peruncus	Cambaridae	Decapoda	5	L/R

S1 Appendix. Raw benthic macroinvertebrate data from Crane Pond Creek, Iron County, Missouri, USA.

6/24/2011	2	CS	2	Simulium sp.	Simuliidae	Diptera	2
6/24/2011	2	CS	2	Serratella sp.	Ephemerellidae	Ephemeroptera	6
6/24/2011	2	CS	2	Cheumatopsyche sp.	Hydropsychidae	Tricoptera	125
6/24/2011	2	CS	2	Caenis sp.	Caenidae	Ephemeroptera	11
6/24/2011	2	CS	2	Nilotanypus sp.	Tanypodinae	Diptera	1
6/24/2011	2	CS	2	Chimarra sp.	Philopotamidae	Tricoptera	20
6/24/2011	2	CS	2	Stenelmis sp.	Elmidae	Coleoptera	39
6/24/2011	2	CS	2	Psephenus herricki	Psephenidae	Coleoptera	6
6/24/2011	2	CS	2	Optioservus sandersoni	Elmidae	Coleoptera	3
6/24/2011	2	CS	2	Corydalis sp.	Corydalidae	Megaloptera	3
6/24/2011	2	CS	2	Hemerodromia sp.	Empididae	Diptera	2
6/24/2011	2	CS	2	Tricorythodes sp.	Leptohyphidae	Ephemeroptera	10
6/24/2011	2	CS	2	Acarina sp.	Hydracarina	Arachnoidea	9
6/24/2011	2	CS	2	Maccaffertium vicarium	Heptageniidae	Ephemeroptera	4
6/24/2011	2	CS	2	Lirceus sp.	Asellidae	Isopoda	2
6/24/2011	2	CS	2	Helicopsyche sp.	Helicopsychidae	Tricoptera	3
6/24/2011	2	CS	2	Nigronia sp.	Corydalidae	Megaloptera	10
6/24/2011	2	CS	2	Maccaffertium sp.	Heptageniidae	Ephemeroptera	45
6/24/2011	2	CS	2	Stenelmis lateralis	Elmidae	Coleoptera	20
6/24/2011	2	CS	2	Fallceon sp.	Baetidae	Ephemeroptera	7
6/24/2011	2	CS	2	Neoperla sp.	Perlidae	Plecoptera	5
6/24/2011	2	CS	2	Stylogomphus albistylus	Gomphidae	Odonata	9
6/24/2011	2	CS	2	Isonychia bicolor	Isonychiidae	Ephemeroptera	13
6/24/2011	2	CS	2	Optioservus sp.	Elmidae	Coleoptera	11
6/24/2011	2	CS	2	Baetidae	Baetidae	Ephemeroptera	15
6/24/2011	2	CS	2	Amphinemura sp.	Nemouridae	Plecoptera	3
6/24/2011	2	CS	3	Amphinemura sp.	Nemouridae	Plecoptera	1
6/24/2011	2	CS	3	Neoperla sp.	Perlidae	Plecoptera	2
6/24/2011	2	CS	3	Nigronia sp.	Corydalidae	Megaloptera	3
6/24/2011	2	CS	3	Corydalis sp.	Corydalidae	Megaloptera	1
6/24/2011	2	CS	3	Stylogomphus albistylus	Gomphidae	Odonata	3
6/24/2011	2	CS	3	Neoperla harpi	Perlidae	Plecoptera	3
6/24/2011	2	CS	3	Neoperla osage	Perlidae	Plecoptera	2
6/24/2011	2	CS	3	Hydropsyche sp.	Hydropsychidae	Tricoptera	2
6/24/2011	2	CS	3	Psephenus herricki	Psephenidae	Coleoptera	2
6/24/2011	2	CS	3	Parakiefferiella sp.	Orthocladiinae	Diptera	1
6/24/2011	2	CS	3	Caenis sp.	Caenidae	Ephemeroptera	5

S1 Appendix. Raw benthic macroinvertebrate data from Crane Pond Creek, Iron County, Missouri, USA.

6/24/2011	2	CS	3	Orconectes peruncus	Cambaridae	Decapoda	1	
6/24/2011	2	CS	3	Stenelmis lateralis	Elmidae	Coleoptera	27	
6/24/2011	2	CS	3	Cheumatopsyche sp.	Hydropsychidae	Tricoptera	222	
6/24/2011	2	CS	3	Stenelmis sp.	Elmidae	Coleoptera	34	
6/24/2011	2	CS	3	Tricorythodes sp.	Leptohyphidae	Ephemeroptera	4	
6/24/2011	2	CS	3	Fallceon sp.	Baetidae	Ephemeroptera	25	
6/24/2011	2	CS	3	Baetidae	Baetidae	Ephemeroptera	18	
6/24/2011	2	CS	3	Orconectes sp.	Cambaridae	Decapoda	1	
6/24/2011	2	CS	3	Isonychia bicolor	Isonychiidae	Ephemeroptera	19	
6/24/2011	2	CS	3	Serratella sp.	Ephemerellidae	Ephemeroptera	5	
6/24/2011	2	CS	3	Chimarra sp.	Philopotamidae	Tricoptera	48	
6/24/2011	2	CS	3	Optioservus sp.	Elmidae	Coleoptera	2	
6/24/2011	2	CS	3	Simulium sp.	Simuliidae	Diptera	17	
6/24/2011	2	CS	3	Maccaffertium sp.	Heptageniidae	Ephemeroptera	41	
6/24/2011	2	CS	3	Alloperla sp.	Chloroperlidae	Plecoptera	6	
6/24/2011	2	CS	3	Maccaffertium mediopunctatum	Heptageniidae	Ephemeroptera	15	
6/24/2011	2	CS	3	Lumbriculidae	Lumbriculidae	Lumbriculida	1	
6/24/2011	2	CS	3	Thienemannimyia grp	Tanypodinae	Diptera	10	
6/24/2011	2	CS	3	Thienemanniella sp.	Orthocladiinae	Diptera	2	
6/24/2011	2	CS	3	Chironomidae	Chironomidae	Diptera	132	
6/24/2011	2	CS	3	Orconectes luteus	Cambaridae	Decapoda	3	L/R
6/24/2011	2	CS	3	Chaetocladius sp.	Orthocladiinae	Diptera	5	
6/24/2011	2	CS	3	Hydropsychidae Pupa	Hydropsychidae	Tricoptera	4	
6/24/2011	2	CS	3	Rheocricotopus sp.	Orthocladiinae	Diptera	10	
6/24/2011	2	CS	3	Hexatoma sp.	Tipulidae	Diptera	1	L/R
6/24/2011	2	CS	3	Isonychia bicolor	Isonychiidae	Ephemeroptera	6	L/R
6/24/2011	2	CS	3	Neoperla harpi	Perlidae	Plecoptera	3	L/R
6/24/2011	2	CS	3	Ablabesmyia sp.	Tanypodinae	Diptera	1	
6/24/2011	2	CS	3	Microtendipes sp.	Chironominae	Diptera	2	
6/24/2011	2	CS	3	Stylogomphus albistylus	Gomphidae	Odonata	3	L/R
6/24/2011	2	CS	3	Polypedilum convictum	Chironominae	Diptera	14	
6/24/2011	2	CS	3	Maccaffertium mediopunctatum	Heptageniidae	Ephemeroptera	2	L/R
6/24/2011	2	CS	3	Alloperla sp.	Chloroperlidae	Plecoptera	1	L/R
6/24/2011	2	CS	3	Corydalis sp.	Corydalidae	Megaloptera	5	L/R
6/24/2011	2	CS	3	Acarina sp.	Hydracarina	Arachnoidea	2	
6/24/2011	2	CS	3	Maccaffertium vicarium	Heptageniidae	Ephemeroptera	2	
6/24/2011	2	CS	3	Cricotopus bicinctus	Orthocladiinae	Diptera	4	

S1 Appendix. Raw benthic macroinvertebrate data from Crane Pond Creek, Iron County, Missouri, USA.

6/24/2011	2	CS	3	Neoperla harpi	Perlidae	Plecoptera	1	L/R
6/24/2011	2	CS	3	Psephenus herricki	Psephenidae	Coleoptera	1	L/R
6/24/2011	2	CS	3	Tanytarsus sp.	Chironominae	Diptera	38	
6/24/2011	2	CS	3	Orthocladius sp. (yellow dome tooth)	Orthocladiinae	Diptera	2	
6/24/2011	2	CS	3	Polypedilum (sp. A)	Chironominae	Diptera	13	
6/24/2011	2	CS	3	Chironomidae Pupae	Chironomidae	Diptera	10	
6/24/2011	2	NF	1	Neoperla sp.	Perlidae	Plecoptera	4	
6/24/2011	2	NF	1	Cryptochironomus sp.	Chironominae	Diptera	1	
6/24/2011	2	NF	1	Caenis sp.	Caenidae	Ephemeroptera	3	
6/24/2011	2	NF	1	Helicopsyche sp.	Helicopsychidae	Tricoptera	2	
6/24/2011	2	NF	1	Argia sp.	Coengrionidae	Odonata	2	
6/24/2011	2	NF	1	Tricorythodes sp.	Leptohyphidae	Ephemeroptera	2	
6/24/2011	2	NF	1	Choroterpes sp.	Leptophlebiidae	Ephemeroptera	5	
6/24/2011	2	NF	1	Polypedilum (sp. A)	Chironominae	Diptera	4	
6/24/2011	2	NF	1	Acarina sp.	Hydracarina	Arachnoidea	7	
6/24/2011	2	NF	1	Orconectes sp.	Cambaridae	Decapoda	1	
6/24/2011	2	NF	1	Parakiefferiella sp.	Orthocladiinae	Diptera	14	
6/24/2011	2	NF	1	Stylogomphus albistylus	Gomphidae	Odonata	1	
6/24/2011	2	NF	1	Serratella sp.	Ephemerellidae	Ephemeroptera	1	
6/24/2011	2	NF	1	Neoperla sp.	Perlidae	Plecoptera	1	L/R
6/24/2011	2	NF	1	Heterosternuta sp.	Dytiscidae	Coleoptera	3	
6/24/2011	2	NF	1	Stenonema femoratum	Heptageniidae	Ephemeroptera	1	L/R
6/24/2011	2	NF	1	Chironomidae Pupae	Chironomidae	Diptera	4	
6/24/2011	2	NF	1	Cricotopus bicinctus	Orthocladiinae	Diptera	4	
6/24/2011	2	NF	1	Cladotanytarsus sp.	Chironominae	Diptera	8	
6/24/2011	2	NF	1	Tanytarsus sp.	Chironominae	Diptera	96	
6/24/2011	2	NF	1	Ablabesmyia sp.	Tanypodinae	Diptera	10	
6/24/2011	2	NF	1	Chironomidae	Chironomidae	Diptera	212	
6/24/2011	2	NF	1	Helocordulia sp.	Corduliidae	Odonata	1	L/R
6/24/2011	2	NF	1	Stylogomphus albistylus	Gomphidae	Odonata	1	L/R
6/24/2011	2	NF	1	Ectopria nervosa	Psephenidae	Coleoptera	1	
6/24/2011	2	NF	1	Baetidae	Baetidae	Ephemeroptera	1	L/R
6/24/2011	2	NF	1	Argia sp.	Coengrionidae	Odonata	1	L/R
6/24/2011	2	NF	1	Psephenus herricki	Psephenidae	Coleoptera	5	
6/24/2011	2	NF	1	Oecetis sp.	Leptoceridae	Tricoptera	1	
6/24/2011	2	NF	1	Stenelmis sp.	Elmidae	Coleoptera	32	
6/24/2011	2	NF	1	Stenonema femoratum	Heptageniidae	Ephemeroptera	44	

S1 Appendix. Raw benthic macroinvertebrate data from Crane Pond Creek, Iron County, Missouri, USA.

6/24/2011	2	NF	1	Baetidae	Baetidae	Ephemeroptera	3	
6/24/2011	2	NF	1	Chimarra sp.	Philopotamidae	Tricoptera	1	L/R
6/24/2011	2	NF	1	Thienemannimyia grp	Tanypodinae	Diptera	3	
6/24/2011	2	NF	1	Dubiraphia sp.	Elmidae	Coleoptera	1	Larvae
6/24/2011	2	NF	1	Microtendipes sp.	Chironominae	Diptera	19	
6/24/2011	2	NF	1	Alloperla sp.	Chloroperlidae	Plecoptera	12	
6/24/2011	2	NF	1	Phaenopsectra sp.	Chironominae	Diptera	2	
6/24/2011	2	NF	1	Paratanytarsus sp.	Chironominae	Diptera	2	
6/24/2011	2	NF	1	Orthocladius sp. (yellow dome tooth)	Orthocladiinae	Diptera	2	
6/24/2011	2	NF	1	Lauterborniella sp.	Chironominae	Diptera	12	
6/24/2011	2	NF	2	Stylogomphus albistylus	Gomphidae	Odonata	1	
6/24/2011	2	NF	2	Stenelmis sp.	Elmidae	Coleoptera	37	
6/24/2011	2	NF	2	Microtendipes sp.	Chironominae	Diptera	8	
6/24/2011	2	NF	2	Acarina sp.	Hydracarina	Arachnoidea	3	
6/24/2011	2	NF	2	Lauterborniella sp.	Chironominae	Diptera	17	
6/24/2011	2	NF	2	Psephenus herricki	Psephenidae	Coleoptera	2	L/R
6/24/2011	2	NF	2	Bezzia sp.	Ceratopogonidae	Diptera	1	
6/24/2011	2	NF	2	Argia sp.	Coengrionidae	Odonata	4	L/R
6/24/2011	2	NF	2	Ectopria nervosa	Psephenidae	Coleoptera	1	
6/24/2011	2	NF	2	Psephenus herricki	Psephenidae	Coleoptera	1	
6/24/2011	2	NF	2	Hagenius brevistylus	Gomphidae	Odonata	1	
6/24/2011	2	NF	2	Neoperla sp.	Perlidae	Plecoptera	1	
6/24/2011	2	NF	2	Stenelmis lateralis	Elmidae	Coleoptera	6	
6/24/2011	2	NF	2	Caenis sp.	Caenidae	Ephemeroptera	1	
6/24/2011	2	NF	2	Choroterpes sp.	Leptophlebiidae	Ephemeroptera	9	
6/24/2011	2	NF	2	Fallceon sp.	Baetidae	Ephemeroptera	9	
6/24/2011	2	NF	2	Phaenopsectra sp.	Chironominae	Diptera	1	
6/24/2011	2	NF	2	Ablabesmyia sp.	Tanypodinae	Diptera	7	
6/24/2011	2	NF	2	Serratella sp.	Ephemerellidae	Ephemeroptera	1	
6/24/2011	2	NF	2	Cryptochironomus sp.	Chironominae	Diptera	4	
6/24/2011	2	NF	2	Corbicula sp.	Corbiculidae	Veroida	1	
6/24/2011	2	NF	2	Parakiefferiella sp.	Orthocladiinae	Diptera	23	
6/24/2011	2	NF	2	Paratanytarsus sp.	Chironominae	Diptera	2	
6/24/2011	2	NF	2	Dubiraphia sp.	Elmidae	Coleoptera	3	
6/24/2011	2	NF	2	Polypedilum (sp. A)	Chironominae	Diptera	6	
6/24/2011	2	NF	2	Cricotopus bicinctus	Orthocladiinae	Diptera	6	
6/24/2011	2	NF	2	Orconectes peruncus	Cambaridae	Decapoda	1	L/R

S1 Appendix. Raw benthic macroinvertebrate data from Crane Pond Creek, Iron County, Missouri, USA.

6/24/2011	2	NF	2	Chironomidae Pupae	Chironomidae	Diptera	6	
6/24/2011	2	NF	2	Libellula sp.	Libellulidae	Odonata	1	L/R
6/24/2011	2	NF	2	Orthocladius sp. (yellow dome tooth)	Orthocladiinae	Diptera	2	
6/24/2011	2	NF	2	Chironomidae	Chironomidae	Diptera	213	
6/24/2011	2	NF	2	Cladotanytarsus sp.	Chironominae	Diptera	17	
6/24/2011	2	NF	2	Tanytarsus sp.	Chironominae	Diptera	65	
6/24/2011	2	NF	2	Stenonema femoratum	Heptageniidae	Ephemeroptera	26	
6/24/2011	2	NF	2	Dicrotendipes sp.	Chironominae	Diptera	1	
6/24/2011	2	NF	2	Tricorythodes sp.	Leptohyphidae	Ephemeroptera	3	
6/24/2011	2	NF	2	Argia sp.	Coengrionidae	Odonata	1	
6/24/2011	2	NF	2	Stenonema femoratum	Heptageniidae	Ephemeroptera	6	L/R
6/24/2011	2	NF	3	Chironomidae	Chironomidae	Diptera	198	
6/24/2011	2	NF	3	Orconectes peruncus	Cambaridae	Decapoda	1	L/R
6/24/2011	2	NF	3	Phaenopsectra sp.	Chironominae	Diptera	55	
6/24/2011	2	NF	3	Procladius sp.	Tanypodinae	Diptera	5	
6/24/2011	2	NF	3	Libellula sp.	Libellulidae	Odonata	1	L/R
6/24/2011	2	NF	3	Chironomidae Pupae	Chironomidae	Diptera	3	
6/24/2011	2	NF	3	Parakiefferiella sp.	Orthocladiinae	Diptera	2	
6/24/2011	2	NF	3	Ablabesmyia sp.	Tanypodinae	Diptera	17	
6/24/2011	2	NF	3	Tanytarsus sp.	Chironominae	Diptera	33	
6/24/2011	2	NF	3	Cladotanytarsus sp.	Chironominae	Diptera	14	
6/24/2011	2	NF	3	Parachironomus sp.	Chironominae	Diptera	1	
6/24/2011	2	NF	3	Tricorythodes sp.	Leptohyphidae	Ephemeroptera	6	
6/24/2011	2	NF	3	Libellula sp.	Libellulidae	Odonata	1	
6/24/2011	2	NF	3	Bezzia sp.	Ceratopogonidae	Diptera	2	
6/24/2011	2	NF	3	Thienemanniella sp.	Orthocladiinae	Diptera	1	
6/24/2011	2	NF	3	Orconectes peruncus	Cambaridae	Decapoda	2	
6/24/2011	2	NF	3	Choroterpes sp.	Leptophlebiidae	Ephemeroptera	12	
6/24/2011	2	NF	3	Stenonema femoratum	Heptageniidae	Ephemeroptera	16	
6/24/2011	2	NF	3	Simulium sp.	Simuliidae	Diptera	1	
6/24/2011	2	NF	3	Acarina sp.	Hydracarina	Arachnoidea	21	
6/24/2011	2	NF	3	Baetidae	Baetidae	Ephemeroptera	1	
6/24/2011	2	NF	3	Corbicula sp.	Corbiculidae	Veroida	1	
6/24/2011	2	NF	3	Cryptochironomus sp.	Chironominae	Diptera	2	
6/24/2011	2	NF	3	Stenelmis sp.	Elmidae	Coleoptera	14	
6/24/2011	2	NF	3	Corbicula sp.	Corbiculidae	Veroida	1	
6/24/2011	2	NF	3	Paratanytarsus sp.	Chironominae	Diptera	1	

S1 Appendix. Raw benthic macroinvertebrate data from Crane Pond Creek, Iron County, Missouri, USA.

6/24/2011	2	NF	3	Stenelmis lateralis	Elmidae	Coleoptera	1	
6/24/2011	2	NF	3	Dubiraphia sp.	Elmidae	Coleoptera	7	Larvae
6/24/2011	2	NF	3	Tabanus sp.	Tabanidae	Diptera	2	
6/24/2011	2	NF	3	Psephenus herricki	Psephenidae	Coleoptera	3	
6/24/2011	2	NF	3	Trepobates becki	Gerridae	Hemiptera	2	
6/24/2011	2	NF	3	Oecetis sp.	Leptoceridae	Tricoptera	2	
6/24/2011	2	NF	3	Caenis sp.	Caenidae	Ephemeroptera	13	
6/24/2011	2	NF	3	Thienemannimyia grp	Tanypodinae	Diptera	3	
6/24/2011	2	NF	3	Serratella sp.	Ephemerellidae	Ephemeroptera	4	
6/24/2011	2	NF	3	Microtendipes sp.	Chironominae	Diptera	1	
6/24/2011	2	NF	3	Lauterborniella sp.	Chironominae	Diptera	10	
7/29/2011	1	CS	1	Baetidae	Baetidae	Ephemeroptera	3	
7/29/2011	1	CS	1	Chironomidae	Chironomidae	Diptera	19	
7/29/2011	1	CS	1	Serratella sp.	Ephemerellidae	Ephemeroptera	6	
7/29/2011	1	CS	1	Hexatoma sp.	Tipulidae	Diptera	2	
7/29/2011	1	CS	1	Corydalis sp.	Corydalidae	Megaloptera	11	
7/29/2011	1	CS	1	Petrophilia sp.	Pyralidae	Lepidoptera	1	
7/29/2011	1	CS	1	Stylogomphus albistylus	Gomphidae	Odonata	1	
7/29/2011	1	CS	1	Neoperla sp.	Perlidae	Plecoptera	17	
7/29/2011	1	CS	1	Acarina sp.	Hydracarina	Arachnoidea	21	
7/29/2011	1	CS	1	Argia sp.	Coengrionidae	Odonata	1	
7/29/2011	1	CS	1	Neoperla osage	Perlidae	Plecoptera	1	
7/29/2011	1	CS	1	Stenelmis lateralis	Elmidae	Coleoptera	36	
7/29/2011	1	CS	1	Maccaffertium mediopunctatum	Heptageniidae	Ephemeroptera	20	
7/29/2011	1	CS	1	Caenis sp.	Caenidae	Ephemeroptera	12	
7/29/2011	1	CS	1	Neoperla harpi	Perlidae	Plecoptera	1	
7/29/2011	1	CS	1	Maccaffertium sp.	Heptageniidae	Ephemeroptera	93	
7/29/2011	1	CS	1	Orconectes hylas	Cambaridae	Decapoda	13	L/R
7/29/2011	1	CS	1	Oecetis sp.	Leptoceridae	Tricoptera	4	
7/29/2011	1	CS	1	Orconectes hylas	Cambaridae	Decapoda	5	
7/29/2011	1	CS	1	Neoperla osage	Perlidae	Plecoptera	1	L/R
7/29/2011	1	CS	1	Trepobates becki	Gerridae	Hemiptera	1	L/R
7/29/2011	1	CS	1	Tricorythodes sp.	Leptohyphidae	Ephemeroptera	15	
7/29/2011	1	CS	1	Maccaffertium mediopunctatum	Heptageniidae	Ephemeroptera	3	L/R
7/29/2011	1	CS	1	Hexatoma sp.	Tipulidae	Diptera	1	L/R
7/29/2011	1	CS	1	Stenelmis sp.	Elmidae	Coleoptera	60	
7/29/2011	1	CS	1	Simulium sp.	Simuliidae	Diptera	2	

S1 Appendix. Raw benthic macroinvertebrate data from Crane Pond Creek, Iron County, Missouri, USA.

7/29/2011	1	CS	1	Helicopsyche sp.	Helicopsychidae	Tricoptera	4	
7/29/2011	1	CS	1	Neoperla harpi	Perlidae	Plecoptera	1	L/R
7/29/2011	1	CS	1	Optioservus sandersoni	Elmidae	Coleoptera	15	
7/29/2011	1	CS	1	Hydropsyche sp.	Hydropsychidae	Tricoptera	6	
7/29/2011	1	CS	1	Isonychia bicolor	Isonychiidae	Ephemeroptera	2	L/R
7/29/2011	1	CS	1	Corydalis sp.	Corydalidae	Megaloptera	10	L/R
7/29/2011	1	CS	1	Isonychia bicolor	Isonychiidae	Ephemeroptera	23	
7/29/2011	1	CS	1	Orconectes peruncus	Cambaridae	Decapoda	1	L/R
7/29/2011	1	CS	1	Stenonema femoratum	Heptageniidae	Ephemeroptera	1	
7/29/2011	1	CS	1	Tabanus sp.	Tabanidae	Diptera	1	
7/29/2011	1	CS	1	Optioservus sp.	Elmidae	Coleoptera	7	
7/29/2011	1	CS	1	Eukiefferiella sp.	Orthocladiinae	Diptera	1	
7/29/2011	1	CS	1	Cricotopus isocladius	Orthocladiinae	Diptera	1	
7/29/2011	1	CS	1	Thienemannimyia grp	Tanypodinae	Diptera	6	
7/29/2011	1	CS	1	Fallceon sp.	Baetidae	Ephemeroptera	4	
7/29/2011	1	CS	1	Polypedilum convictum	Chironominae	Diptera	3	
7/29/2011	1	CS	1	Paracladopelma sp.	Chironominae	Diptera	1	
7/29/2011	1	CS	1	Helichus sp.	Dryopidae	Coleoptera	5	Adult
7/29/2011	1	CS	1	Chimarra sp.	Philopotamidae	Tricoptera	1	
7/29/2011	1	CS	1	Polypedilum (sp. A)	Chironominae	Diptera	1	
7/29/2011	1	CS	1	Psephenus herricki	Psephenidae	Coleoptera	93	
7/29/2011	1	CS	1	Ectopria nervosa	Psephenidae	Coleoptera	2	
7/29/2011	1	CS	1	Choroterpes sp.	Leptophlebiidae	Ephemeroptera	1	
7/29/2011	1	CS	1	Cheumatopsyche sp.	Hydropsychidae	Tricoptera	61	
7/29/2011	1	CS	1	Cricotopus bicinctus	Orthocladiinae	Diptera	3	
7/29/2011	1	CS	2	Optioservus sp.	Elmidae	Coleoptera	6	
7/29/2011	1	CS	2	Argia sp.	Coengrionidae	Odonata	1	
7/29/2011	1	CS	2	Orconectes hylas	Cambaridae	Decapoda	1	
7/29/2011	1	CS	2	Fallceon sp.	Baetidae	Ephemeroptera	28	
7/29/2011	1	CS	2	Helichus sp.	Dryopidae	Coleoptera	2	
7/29/2011	1	CS	2	Isonychia bicolor	Isonychiidae	Ephemeroptera	41	
7/29/2011	1	CS	2	Bezzia sp.	Ceratopogonidae	Diptera	1	
7/29/2011	1	CS	2	Stenelmis lateralis	Elmidae	Coleoptera	70	
7/29/2011	1	CS	2	Maccaffertium mediopunctatum	Heptageniidae	Ephemeroptera	12	
7/29/2011	1	CS	2	Tricorythodes sp.	Leptohyphidae	Ephemeroptera	3	
7/29/2011	1	CS	2	Hydropsyche sp.	Hydropsychidae	Tricoptera	8	
7/29/2011	1	CS	2	Hexatoma sp.	Tipulidae	Diptera	1	

S1 Appendix. Raw benthic macroinvertebrate data from Crane Pond Creek, Iron County, Missouri, USA.

7/29/2011	1	CS	2	Serratella sp.	Ephemerellidae	Ephemeroptera	5	
7/29/2011	1	CS	2	Helicopsyche sp.	Helicopsychidae	Tricoptera	7	
7/29/2011	1	CS	2	Bezzia sp.	Ceratopogonidae	Diptera	1	
7/29/2011	1	CS	2	Maccaffertium sp.	Heptageniidae	Ephemeroptera	102	
7/29/2011	1	CS	2	Corydalis sp.	Corydalidae	Megaloptera	21	
7/29/2011	1	CS	2	Neoperla osage	Perlidae	Plecoptera	1	L/R
7/29/2011	1	CS	2	Atherix sp.	Athericidae	Diptera	2	L/R
7/29/2011	1	CS	2	Hetaerina sp.	Calopterygidae	Odonata	1	L/R
7/29/2011	1	CS	2	Corydalis sp.	Corydalidae	Megaloptera	3	L/R
7/29/2011	1	CS	2	Orconectes hylas	Cambaridae	Decapoda	19	L/R
7/29/2011	1	CS	2	Orconectes luteus	Cambaridae	Decapoda	4	
7/29/2011	1	CS	2	Chironomidae	Chironomidae	Diptera	19	
7/29/2011	1	CS	2	Acarina sp.	Hydracarina	Arachnoidea	10	
7/29/2011	1	CS	2	Caenis sp.	Caenidae	Ephemeroptera	14	
7/29/2011	1	CS	2	Agnetina flavescens	Perlidae	Plecoptera	12	
7/29/2011	1	CS	2	Nigronia sp.	Corydalidae	Megaloptera	9	
7/29/2011	1	CS	2	Optioservus sandersoni	Elmidae	Coleoptera	10	
7/29/2011	1	CS	2	Cheumatopsyche sp.	Hydropsychidae	Tricoptera	115	
7/29/2011	1	CS	2	Stenelmis sp.	Elmidae	Coleoptera	85	
7/29/2011	1	CS	2	Paratanytarsus sp.	Chironominae	Diptera	1	
7/29/2011	1	CS	2	Psephenus herricki	Psephenidae	Coleoptera	40	
7/29/2011	1	CS	2	Thienemannimyia grp	Tanypodinae	Diptera	1	
7/29/2011	1	CS	2	Polypedilum convictum	Chironominae	Diptera	6	
7/29/2011	1	CS	3	Cheumatopsyche sp.	Hydropsychidae	Tricoptera	110	
7/29/2011	1	CS	3	Corydalis sp.	Corydalidae	Megaloptera	11	L/R
7/29/2011	1	CS	3	Atherix sp.	Athericidae	Diptera	1	L/R
7/29/2011	1	CS	3	Maccaffertium mediopunctatum	Heptageniidae	Ephemeroptera	7	L/R
7/29/2011	1	CS	3	Isonychia bicolor	Isonychiidae	Ephemeroptera	3	L/R
7/29/2011	1	CS	3	Neoperla harpi	Perlidae	Plecoptera	2	L/R
7/29/2011	1	CS	3	Maccaffertium sp.	Heptageniidae	Ephemeroptera	32	
7/29/2011	1	CS	3	Orconectes hylas	Cambaridae	Decapoda	16	L/R
7/29/2011	1	CS	3	Atherix sp.	Athericidae	Diptera	1	
7/29/2011	1	CS	3	Chironomidae	Chironomidae	Diptera	19	
7/29/2011	1	CS	3	Baetis sp.	Baetidae	Ephemeroptera	3	
7/29/2011	1	CS	3	Helichus sp.	Dryopidae	Coleoptera	2	L/R
7/29/2011	1	CS	3	Optioservus sp.	Elmidae	Coleoptera	7	
7/29/2011	1	CS	3	Optioservus sandersoni	Elmidae	Coleoptera	15	

S1 Appendix. Raw benthic macroinvertebrate data from Crane Pond Creek, Iron County, Missouri, USA.

7/29/2011	1	CS	3	Bezzia sp.	Ceratopogonidae	Diptera	4
7/29/2011	1	CS	3	Chimarra sp.	Philopotamidae	Tricoptera	2
7/29/2011	1	CS	3	Marilia sp.	Odontoceridae	Tricoptera	1
7/29/2011	1	CS	3	Nigronia sp.	Corydalidae	Megaloptera	12
7/29/2011	1	CS	3	Isonychia bicolor	Isonychiidae	Ephemeroptera	37
7/29/2011	1	CS	3	Psephenus herricki	Psephenidae	Coleoptera	17
7/29/2011	1	CS	3	Helicopsyche sp.	Helicopsychidae	Tricoptera	3
7/29/2011	1	CS	3	Neoperla sp.	Perlidae	Plecoptera	10
7/29/2011	1	CS	3	Tricorythodes sp.	Leptohyphidae	Ephemeroptera	12
7/29/2011	1	CS	3	Stenelmis lateralis	Elmidae	Coleoptera	57
7/29/2011	1	CS	3	Serratella sp.	Ephemerellidae	Ephemeroptera	4
7/29/2011	1	CS	3	Siphlonurus sp.	Siphlonuridae	Ephemeroptera	3
7/29/2011	1	CS	3	Neoperla harpi	Perlidae	Plecoptera	1
7/29/2011	1	CS	3	Maccaffertium mediopunctatum	Heptageniidae	Ephemeroptera	9
7/29/2011	1	CS	3	Polypedilum (sp. A)	Chironominae	Diptera	21
7/29/2011	1	CS	3	Chironomidae Pupae	Chironomidae	Diptera	3
7/29/2011	1	CS	3	Rheotanytarsus sp.	Chironominae	Diptera	6
7/29/2011	1	CS	3	Thienemannimyia grp	Tanypodinae	Diptera	7
7/29/2011	1	CS	3	Corydalis sp.	Corydalidae	Megaloptera	15
7/29/2011	1	CS	3	Eukiefferiella sp.	Orthocladiinae	Diptera	1
7/29/2011	1	CS	3	Hydropsychidae	Hydropsychidae	Tricoptera	16
7/29/2011	1	CS	3	Thienemanniella sp.	Orthocladiinae	Diptera	6
7/29/2011	1	CS	3	Polypedilum convictum	Chironominae	Diptera	28
7/29/2011	1	CS	3	Cricotopus bicinctus	Orthocladiinae	Diptera	25
7/29/2011	1	CS	3	Caenis sp.	Caenidae	Ephemeroptera	17
7/29/2011	1	CS	3	Stenelmis sp.	Elmidae	Coleoptera	105
7/29/2011	1	CS	3	Tabanus sp.	Tabanidae	Diptera	6
7/29/2011	1	CS	3	Chaetocladius sp.	Orthocladiinae	Diptera	2
7/29/2011	1	CS	3	Elimia potosensis	Pleuroceridae	Gastropoda	13
7/29/2011	1	CS	3	Helichus sp.	Dryopidae	Coleoptera	3
7/29/2011	1	CS	3	Ochrotrichia sp.	Hydroptilidae	Tricoptera	1
7/29/2011	1	CS	3	Acarina sp.	Hydracarina	Arachnoidea	10
7/29/2011	1	CS	3	Orconectes hylas	Cambaridae	Decapoda	2
7/29/2011	1	CS	3	Cricotopus isocladius	Orthocladiinae	Diptera	15
7/29/2011	1	NF	1	Polypedilum convictum	Chironominae	Diptera	1
7/29/2011	1	NF	1	Ablabesmyia sp.	Tanypodinae	Diptera	4
7/29/2011	1	NF	1	Neoperla sp.	Perlidae	Plecoptera	8

S1 Appendix. Raw benthic macroinvertebrate data from Crane Pond Creek, Iron County, Missouri, USA.

7/29/2011	1	NF	1	Cladotanytarsus sp.	Chironominae	Diptera	9	
7/29/2011	1	NF	1	Corynoneura sp.	Orthocladiinae	Diptera	1	
7/29/2011	1	NF	1	Chironomidae Pupae	Chironomidae	Diptera	4	
7/29/2011	1	NF	1	Parakiefferiella sp.	Orthocladiinae	Diptera	4	
7/29/2011	1	NF	1	Tanytarsus sp.	Chironominae	Diptera	2	
7/29/2011	1	NF	1	Paratanytarsus sp.	Chironominae	Diptera	34	
7/29/2011	1	NF	1	Bezzia sp.	Ceratopogonidae	Diptera	2	
7/29/2011	1	NF	1	Argia sp.	Coengrionidae	Odonata	1	
7/29/2011	1	NF	1	Psephenus herricki	Psephenidae	Coleoptera	12	
7/29/2011	1	NF	1	Psephenus herricki	Psephenidae	Coleoptera	1	L/R
7/29/2011	1	NF	1	Neoperla sp.	Perlidae	Plecoptera	1	L/R
7/29/2011	1	NF	1	Chironomidae	Chironomidae	Diptera	117	
7/29/2011	1	NF	1	Stenelmis lateralis	Elmidae	Coleoptera	3	
7/29/2011	1	NF	1	Microtendipes sp.	Chironominae	Diptera	3	
7/29/2011	1	NF	1	Stenonema femoratum	Heptageniidae	Ephemeroptera	2	L/R
7/29/2011	1	NF	1	Orthocladius sp. (yellow dome tooth)	Orthocladiinae	Diptera	6	
7/29/2011	1	NF	1	Thienemannimyia grp	Tanypodinae	Diptera	3	
7/29/2011	1	NF	1	Dicrotendipes sp.	Chironominae	Diptera	4	
7/29/2011	1	NF	1	Lauterborniella sp.	Chironominae	Diptera	11	
7/29/2011	1	NF	1	Polypedilum (sp. A)	Chironominae	Diptera	1	
7/29/2011	1	NF	1	Optioservus sandersoni	Elmidae	Coleoptera	1	
7/29/2011	1	NF	1	Stenonema femoratum	Heptageniidae	Ephemeroptera	14	
7/29/2011	1	NF	1	Phaenopsectra sp.	Chironominae	Diptera	3	
7/29/2011	1	NF	1	Caenis sp.	Caenidae	Ephemeroptera	3	
7/29/2011	1	NF	1	Choroterpes sp.	Leptophlebiidae	Ephemeroptera	19	
7/29/2011	1	NF	1	Maccaffertium mediopunctatum	Heptageniidae	Ephemeroptera	2	
7/29/2011	1	NF	1	Stylogomphus albistylus	Gomphidae	Odonata	7	
7/29/2011	1	NF	1	Eukiefferiella sp.	Orthocladiinae	Diptera	1	
7/29/2011	1	NF	1	Polycentropus sp.	Polycentropodidae	Tricoptera	3	
7/29/2011	1	NF	1	Dubiraphia sp.	Elmidae	Coleoptera	21	
7/29/2011	1	NF	1	Atherix sp.	Athericidae	Diptera	1	
7/29/2011	1	NF	1	Cheumatopsyche sp.	Hydropsychidae	Tricoptera	8	
7/29/2011	1	NF	1	Acarina sp.	Hydracarina	Arachnoidea	4	
7/29/2011	1	NF	1	Ectopria nervosa	Psephenidae	Coleoptera	2	
7/29/2011	1	NF	1	Elimia potosensis	Pleuroceridae	Gastropoda	3	
7/29/2011	1	NF	1	Paralauterborniella sp.	Chironominae	Diptera	1	
7/29/2011	1	NF	1	Oecetis sp.	Leptoceridae	Tricoptera	3	

S1 Appendix. Raw benthic macroinvertebrate data from Crane Pond Creek, Iron County, Missouri, USA.

7/29/2011	1	NF	1	Stenelmis sp.	Elmidae	Coleoptera	73
7/29/2011	1	NF	1	Tricorythodes sp.	Leptohyphidae	Ephemeroptera	8
7/29/2011	1	NF	1	Baetisca sp.	Baetiscidae	Ephemeroptera	9
7/29/2011	1	NF	1	Chimarra sp.	Philopotamidae	Tricoptera	1
7/29/2011	1	NF	1	Optioservus sp.	Elmidae	Coleoptera	2
7/29/2011	1	NF	2	Chironomidae Pupae	Chironomidae	Diptera	8
7/29/2011	1	NF	2	Tabanus sp.	Tabanidae	Diptera	1
7/29/2011	1	NF	2	Ablabesmyia sp.	Tanypodinae	Diptera	5
7/29/2011	1	NF	2	Cryptochironomus sp.	Chironominae	Diptera	3
7/29/2011	1	NF	2	Cladotanytarsus sp.	Chironominae	Diptera	18
7/29/2011	1	NF	2	Dicrotendipes sp.	Chironominae	Diptera	4
7/29/2011	1	NF	2	Thienemannimyia grp	Tanypodinae	Diptera	2
7/29/2011	1	NF	2	Paralauterborniella sp.	Chironominae	Diptera	3
7/29/2011	1	NF	2	Cricotopus bicinctus	Orthocladiinae	Diptera	5
7/29/2011	1	NF	2	Paratanytarsus sp.	Chironominae	Diptera	11
7/29/2011	1	NF	2	Microtendipes sp.	Chironominae	Diptera	2
7/29/2011	1	NF	2	Lauterborniella sp.	Chironominae	Diptera	12
7/29/2011	1	NF	2	Tanytarsus sp.	Chironominae	Diptera	9
7/29/2011	1	NF	2	Stenelmis sp.	Elmidae	Coleoptera	80
7/29/2011	1	NF	2	Parakiefferiella sp.	Orthocladiinae	Diptera	15
7/29/2011	1	NF	2	Ectopria nervosa	Psephenidae	Coleoptera	2
7/29/2011	1	NF	2	Acarina sp.	Hydracarina	Arachnoidea	24
7/29/2011	1	NF	2	Hemerodromia sp.	Empididae	Diptera	4
7/29/2011	1	NF	2	Stenelmis lateralis	Elmidae	Coleoptera	2
7/29/2011	1	NF	2	Caenis sp.	Caenidae	Ephemeroptera	2
7/29/2011	1	NF	2	Elimia potosensis	Pleuroceridae	Gastropoda	2
7/29/2011	1	NF	2	Psephenus herricki	Psephenidae	Coleoptera	7
7/29/2011	1	NF	2	Cheumatopsyche sp.	Hydropsychidae	Tricoptera	2
7/29/2011	1	NF	2	Choroterpes sp.	Leptophlebiidae	Ephemeroptera	13
7/29/2011	1	NF	2	Dubiraphia sp.	Elmidae	Coleoptera	14
7/29/2011	1	NF	2	Baetidae	Baetidae	Ephemeroptera	5
7/29/2011	1	NF	2	Stenonema femoratum	Heptageniidae	Ephemeroptera	11
7/29/2011	1	NF	2	Oecetis sp.	Leptoceridae	Tricoptera	1
7/29/2011	1	NF	2	Argia sp.	Coengrionidae	Odonata	2
7/29/2011	1	NF	2	Bezzia sp.	Ceratopogonidae	Diptera	13
7/29/2011	1	NF	2	Trepobates becki	Gerridae	Hemiptera	1
7/29/2011	1	NF	2	Gyraulus sp.	Planorbidae	Gastropoda	1

S1 Appendix. Raw benthic macroinvertebrate data from Crane Pond Creek, Iron County, Missouri, USA.

7/29/2011	1	NF	2	Stenelmis sp.	Elmidae	Coleoptera	2	L/R
7/29/2011	1	NF	2	Stylogomphus albistylus	Gomphidae	Odonata	8	
7/29/2011	1	NF	2	Baetisca sp.	Baetiscidae	Ephemeroptera	12	
7/29/2011	1	NF	2	Tricorythodes sp.	Leptohyphidae	Ephemeroptera	4	
7/29/2011	1	NF	2	Chironomidae	Chironomidae	Diptera	115	
7/29/2011	1	NF	2	Choroterpes sp.	Leptophlebiidae	Ephemeroptera	2	L/R
7/29/2011	1	NF	2	Cheumatopsyche sp.	Hydropsychidae	Tricoptera	1	L/R
7/29/2011	1	NF	2	Tabanus sp.	Tabanidae	Diptera	1	L/R
7/29/2011	1	NF	3	Cladotanytarsus sp.	Chironominae	Diptera	17	
7/29/2011	1	NF	3	Chironomidae Pupae	Chironomidae	Diptera	4	
7/29/2011	1	NF	3	Dubiraphia sp.	Elmidae	Coleoptera	3	
7/29/2011	1	NF	3	Tanytarsus sp.	Chironominae	Diptera	6	
7/29/2011	1	NF	3	Cryptochironomus sp.	Chironominae	Diptera	2	
7/29/2011	1	NF	3	Dicrotendipes sp.	Chironominae	Diptera	6	
7/29/2011	1	NF	3	Cricotopus bicinctus	Orthocladiinae	Diptera	1	
7/29/2011	1	NF	3	Polypedilum convictum	Chironominae	Diptera	2	
7/29/2011	1	NF	3	Elimia potosensis	Pleuroceridae	Gastropoda	14	
7/29/2011	1	NF	3	Trepobates becki	Gerridae	Hemiptera	2	
7/29/2011	1	NF	3	Tricorythodes sp.	Leptohyphidae	Ephemeroptera	8	
7/29/2011	1	NF	3	Bezzia sp.	Ceratopogonidae	Diptera	8	
7/29/2011	1	NF	3	Stenelmis sp.	Elmidae	Coleoptera	58	
7/29/2011	1	NF	3	Tabanus sp.	Tabanidae	Diptera	2	
7/29/2011	1	NF	3	Neoperla sp.	Perlidae	Plecoptera	7	
7/29/2011	1	NF	3	Ectopria nervosa	Psephenidae	Coleoptera	1	
7/29/2011	1	NF	3	Stylogomphus albistylus	Gomphidae	Odonata	1	
7/29/2011	1	NF	3	Baetisca sp.	Baetiscidae	Ephemeroptera	2	
7/29/2011	1	NF	3	Stenelmis lateralis	Elmidae	Coleoptera	25	
7/29/2011	1	NF	3	Choroterpes sp.	Leptophlebiidae	Ephemeroptera	30	
7/29/2011	1	NF	3	Leuctra sp.	Leuctridae	Plecoptera	1	
7/29/2011	1	NF	3	Cheumatopsyche sp.	Hydropsychidae	Tricoptera	1	
7/29/2011	1	NF	3	Acarina sp.	Hydracarina	Arachnoidea	11	
7/29/2011	1	NF	3	Marilia sp.	Odontoceridae	Tricoptera	2	
7/29/2011	1	NF	3	Chironomidae	Chironomidae	Diptera	95	
7/29/2011	1	NF	3	Paratanytarsus sp.	Chironominae	Diptera	13	
7/29/2011	1	NF	3	Psephenus herricki	Psephenidae	Coleoptera	7	
7/29/2011	1	NF	3	Helicopsyche sp.	Helicopsychidae	Tricoptera	2	
7/29/2011	1	NF	3	Lauterborniella sp.	Chironominae	Diptera	37	

S1 Appendix. Raw benthic macroinvertebrate data from Crane Pond Creek, Iron County, Missouri, USA.

7/29/2011	1	NF	3	Stenonema femoratum	Heptageniidae	Ephemeroptera	19	
7/29/2011	2	CS	1	Chironomidae	Chironomidae	Diptera	33	
7/29/2011	2	CS	1	Orconectes sp.	Cambaridae	Decapoda	1	L/R
7/29/2011	2	CS	1	Ablabesmyia sp.	Tanypodinae	Diptera	2	
7/29/2011	2	CS	1	Chironomidae Pupae	Chironomidae	Diptera	3	
7/29/2011	2	CS	1	Tanytarsus sp.	Chironominae	Diptera	1	
7/29/2011	2	CS	1	Maccaffertium mediopunctatum	Heptageniidae	Ephemeroptera	6	
7/29/2011	2	CS	1	Tubificidae	Tubificidae	Tubificida	3	
7/29/2011	2	CS	1	Polypedilum convictum	Chironominae	Diptera	5	
7/29/2011	2	CS	1	Lauterborniella sp.	Chironominae	Diptera	1	
7/29/2011	2	CS	1	Thienemanniella sp.	Orthocladiinae	Diptera	6	
7/29/2011	2	CS	1	Phaenopsectra sp.	Chironominae	Diptera	1	
7/29/2011	2	CS	1	Thienemannimyia grp	Tanypodinae	Diptera	3	
7/29/2011	2	CS	1	Eukiefferiella sp.	Orthocladiinae	Diptera	1	
7/29/2011	2	CS	1	Rheotanytarsus sp.	Chironominae	Diptera	1	
7/29/2011	2	CS	1	Acarina sp.	Hydracarina	Arachnoidea	9	
7/29/2011	2	CS	1	Ectopria nervosa	Psephenidae	Coleoptera	2	
7/29/2011	2	CS	1	Isonychia bicolor	Isonychiidae	Ephemeroptera	44	
7/29/2011	2	CS	1	Optioservus sp.	Elmidae	Coleoptera	13	
7/29/2011	2	CS	1	Tabanus sp.	Tabanidae	Diptera	1	
7/29/2011	2	CS	1	Polycentropus sp.	Polycentropodidae	Tricoptera	2	
7/29/2011	2	CS	1	Orconectes luteus	Cambaridae	Decapoda	2	
7/29/2011	2	CS	1	Orconectes peruncus	Cambaridae	Decapoda	4	
7/29/2011	2	CS	1	Helicopsyche sp.	Helicopsychidae	Tricoptera	16	
7/29/2011	2	CS	1	Cheumatopsyche sp.	Hydropsychidae	Tricoptera	75	
7/29/2011	2	CS	1	Tricorythodes sp.	Leptohyphidae	Ephemeroptera	9	
7/29/2011	2	CS	1	Serratella sp.	Ephemerellidae	Ephemeroptera	10	
7/29/2011	2	CS	1	Polypedilum (sp. A)	Chironominae	Diptera	1	
7/29/2011	2	CS	1	Leuctra sp.	Leuctridae	Plecoptera	4	
7/29/2011	2	CS	1	Argia sp.	Coengrionidae	Odonata	2	
7/29/2011	2	CS	1	Neoperla sp.	Perlidae	Plecoptera	10	
7/29/2011	2	CS	1	Psephenus herricki	Psephenidae	Coleoptera	82	
7/29/2011	2	CS	1	Orconectes peruncus	Cambaridae	Decapoda	4	L/R
7/29/2011	2	CS	1	Cricotopus bicinctus	Orthocladiinae	Diptera	4	
7/29/2011	2	CS	1	Neoperla sp.	Perlidae	Plecoptera	10	
7/29/2011	2	CS	1	Stylogomphus albistylus	Gomphidae	Odonata	5	
7/29/2011	2	CS	1	Tabanus sp.	Tabanidae	Diptera	1	L/R

S1 Appendix. Raw benthic macroinvertebrate data from Crane Pond Creek, Iron County, Missouri, USA.

7/29/2011	2	CS	1	Neoperla harpi	Perlidae	Plecoptera	3	L/R
7/29/2011	2	CS	1	Rhagovelia sp.	Veliidae	Hemiptera	2	
7/29/2011	2	CS	1	Cladotanytarsus sp.	Chironominae	Diptera	2	
7/29/2011	2	CS	1	Chimarra sp.	Philopotamidae	Tricoptera	3	
7/29/2011	2	CS	1	Petrophilia sp.	Pyralidae	Lepidoptera	2	
7/29/2011	2	CS	1	Isonychia bicolor	Isonychiidae	Ephemeroptera	2	L/R
7/29/2011	2	CS	1	Neoperla harpi	Perlidae	Plecoptera	2	
7/29/2011	2	CS	1	Optioservus sandersoni	Elmidae	Coleoptera	3	
7/29/2011	2	CS	1	Maccaffertium mediopunctatum	Heptageniidae	Ephemeroptera	1	L/R
7/29/2011	2	CS	1	Caenis sp.	Caenidae	Ephemeroptera	10	
7/29/2011	2	CS	1	Stenelmis sp.	Elmidae	Coleoptera	143	
7/29/2011	2	CS	1	Stylogomphus albistylus	Gomphidae	Odonata	1	L/R
7/29/2011	2	CS	1	Nigronia sp.	Corydalidae	Megaloptera	5	
7/29/2011	2	CS	1	Bezzia sp.	Ceratopogonidae	Diptera	1	
7/29/2011	2	CS	1	Corydalis sp.	Corydalidae	Megaloptera	7	
7/29/2011	2	CS	1	Corydalis sp.	Corydalidae	Megaloptera	1	L/R
7/29/2011	2	CS	1	Maccaffertium sp.	Heptageniidae	Ephemeroptera	41	
7/29/2011	2	CS	2	Orconectes peruncus	Cambaridae	Decapoda	1	L/R
7/29/2011	2	CS	2	Chironomidae	Chironomidae	Diptera	46	
7/29/2011	2	CS	2	Corydalis sp.	Corydalidae	Megaloptera	3	L/R
7/29/2011	2	CS	2	Isonychia bicolor	Isonychiidae	Ephemeroptera	3	L/R
7/29/2011	2	CS	2	Bezzia sp.	Ceratopogonidae	Diptera	1	
7/29/2011	2	CS	2	Thienemanniella sp.	Orthocladiinae	Diptera	2	
7/29/2011	2	CS	2	Cryptochironomus sp.	Chironominae	Diptera	1	
7/29/2011	2	CS	2	Thienemannimyia grp	Tanypodinae	Diptera	9	
7/29/2011	2	CS	2	Phaenopsectra sp.	Chironominae	Diptera	2	
7/29/2011	2	CS	2	Maccaffertium sp.	Heptageniidae	Ephemeroptera	2	L/R
7/29/2011	2	CS	2	Nigronia sp.	Corydalidae	Megaloptera	5	
7/29/2011	2	CS	2	Polypedilum (sp. A)	Chironominae	Diptera	2	
7/29/2011	2	CS	2	Ablabesmyia sp.	Tanypodinae	Diptera	1	
7/29/2011	2	CS	2	Baetis sp.	Baetidae	Ephemeroptera	2	
7/29/2011	2	CS	2	Rhagovelia sp.	Veliidae	Hemiptera	1	
7/29/2011	2	CS	2	Chironomidae Pupae	Chironomidae	Diptera	5	
7/29/2011	2	CS	2	Maccaffertium vicarium	Heptageniidae	Ephemeroptera	1	
7/29/2011	2	CS	2	Neoperla sp.	Perlidae	Plecoptera	4	
7/29/2011	2	CS	2	Tricorythodes sp.	Leptohyphidae	Ephemeroptera	18	
7/29/2011	2	CS	2	Acarina sp.	Hydracarina	Arachnoidea	11	

S1 Appendix. Raw benthic macroinvertebrate data from Crane Pond Creek, Iron County, Missouri, USA.

7/29/2011	2	CS	2	Isonychia bicolor	Isonychiidae	Ephemeroptera	43	
7/29/2011	2	CS	2	Leuctra sp.	Leuctridae	Plecoptera	15	
7/29/2011	2	CS	2	Hydroptila	Hyrdroptilidae	Tricoptera	1	
7/29/2011	2	CS	2	Stylogomphus albistylus	Gomphidae	Odonata	30	
7/29/2011	2	CS	2	Argia sp.	Coengrionidae	Odonata	5	
7/29/2011	2	CS	2	Petrophilia sp.	Pyralidae	Lepidoptera	1	
7/29/2011	2	CS	2	Helicopsyche sp.	Helicopsychidae	Tricoptera	7	
7/29/2011	2	CS	2	Ectopria nervosa	Psephenidae	Coleoptera	1	
7/29/2011	2	CS	2	Stenelmis sp.	Elmidae	Coleoptera	132	
7/29/2011	2	CS	2	Cheumatopsyche sp.	Hydropsychidae	Tricoptera	67	
7/29/2011	2	CS	2	Psephenus herricki	Psephenidae	Coleoptera	116	
7/29/2011	2	CS	2	Tabanus sp.	Tabanidae	Diptera	3	
7/29/2011	2	CS	2	Maccaffertium mediopunctatum	Heptageniidae	Ephemeroptera	3	L/R
7/29/2011	2	CS	2	Tipula sp.	Tipulidae	Diptera	1	L/R
7/29/2011	2	CS	2	Chimarra sp.	Philopotamidae	Tricoptera	9	
7/29/2011	2	CS	2	Optioservus sp.	Elmidae	Coleoptera	8	
7/29/2011	2	CS	2	Cricotopus bicinctus	Orthocladiinae	Diptera	8	
7/29/2011	2	CS	2	Maccaffertium mediopunctatum	Heptageniidae	Ephemeroptera	5	
7/29/2011	2	CS	2	Rheocricotopus sp.	Orthocladiinae	Diptera	1	
7/29/2011	2	CS	2	Stenelmis lateralis	Elmidae	Coleoptera	21	
7/29/2011	2	CS	2	Neoperla harpi	Perlidae	Plecoptera	1	
7/29/2011	2	CS	2	Stylogomphus albistylus	Gomphidae	Odonata	1	L/R
7/29/2011	2	CS	2	Corydalis sp.	Corydalidae	Megaloptera	5	
7/29/2011	2	CS	2	Bezzia sp.	Ceratopogonidae	Diptera	1	
7/29/2011	2	CS	2	Caenis sp.	Caenidae	Ephemeroptera	52	
7/29/2011	2	CS	2	Polypedilum convictum	Chironominae	Diptera	4	
7/29/2011	2	CS	2	Lumbriculidae	Lumbriculidae	Lumbriculida	5	
7/29/2011	2	CS	2	Polycentropus sp.	Polycentropodidae	Tricoptera	3	
7/29/2011	2	CS	2	Maccaffertium sp.	Heptageniidae	Ephemeroptera	64	
7/29/2011	2	CS	3	Maccaffertium sp.	Heptageniidae	Ephemeroptera	46	
7/29/2011	2	CS	3	Cricotopus bicinctus	Orthocladiinae	Diptera	7	
7/29/2011	2	CS	3	Corydalis sp.	Corydalidae	Megaloptera	4	L/R
7/29/2011	2	CS	3	Neoperla harpi	Perlidae	Plecoptera	2	L/R
7/29/2011	2	CS	3	Tipula sp.	Tipulidae	Diptera	1	L/R
7/29/2011	2	CS	3	Hexatoma sp.	Tipulidae	Diptera	1	L/R
7/29/2011	2	CS	3	Orconectes peruncus	Cambaridae	Decapoda	2	L/R
7/29/2011	2	CS	3	Microtendipes sp.	Chironominae	Diptera	1	

S1 Appendix. Raw benthic macroinvertebrate data from Crane Pond Creek, Iron County, Missouri, USA.

7/29/2011	2	CS	3	Thienemannimyia grp	Tanypodinae	Diptera	8
7/29/2011	2	CS	3	Phaenopsectra sp.	Chironominae	Diptera	1
7/29/2011	2	CS	3	Psephenus herricki	Psephenidae	Coleoptera	12
7/29/2011	2	CS	3	Thienemanniella sp.	Orthocladiinae	Diptera	6
7/29/2011	2	CS	3	Acarina sp.	Hydracarina	Arachnoidea	7
7/29/2011	2	CS	3	Chironomidae	Chironomidae	Diptera	102
7/29/2011	2	CS	3	Polypedilum (sp. A)	Chironominae	Diptera	7
7/29/2011	2	CS	3	Cladotanytarsus sp.	Chironominae	Diptera	2
7/29/2011	2	CS	3	Chironomidae Pupae	Chironomidae	Diptera	5
7/29/2011	2	CS	3	Bezzia sp.	Ceratopogonidae	Diptera	2
7/29/2011	2	CS	3	Polypedilum convictum	Chironominae	Diptera	2
7/29/2011	2	CS	3	Rheocricotopus sp.	Orthocladiinae	Diptera	2
7/29/2011	2	CS	3	Rheotanytarsus sp.	Chironominae	Diptera	3
7/29/2011	2	CS	3	Eukiefferiella sp.	Orthocladiinae	Diptera	1
7/29/2011	2	CS	3	Stenelmis sp.	Elmidae	Coleoptera	51
7/29/2011	2	CS	3	Tabanus sp.	Tabanidae	Diptera	4
7/29/2011	2	CS	3	Optioservus sandersoni	Elmidae	Coleoptera	4
7/29/2011	2	CS	3	Hydropsychidae Pupa	Hydropsychidae	Tricoptera	1
7/29/2011	2	CS	3	Optioservus sp.	Elmidae	Coleoptera	3
7/29/2011	2	CS	3	Isonychia bicolor	Isonychiidae	Ephemeroptera	116
7/29/2011	2	CS	3	Atherix sp.	Athericidae	Diptera	1
7/29/2011	2	CS	3	Corydalis sp.	Corydalidae	Megaloptera	27
7/29/2011	2	CS	3	Chimarra sp.	Philopotamidae	Tricoptera	32
7/29/2011	2	CS	3	Orconectes peruncus	Cambaridae	Decapoda	2
7/29/2011	2	CS	3	Neoperla harpi	Perlidae	Plecoptera	1
7/29/2011	2	CS	3	Cheumatopsyche sp.	Hydropsychidae	Tricoptera	126
7/29/2011	2	CS	3	Hydropsyche sp.	Hydropsychidae	Tricoptera	4
7/29/2011	2	CS	3	Neoperla sp.	Perlidae	Plecoptera	3
7/29/2011	2	CS	3	Ochrotrichia sp.	Hydroptilidae	Tricoptera	1
7/29/2011	2	CS	3	Caenis sp.	Caenidae	Ephemeroptera	39
7/29/2011	2	CS	3	Nigronia sp.	Corydalidae	Megaloptera	1
7/29/2011	2	CS	3	Baetis sp.	Baetidae	Ephemeroptera	10
7/29/2011	2	CS	3	Maccaffertium mediopunctatum	Heptageniidae	Ephemeroptera	8
7/29/2011	2	CS	3	Tricorythodes sp.	Leptohyphidae	Ephemeroptera	2
7/29/2011	2	CS	3	Leuctra sp.	Leuctridae	Plecoptera	2
7/29/2011	2	CS	3	Stenelmis lateralis	Elmidae	Coleoptera	64
7/29/2011	2	NF	1	Tubificidae	Tubificidae	Tubificida	1

S1 Appendix. Raw benthic macroinvertebrate data from Crane Pond Creek, Iron County, Missouri, USA.

7/29/2011	2	NF	1	Lauterborniella sp.	Chironominae	Diptera	1	
7/29/2011	2	NF	1	Stenonema femoratum	Heptageniidae	Ephemeroptera	3	
7/29/2011	2	NF	1	Paralauterborniella sp.	Chironominae	Diptera	8	
7/29/2011	2	NF	1	Choroterpes sp.	Leptophlebiidae	Ephemeroptera	4	
7/29/2011	2	NF	1	Chironomidae	Chironomidae	Diptera	111	
7/29/2011	2	NF	1	Nanocladius sp.	Orthocladiinae	Diptera	1	
7/29/2011	2	NF	1	Thienemannimyia grp	Tanypodinae	Diptera	1	
7/29/2011	2	NF	1	Baetisca sp.	Baetiscidae	Ephemeroptera	2	
7/29/2011	2	NF	1	Stenelmis sp.	Elmidae	Coleoptera	2	L/R
7/29/2011	2	NF	1	Orthocladius sp. (yellow dome tooth)	Orthocladiinae	Diptera	3	
7/29/2011	2	NF	1	Psephenus herricki	Psephenidae	Coleoptera	1	
7/29/2011	2	NF	1	Stenelmis sp.	Elmidae	Coleoptera	30	
7/29/2011	2	NF	1	Phaenopsectra sp.	Chironominae	Diptera	1	
7/29/2011	2	NF	1	Cryptochironomus sp.	Chironominae	Diptera	1	
7/29/2011	2	NF	1	Cricotopus bicinctus	Orthocladiinae	Diptera	4	
7/29/2011	2	NF	1	Cladotanytarsus sp.	Chironominae	Diptera	2	
7/29/2011	2	NF	1	Nilotanypus sp.	Tanypodinae	Diptera	1	
7/29/2011	2	NF	1	Ablabesmyia sp.	Tanypodinae	Diptera	13	
7/29/2011	2	NF	1	Paracladopelma sp.	Chironominae	Diptera	2	
7/29/2011	2	NF	1	Parakiefferiella sp.	Orthocladiinae	Diptera	27	
7/29/2011	2	NF	1	Tanytarsus sp.	Chironominae	Diptera	7	
7/29/2011	2	NF	1	Baetidae	Baetidae	Ephemeroptera	2	
7/29/2011	2	NF	1	Heptageniidae	Heptageniidae	Ephemeroptera	3	
7/29/2011	2	NF	1	Bezzia sp.	Ceratopogonidae	Diptera	6	
7/29/2011	2	NF	1	Choroterpes sp.	Leptophlebiidae	Ephemeroptera	25	
7/29/2011	2	NF	1	Caenis sp.	Caenidae	Ephemeroptera	45	
7/29/2011	2	NF	1	Polycentropus sp.	Polycentropodidae	Tricoptera	2	
7/29/2011	2	NF	1	Stylogomphus albistylus	Gomphidae	Odonata	10	
7/29/2011	2	NF	1	Helicopsyche sp.	Helicopsychidae	Tricoptera	1	
7/29/2011	2	NF	1	Oecetis sp.	Leptoceridae	Tricoptera	10	
7/29/2011	2	NF	1	Stylogomphus albistylus	Gomphidae	Odonata	1	
7/29/2011	2	NF	1	Tipula sp.	Tipulidae	Diptera	1	
7/29/2011	2	NF	1	Caenis sp.	Caenidae	Ephemeroptera	1	
7/29/2011	2	NF	1	Stenonema femoratum	Heptageniidae	Ephemeroptera	36	
7/29/2011	2	NF	1	Argia sp.	Coengrionidae	Odonata	5	
7/29/2011	2	NF	1	Acarina sp.	Hydracarina	Arachnoidea	9	
7/29/2011	2	NF	1	Leuctra sp.	Leuctridae	Plecoptera	16	

S1 Appendix. Raw benthic macroinvertebrate data from Crane Pond Creek, Iron County, Missouri, USA.

7/29/2011	2	NF	1	Neoperla sp.	Perlidae	Plecoptera	2	
7/29/2011	2	NF	1	Ectopria nervosa	Psephenidae	Coleoptera	10	
7/29/2011	2	NF	1	Chironomidae Pupae	Chironomidae	Diptera	4	
7/29/2011	2	NF	1	Stenelmis lateralis	Elmidae	Coleoptera	3	
7/29/2011	2	NF	1	Neoperla sp.	Perlidae	Plecoptera	3	
7/29/2011	2	NF	1	Gyraulus sp.	Planorbidae	Gastropoda	2	
7/29/2011	2	NF	2	Thienemannimyia grp	Tanypodinae	Diptera	1	
7/29/2011	2	NF	2	Thienemanniella sp.	Orthocladiinae	Diptera	2	
7/29/2011	2	NF	2	Paratanytarsus sp.	Chironominae	Diptera	3	
7/29/2011	2	NF	2	Paralauterborniella sp.	Chironominae	Diptera	1	
7/29/2011	2	NF	2	Parakiefferiella sp.	Orthocladiinae	Diptera	4	
7/29/2011	2	NF	2	Ablabesmyia sp.	Tanypodinae	Diptera	8	
7/29/2011	2	NF	2	Cladotanytarsus sp.	Chironominae	Diptera	2	
7/29/2011	2	NF	2	Cricotopus bicinctus	Orthocladiinae	Diptera	3	
7/29/2011	2	NF	2	Parachironomus sp.	Chironominae	Diptera	1	
7/29/2011	2	NF	2	Cryptochironomus sp.	Chironominae	Diptera	1	
7/29/2011	2	NF	2	Dicrotendipes sp.	Chironominae	Diptera	1	
7/29/2011	2	NF	2	Lauterborniella sp.	Chironominae	Diptera	2	
7/29/2011	2	NF	2	Chironomidae Pupae	Chironomidae	Diptera	4	
7/29/2011	2	NF	2	Nanocladius sp.	Orthocladiinae	Diptera	1	
7/29/2011	2	NF	2	Acarina sp.	Hydracarina	Arachnoidea	9	
7/29/2011	2	NF	2	Orthocladius sp. (yellow dome tooth)	Orthocladiinae	Diptera	1	
7/29/2011	2	NF	2	Argia sp.	Coengrionidae	Odonata	10	
7/29/2011	2	NF	2	Choroterpes sp.	Leptophlebiidae	Ephemeroptera	1	L/R
7/29/2011	2	NF	2	Tabanus sp.	Tabanidae	Diptera	1	
7/29/2011	2	NF	2	Psephenus herricki	Psephenidae	Coleoptera	4	
7/29/2011	2	NF	2	Stenonema femoratum	Heptageniidae	Ephemeroptera	62	
7/29/2011	2	NF	2	Heptageniidae	Heptageniidae	Ephemeroptera	2	
7/29/2011	2	NF	2	Dubiraphia sp.	Elmidae	Coleoptera	14	
7/29/2011	2	NF	2	Choroterpes sp.	Leptophlebiidae	Ephemeroptera	16	
7/29/2011	2	NF	2	Neoperla sp.	Perlidae	Plecoptera	1	
7/29/2011	2	NF	2	Gyraulus sp.	Planorbidae	Gastropoda	1	
7/29/2011	2	NF	2	Stenelmis lateralis	Elmidae	Coleoptera	1	
7/29/2011	2	NF	2	Dromogomphus sp.	Gomphidae	Odonata	1	
7/29/2011	2	NF	2	Baetidae	Baetidae	Ephemeroptera	4	
7/29/2011	2	NF	2	Ectopria nervosa	Psephenidae	Coleoptera	31	
7/29/2011	2	NF	2	Bezzia sp.	Ceratopogonidae	Diptera	7	

S1 Appendix. Raw benthic macroinvertebrate data from Crane Pond Creek, Iron County, Missouri, USA.

7/29/2011	2	NF	2	Oecetis sp.	Leptoceridae	Tricoptera	1	
7/29/2011	2	NF	2	Polycentropus sp.	Polycentropodidae	Tricoptera	1	
7/29/2011	2	NF	2	Caenis sp.	Caenidae	Ephemeroptera	81	
7/29/2011	2	NF	2	Stenonema femoratum	Heptageniidae	Ephemeroptera	9	L/R
7/29/2011	2	NF	2	Polypedilum (sp. A)	Chironominae	Diptera	2	
7/29/2011	2	NF	2	Polypedilum convictum	Chironominae	Diptera	1	
7/29/2011	2	NF	2	Tanytarsus sp.	Chironominae	Diptera	8	
7/29/2011	2	NF	2	Chironomidae	Chironomidae	Diptera	50	
7/29/2011	2	NF	2	Stenelmis sp.	Elmidae	Coleoptera	19	
7/29/2011	2	NF	2	Ectopria nervosa	Psephenidae	Coleoptera	1	L/R
7/29/2011	2	NF	2	Psephenus herricki	Psephenidae	Coleoptera	1	L/R
7/29/2011	2	NF	2	Gammarus sp.	Gammaridae	Amphipoda	1	
7/29/2011	2	NF	2	Fallceon sp.	Baetidae	Ephemeroptera	7	
7/29/2011	2	NF	3	Paratanytarsus sp.	Chironominae	Diptera	1	
7/29/2011	2	NF	3	Chironomidae Pupae	Chironomidae	Diptera	3	
7/29/2011	2	NF	3	Stenelmis lateralis	Elmidae	Coleoptera	2	
7/29/2011	2	NF	3	Paralauterborniella sp.	Chironominae	Diptera	2	
7/29/2011	2	NF	3	Phaenopsectra sp.	Chironominae	Diptera	8	
7/29/2011	2	NF	3	Orthocladius sp. (yellow dome tooth)	Orthocladiinae	Diptera	1	
7/29/2011	2	NF	3	Paracladopelma sp.	Chironominae	Diptera	2	
7/29/2011	2	NF	3	Tanytarsus sp.	Chironominae	Diptera	13	
7/29/2011	2	NF	3	Parakiefferiella sp.	Orthocladiinae	Diptera	2	
7/29/2011	2	NF	3	Polypedilum (sp. A)	Chironominae	Diptera	1	
7/29/2011	2	NF	3	Thienemannimyia grp	Tanypodinae	Diptera	1	
7/29/2011	2	NF	3	Stenonema femoratum	Heptageniidae	Ephemeroptera	19	
7/29/2011	2	NF	3	Baetisca sp.	Baetiscidae	Ephemeroptera	1	
7/29/2011	2	NF	3	Helicopsyche sp.	Helicopsychidae	Tricoptera	1	
7/29/2011	2	NF	3	Stenelmis sp.	Elmidae	Coleoptera	36	
7/29/2011	2	NF	3	Fallceon sp.	Baetidae	Ephemeroptera	19	
7/29/2011	2	NF	3	Caenis sp.	Caenidae	Ephemeroptera	123	
7/29/2011	2	NF	3	Argia sp.	Coengrionidae	Odonata	3	
7/29/2011	2	NF	3	Tabanus sp.	Tabanidae	Diptera	1	
7/29/2011	2	NF	3	Oecetis sp.	Leptoceridae	Tricoptera	3	
7/29/2011	2	NF	3	Dromogomphus sp.	Gomphidae	Odonata	1	
7/29/2011	2	NF	3	Acarina sp.	Hydracarina	Arachnoidea	6	
7/29/2011	2	NF	3	Bezzia sp.	Ceratopogonidae	Diptera	2	
7/29/2011	2	NF	3	Choroterpes sp.	Leptophlebiidae	Ephemeroptera	11	

S1 Appendix. Raw benthic macroinvertebrate data from Crane Pond Creek, Iron County, Missouri, USA.

7/29/2011	2	NF	3	Leuctra sp.	Leuctridae	Plecoptera	1	
7/29/2011	2	NF	3	Ectopria nervosa	Psephenidae	Coleoptera	3	
7/29/2011	2	NF	3	Stenelmis sp.	Elmidae	Coleoptera	2	L/R
7/29/2011	2	NF	3	Dicrotendipes sp.	Chironominae	Diptera	1	
7/29/2011	2	NF	3	Cryptochironomus sp.	Chironominae	Diptera	1	
7/29/2011	2	NF	3	Cladotanytarsus sp.	Chironominae	Diptera	14	
7/29/2011	2	NF	3	Ablabesmyia sp.	Tanypodinae	Diptera	2	
7/29/2011	2	NF	3	Chironomidae	Chironomidae	Diptera	81	
7/29/2011	2	NF	3	Procladius sp.	Tanypodinae	Diptera	1	
7/29/2011	2	NF	3	Corduliidae	Corduliidae	Odonata	1	L/R
7/29/2011	2	NF	3	Lauterborniella sp.	Chironominae	Diptera	9	
7/29/2011	2	NF	3	Stenonema femoratum	Heptageniidae	Ephemeroptera	10	L/R
7/29/2011	2	NF	3	Psephenus herricki	Psephenidae	Coleoptera	3	L/R
7/29/2011	2	NF	3	Caenis sp.	Caenidae	Ephemeroptera	1	L/R
7/29/2011	2	NF	3	Dubiraphia sp.	Elmidae	Coleoptera	16	
7/29/2011	2	NF	3	Psephenus herricki	Psephenidae	Coleoptera	2	
9/30/2011	1	CS	1	Fallceon sp.	Baetidae	Ephemeroptera	3	
9/30/2011	1	CS	1	Paratanytarsus sp.	Chironominae	Diptera	2	
9/30/2011	1	CS	1	Eukiefferiella sp.	Orthocladiinae	Diptera	1	
9/30/2011	1	CS	1	Polypedilum (sp. A)	Chironominae	Diptera	1	
9/30/2011	1	CS	1	Ectopria nervosa	Psephenidae	Coleoptera	6	
9/30/2011	1	CS	1	Tabanus sp.	Tabanidae	Diptera	1	
9/30/2011	1	CS	1	Neoperla harpi	Perlidae	Plecoptera	16	
9/30/2011	1	CS	1	Maccaffertium sp.	Heptageniidae	Ephemeroptera	20	
9/30/2011	1	CS	1	Elimia potosensis	Pleuroceridae	Gastropoda	59	
9/30/2011	1	CS	1	Caenis sp.	Caenidae	Ephemeroptera	6	
9/30/2011	1	CS	1	Optioservus sandersoni	Elmidae	Coleoptera	4	
9/30/2011	1	CS	1	Tricorythodes sp.	Leptohyphidae	Ephemeroptera	1	
9/30/2011	1	CS	1	Simulium sp.	Simuliidae	Diptera	4	
9/30/2011	1	CS	1	Stenelmis lateralis	Elmidae	Coleoptera	100	
9/30/2011	1	CS	1	Helicopsyche sp.	Helicopsychidae	Tricoptera	2	
9/30/2011	1	CS	1	Psephenus herricki	Psephenidae	Coleoptera	129	
9/30/2011	1	CS	1	Neoperla sp.	Perlidae	Plecoptera	13	
9/30/2011	1	CS	1	Stenonema femoratum	Heptageniidae	Ephemeroptera	2	
9/30/2011	1	CS	1	Maccaffertium mediopunctatum	Heptageniidae	Ephemeroptera	35	
9/30/2011	1	CS	1	Neoperla osage	Perlidae	Plecoptera	10	
9/30/2011	1	CS	1	Isonychia bicolor	Isonychiidae	Ephemeroptera	21	

S1 Appendix. Raw benthic macroinvertebrate data from Crane Pond Creek, Iron County, Missouri, USA.

9/30/2011	1	CS	1	Polycentropus sp.	Polycentropodidae	Tricoptera	2	
9/30/2011	1	CS	1	Optioservus sp.	Elmidae	Coleoptera	13	
9/30/2011	1	CS	1	Atherix sp.	Athericidae	Diptera	1	
9/30/2011	1	CS	1	Petrophilia sp.	Pyralidae	Lepidoptera	1	
9/30/2011	1	CS	1	Acarina sp.	Hydracarina	Arachnoidea	14	
9/30/2011	1	CS	1	Atherix sp.	Athericidae	Diptera	3	L/R
9/30/2011	1	CS	1	Chironomidae Pupae	Chironomidae	Diptera	1	
9/30/2011	1	CS	1	Chironomidae	Chironomidae	Diptera	9	
9/30/2011	1	CS	1	Orconectes hylas	Cambaridae	Decapoda	4	L/R
9/30/2011	1	CS	1	Elimia potosensis	Pleuroceridae	Gastropoda	1	L/R
9/30/2011	1	CS	1	Stenelmis sp.	Elmidae	Coleoptera	93	
9/30/2011	1	CS	1	Tabanus sp.	Tabanidae	Diptera	1	L/R
9/30/2011	1	CS	1	Isonychia bicolor	Isonychiidae	Ephemeroptera	2	L/R
9/30/2011	1	CS	1	Maccaffertium mediopunctatum	Heptageniidae	Ephemeroptera	3	L/R
9/30/2011	1	CS	1	Neoperla harpi	Perlidae	Plecoptera	5	L/R
9/30/2011	1	CS	1	Neoperla osage	Perlidae	Plecoptera	6	L/R
9/30/2011	1	CS	1	Corydalis sp.	Corydalidae	Megaloptera	4	L/R
9/30/2011	1	CS	1	Corydalis sp.	Corydalidae	Megaloptera	12	
9/30/2011	1	CS	1	Heptageniidae	Heptageniidae	Ephemeroptera	7	
9/30/2011	1	CS	1	Cheumatopsyche sp.	Hydropsychidae	Tricoptera	26	
9/30/2011	1	CS	1	Tanytarsus sp.	Chironominae	Diptera	3	
9/30/2011	1	CS	2	Tanytarsus sp.	Chironominae	Diptera	1	
9/30/2011	1	CS	2	Chironomidae Pupae	Chironomidae	Diptera	2	
9/30/2011	1	CS	2	Thienemanniella sp.	Orthocladiinae	Diptera	1	
9/30/2011	1	CS	2	Thienemannimyia grp	Tanypodinae	Diptera	1	
9/30/2011	1	CS	2	Paratanytarsus sp.	Chironominae	Diptera	1	
9/30/2011	1	CS	2	Rheotanytarsus sp.	Chironominae	Diptera	1	
9/30/2011	1	CS	2	Isonychia bicolor	Isonychiidae	Ephemeroptera	9	
9/30/2011	1	CS	2	Stenonema femoratum	Heptageniidae	Ephemeroptera	1	L/R
9/30/2011	1	CS	2	Acarina sp.	Hydracarina	Arachnoidea	12	
9/30/2011	1	CS	2	Neoperla sp.	Perlidae	Plecoptera	39	
9/30/2011	1	CS	2	Acerpenna sp.	Baetidae	Ephemeroptera	10	
9/30/2011	1	CS	2	Acentrella sp.	Baetidae	Ephemeroptera	26	
9/30/2011	1	CS	2	Corydalis sp.	Corydalidae	Megaloptera	8	L/R
9/30/2011	1	CS	2	Psephenus herricki	Psephenidae	Coleoptera	2	L/R
9/30/2011	1	CS	2	Stenelmis lateralis	Elmidae	Coleoptera	3	L/R
9/30/2011	1	CS	2	Chironomidae	Chironomidae	Diptera	9	

S1 Appendix. Raw benthic macroinvertebrate data from Crane Pond Creek, Iron County, Missouri, USA.

9/30/2011	1	CS	2	Hexatoma sp.	Tipulidae	Diptera	1	L/R
9/30/2011	1	CS	2	Corydalis sp.	Corydalidae	Megaloptera	15	
9/30/2011	1	CS	2	Stenelmis sp.	Elmidae	Coleoptera	1	L/R
9/30/2011	1	CS	2	Neoperla osage	Perlidae	Plecoptera	4	L/R
9/30/2011	1	CS	2	Agnetina flavescens	Perlidae	Plecoptera	5	L/R
9/30/2011	1	CS	2	Optioservus sandersoni	Elmidae	Coleoptera	9	
9/30/2011	1	CS	2	Tabanus sp.	Tabanidae	Diptera	1	L/R
9/30/2011	1	CS	2	Acerpenna sp.	Baetidae	Ephemeroptera	4	L/R
9/30/2011	1	CS	2	Orconectes hylas	Cambaridae	Decapoda	4	L/R
9/30/2011	1	CS	2	Isonychia bicolor	Isonychiidae	Ephemeroptera	1	L/R
9/30/2011	1	CS	2	Cheumatopsyche sp.	Hydropsychidae	Tricoptera	17	
9/30/2011	1	CS	2	Orconectes hylas	Cambaridae	Decapoda	2	
9/30/2011	1	CS	2	Simulium sp.	Simuliidae	Diptera	3	
9/30/2011	1	CS	2	Psephenus herricki	Psephenidae	Coleoptera	83	
9/30/2011	1	CS	2	Bezzia sp.	Ceratopogonidae	Diptera	1	
9/30/2011	1	CS	2	Baetidae	Baetidae	Ephemeroptera	2	
9/30/2011	1	CS	2	Tabanus sp.	Tabanidae	Diptera	7	
9/30/2011	1	CS	2	Chimarra sp.	Philopotamidae	Tricoptera	2	
9/30/2011	1	CS	2	Maccaffertium mediopunctatum	Heptageniidae	Ephemeroptera	33	
9/30/2011	1	CS	2	Elimia potosensis	Pleuroceridae	Gastropoda	9	
9/30/2011	1	CS	2	Stenelmis lateralis	Elmidae	Coleoptera	145	
9/30/2011	1	CS	2	Helicopsyche sp.	Helicopsychidae	Tricoptera	2	
9/30/2011	1	CS	2	Leuctra sp.	Leuctridae	Plecoptera	3	
9/30/2011	1	CS	2	Agnetina flavescens	Perlidae	Plecoptera	26	
9/30/2011	1	CS	2	Maccaffertium sp.	Heptageniidae	Ephemeroptera	7	
9/30/2011	1	CS	2	Optioservus sp.	Elmidae	Coleoptera	13	
9/30/2011	1	CS	2	Stenonema femoratum	Heptageniidae	Ephemeroptera	5	
9/30/2011	1	CS	2	Stenelmis sp.	Elmidae	Coleoptera	131	
9/30/2011	1	CS	2	Helichus sp.	Dryopidae	Coleoptera	1	L/R
9/30/2011	1	CS	2	Caenis sp.	Caenidae	Ephemeroptera	6	
9/30/2011	1	CS	2	Cheumatopsyche sp.	Hydropsychidae	Tricoptera	3	L/R
9/30/2011	1	CS	3	Agnetina flavescens	Perlidae	Plecoptera	9	L/R
9/30/2011	1	CS	3	Polypedilum convictum	Chironominae	Diptera	1	
9/30/2011	1	CS	3	Acarina sp.	Hydracarina	Arachnoidea	9	
9/30/2011	1	CS	3	Chironomidae	Chironomidae	Diptera	23	
9/30/2011	1	CS	3	Stenochironomus sp.	Chironominae	Diptera	1	
9/30/2011	1	CS	3	Orconectes hylas	Cambaridae	Decapoda	12	L/R

S1 Appendix. Raw benthic macroinvertebrate data from Crane Pond Creek, Iron County, Missouri, USA.

9/30/2011	1	CS	3	Petrophilia sp.	Pyralidae	Lepidoptera	1	L/R
9/30/2011	1	CS	3	Isonychia bicolor	Isonychiidae	Ephemeroptera	2	L/R
9/30/2011	1	CS	3	Stylogomphus albistylus	Gomphidae	Odonata	1	L/R
9/30/2011	1	CS	3	Chironomidae Pupae	Chironomidae	Diptera	2	
9/30/2011	1	CS	3	Agnetina flavescens	Perlidae	Plecoptera	18	
9/30/2011	1	CS	3	Maccaffertium mediopunctatum	Heptageniidae	Ephemeroptera	2	L/R
9/30/2011	1	CS	3	Stenelmis sp.	Elmidae	Coleoptera	225	
9/30/2011	1	CS	3	Neoperla sp.	Perlidae	Plecoptera	4	L/R
9/30/2011	1	CS	3	Argia sp.	Coengrionidae	Odonata	4	L/R
9/30/2011	1	CS	3	Tanytarsus sp.	Chironominae	Diptera	1	
9/30/2011	1	CS	3	Thienemanniella sp.	Orthocladiinae	Diptera	1	
9/30/2011	1	CS	3	Psephenus herricki	Psephenidae	Coleoptera	2	L/R
9/30/2011	1	CS	3	Corydalis sp.	Corydalidae	Megaloptera	5	L/R
9/30/2011	1	CS	3	Maccaffertium mediopunctatum	Heptageniidae	Ephemeroptera	23	
9/30/2011	1	CS	3	Ectopria nervosa	Psephenidae	Coleoptera	1	
9/30/2011	1	CS	3	Optioservus sandersoni	Elmidae	Coleoptera	13	
9/30/2011	1	CS	3	Tricorythodes sp.	Leptohyphidae	Ephemeroptera	9	
9/30/2011	1	CS	3	Caenis sp.	Caenidae	Ephemeroptera	7	
9/30/2011	1	CS	3	Tabanus sp.	Tabanidae	Diptera	3	L/R
9/30/2011	1	CS	3	Petrophilia sp.	Pyralidae	Lepidoptera	2	
9/30/2011	1	CS	3	Psephenus herricki	Psephenidae	Coleoptera	24	
9/30/2011	1	CS	3	Helicopsyche sp.	Helicopsychidae	Tricoptera	15	
9/30/2011	1	CS	3	Nanocladius sp.	Orthocladiinae	Diptera	1	
9/30/2011	1	CS	3	Orconectes hylas	Cambaridae	Decapoda	1	
9/30/2011	1	CS	3	Physella sp.	Physidae	Gastropoda	2	
9/30/2011	1	CS	3	Oecetis sp.	Leptoceridae	Tricoptera	2	
9/30/2011	1	CS	3	Elimia potosensis	Pleuroceridae	Gastropoda	102	
9/30/2011	1	CS	3	Simulium sp.	Simuliidae	Diptera	3	
9/30/2011	1	CS	3	Optioservus sp.	Elmidae	Coleoptera	34	
9/30/2011	1	CS	3	Isonychia bicolor	Isonychiidae	Ephemeroptera	3	
9/30/2011	1	CS	3	Cheumatopsyche sp.	Hydropsychidae	Tricoptera	19	
9/30/2011	1	CS	3	Argia sp.	Coengrionidae	Odonata	3	
9/30/2011	1	CS	3	Stenelmis lateralis	Elmidae	Coleoptera	81	
9/30/2011	1	CS	3	Cricotopus bicinctus	Orthocladiinae	Diptera	5	
9/30/2011	1	CS	3	Neoperla sp.	Perlidae	Plecoptera	17	
9/30/2011	1	CS	3	Corydalis sp.	Corydalidae	Megaloptera	3	
9/30/2011	1	CS	3	Stylogomphus albistylus	Gomphidae	Odonata	1	

S1 Appendix. Raw benthic macroinvertebrate data from Crane Pond Creek, Iron County, Missouri, USA.

9/30/2011	1	CS	3	Stenonema femoratum	Heptageniidae	Ephemeroptera	4	
9/30/2011	1	CS	3	Tabanus sp.	Tabanidae	Diptera	2	
9/30/2011	1	CS	3	Chimarra sp.	Philopotamidae	Tricoptera	2	
9/30/2011	1	NF	1	Ablabesmyia sp.	Tanypodinae	Diptera	1	
9/30/2011	1	NF	1	Cladotanytarsus sp.	Chironominae	Diptera	2	
9/30/2011	1	NF	1	Stenochironomus sp.	Chironominae	Diptera	1	
9/30/2011	1	NF	1	Thienemannimyia grp	Tanypodinae	Diptera	2	
9/30/2011	1	NF	1	Tanytarsus sp.	Chironominae	Diptera	1	
9/30/2011	1	NF	1	Paratanytarsus sp.	Chironominae	Diptera	5	
9/30/2011	1	NF	1	Lumbriculidae	Lumbriculidae	Lumbriculida	4	
9/30/2011	1	NF	1	Psephenus herricki	Psephenidae	Coleoptera	3	L/R
9/30/2011	1	NF	1	Parakiefferiella sp.	Orthocladiinae	Diptera	2	
9/30/2011	1	NF	1	Thienemanniella sp.	Orthocladiinae	Diptera	1	
9/30/2011	1	NF	1	Dubiraphia sp.	Elmidae	Coleoptera	3	
9/30/2011	1	NF	1	Caenis sp.	Caenidae	Ephemeroptera	2	
9/30/2011	1	NF	1	Centroptilum sp.	Baetidae	Ephemeroptera	5	
9/30/2011	1	NF	1	Choroterpes sp.	Leptophlebiidae	Ephemeroptera	5	
9/30/2011	1	NF	1	Polycentropus sp.	Polycentropodidae	Tricoptera	2	
9/30/2011	1	NF	1	Gammarus sp.	Gammaridae	Amphipoda	3	
9/30/2011	1	NF	1	Stylogomphus albistylus	Gomphidae	Odonata	2	
9/30/2011	1	NF	1	Bezzia sp.	Ceratopogonidae	Diptera	2	
9/30/2011	1	NF	1	Tricorythodes sp.	Leptohyphidae	Ephemeroptera	2	
9/30/2011	1	NF	1	Leuctra sp.	Leuctridae	Plecoptera	1	L/R
9/30/2011	1	NF	1	Stenonema femoratum	Heptageniidae	Ephemeroptera	8	L/R
9/30/2011	1	NF	1	Argia sp.	Coengrionidae	Odonata	5	L/R
9/30/2011	1	NF	1	Neoperla sp.	Perlidae	Plecoptera	4	L/R
9/30/2011	1	NF	1	Stylogomphus albistylus	Gomphidae	Odonata	1	L/R
9/30/2011	1	NF	1	Chironomidae	Chironomidae	Diptera	19	
9/30/2011	1	NF	1	Tabanus sp.	Tabanidae	Diptera	2	L/R
9/30/2011	1	NF	1	Baetisca sp.	Baetiscidae	Ephemeroptera	39	
9/30/2011	1	NF	1	Stenonema femoratum	Heptageniidae	Ephemeroptera	22	
9/30/2011	1	NF	1	Optioservus sp.	Elmidae	Coleoptera	2	
9/30/2011	1	NF	1	Psephenus herricki	Psephenidae	Coleoptera	6	
9/30/2011	1	NF	1	Hagenius brevistylus	Gomphidae	Odonata	1	L/R
9/30/2011	1	NF	1	Acarina sp.	Hydracarina	Arachnoidea	6	
9/30/2011	1	NF	1	Stenelmis sp.	Elmidae	Coleoptera	177	
9/30/2011	1	NF	1	Glossiphoniidae	Rhynchobdellida	Hirudinea	1	

S1 Appendix. Raw benthic macroinvertebrate data from Crane Pond Creek, Iron County, Missouri, USA.

9/30/2011	1	NF	1	Ectopria nervosa	Psephenidae	Coleoptera	2	
9/30/2011	1	NF	1	Stenelmis lateralis	Elmidae	Coleoptera	10	
9/30/2011	1	NF	1	Trianodes sp.	Leptoceridae	Tricoptera	2	
9/30/2011	1	NF	1	Neoperla sp.	Perlidae	Plecoptera	28	
9/30/2011	1	NF	1	Helicopsyche sp.	Helicopsychidae	Tricoptera	1	
9/30/2011	1	NF	1	Elimia potosensis	Pleuroceridae	Gastropoda	2	
9/30/2011	1	NF	1	Argia sp.	Coengrionidae	Odonata	1	
9/30/2011	1	NF	1	Oecetis sp.	Leptoceridae	Tricoptera	8	
9/30/2011	1	NF	2	Neoperla sp.	Perlidae	Plecoptera	3	
9/30/2011	1	NF	2	Leuctra sp.	Leuctridae	Plecoptera	1	
9/30/2011	1	NF	2	Optioservus sp.	Elmidae	Coleoptera	7	
9/30/2011	1	NF	2	Choroterpes sp.	Leptophlebiidae	Ephemeroptera	1	
9/30/2011	1	NF	2	Stylogomphus albistylus	Gomphidae	Odonata	9	
9/30/2011	1	NF	2	Oecetis sp.	Leptoceridae	Tricoptera	2	
9/30/2011	1	NF	2	Psephenus herricki	Psephenidae	Coleoptera	2	L/R
9/30/2011	1	NF	2	Ectopria nervosa	Psephenidae	Coleoptera	2	L/R
9/30/2011	1	NF	2	Stenelmis sp.	Elmidae	Coleoptera	2	L/R
9/30/2011	1	NF	2	Dubiraphia sp.	Elmidae	Coleoptera	4	
9/30/2011	1	NF	2	Tricorythodes sp.	Leptohyphidae	Ephemeroptera	2	L/R
9/30/2011	1	NF	2	Acarina sp.	Hydracarina	Arachnoidea	8	
9/30/2011	1	NF	2	Choroterpes sp.	Leptophlebiidae	Ephemeroptera	1	L/R
9/30/2011	1	NF	2	Chironomidae	Chironomidae	Diptera	4	
9/30/2011	1	NF	2	Stenonema femoratum	Heptageniidae	Ephemeroptera	8	L/R
9/30/2011	1	NF	2	Elimia potosensis	Pleuroceridae	Gastropoda	5	
9/30/2011	1	NF	2	Corynoneura sp.	Orthocladiinae	Diptera	1	
9/30/2011	1	NF	2	Neoperla sp.	Perlidae	Plecoptera	3	L/R
9/30/2011	1	NF	2	Tanytarsus sp.	Chironominae	Diptera	1	
9/30/2011	1	NF	2	Lumbriculidae	Lumbriculidae	Lumbriculida	2	
9/30/2011	1	NF	2	Stenonema femoratum	Heptageniidae	Ephemeroptera	18	
9/30/2011	1	NF	2	Serratella sp.	Ephemerellidae	Ephemeroptera	3	
9/30/2011	1	NF	2	Psephenus herricki	Psephenidae	Coleoptera	7	
9/30/2011	1	NF	2	Helicopsyche sp.	Helicopsychidae	Tricoptera	1	
9/30/2011	1	NF	2	Stenelmis sp.	Elmidae	Coleoptera	184	
9/30/2011	1	NF	2	Centroptilum sp.	Baetidae	Ephemeroptera	1	
9/30/2011	1	NF	2	Trianodes sp.	Leptoceridae	Tricoptera	2	
9/30/2011	1	NF	2	Caenis sp.	Caenidae	Ephemeroptera	1	
9/30/2011	1	NF	2	Baetisca sp.	Baetiscidae	Ephemeroptera	37	

S1 Appendix. Raw benthic macroinvertebrate data from Crane Pond Creek, Iron County, Missouri, USA.

9/30/2011	1	NF	2	Bezzia sp.	Ceratopogonidae	Diptera	3	
9/30/2011	1	NF	2	Ectopria nervosa	Psephenidae	Coleoptera	2	
9/30/2011	1	NF	3	Didymops sp.	Corduliidae	Odonata	1	L/R
9/30/2011	1	NF	3	Ectopria nervosa	Psephenidae	Coleoptera	2	
9/30/2011	1	NF	3	Optioservus sp.	Elmidae	Coleoptera	1	
9/30/2011	1	NF	3	Thienemannimyia grp	Tanypodinae	Diptera	1	
9/30/2011	1	NF	3	Ectopria nervosa	Psephenidae	Coleoptera	1	L/R
9/30/2011	1	NF	3	Stenonema femoratum	Heptageniidae	Ephemeroptera	5	L/R
9/30/2011	1	NF	3	Neoperla sp.	Perlidae	Plecoptera	7	L/R
9/30/2011	1	NF	3	Ablabesmyia sp.	Tanypodinae	Diptera	1	
9/30/2011	1	NF	3	Stylogomphus albistylus	Gomphidae	Odonata	4	
9/30/2011	1	NF	3	Cladotanytarsus sp.	Chironominae	Diptera	6	
9/30/2011	1	NF	3	Argia sp.	Coengrionidae	Odonata	2	L/R
9/30/2011	1	NF	3	Chironomidae	Chironomidae	Diptera	44	
9/30/2011	1	NF	3	Cryptochironomus sp.	Chironominae	Diptera	1	
9/30/2011	1	NF	3	Paratanytarsus sp.	Chironominae	Diptera	13	
9/30/2011	1	NF	3	Dicrotendipes sp.	Chironominae	Diptera	1	
9/30/2011	1	NF	3	Lauterborniella sp.	Chironominae	Diptera	3	
9/30/2011	1	NF	3	Psephenus herricki	Psephenidae	Coleoptera	12	
9/30/2011	1	NF	3	Tanytarsus sp.	Chironominae	Diptera	3	
9/30/2011	1	NF	3	Parakiefferiella sp.	Orthocladiinae	Diptera	1	
9/30/2011	1	NF	3	Tricorythodes sp.	Leptohyphidae	Ephemeroptera	1	
9/30/2011	1	NF	3	Stenelmis sp.	Elmidae	Coleoptera	146	
9/30/2011	1	NF	3	Argia sp.	Coengrionidae	Odonata	1	
9/30/2011	1	NF	3	Hagenius brevistylus	Gomphidae	Odonata	2	L/R
9/30/2011	1	NF	3	Leuctra sp.	Leuctridae	Plecoptera	5	
9/30/2011	1	NF	3	Polycentropus sp.	Polycentropodidae	Tricoptera	1	
9/30/2011	1	NF	3	Stenelmis lateralis	Elmidae	Coleoptera	20	
9/30/2011	1	NF	3	Elimia potosensis	Pleuroceridae	Gastropoda	1	
9/30/2011	1	NF	3	Psephenus herricki	Psephenidae	Coleoptera	1	L/R
9/30/2011	1	NF	3	Choroterpes sp.	Leptophlebiidae	Ephemeroptera	4	
9/30/2011	1	NF	3	Neoperla sp.	Perlidae	Plecoptera	20	
9/30/2011	1	NF	3	Stenonema femoratum	Heptageniidae	Ephemeroptera	33	
9/30/2011	1	NF	3	Chironomidae Pupae	Chironomidae	Diptera	3	
9/30/2011	1	NF	3	Baetisca sp.	Baetiscidae	Ephemeroptera	3	
9/30/2011	1	NF	3	Caenis sp.	Caenidae	Ephemeroptera	8	
9/30/2011	1	NF	3	Acarina sp.	Hydracarina	Arachnoidea	5	

S1 Appendix. Raw benthic macroinvertebrate data from Crane Pond Creek, Iron County, Missouri, USA.

9/30/2011	1	NF	3	Oecetis sp.	Leptoceridae	Tricoptera	7	
9/30/2011	1	NF	3	Centroptilum sp.	Baetidae	Ephemeroptera	1	
9/30/2011	1	NF	3	Hagenius brevistylus	Gomphidae	Odonata	1	
9/30/2011	2	CS	1	Optioservus sandersoni	Elmidae	Coleoptera	2	
9/30/2011	2	CS	1	Tabanus sp.	Tabanidae	Diptera	1	
9/30/2011	2	CS	1	Dicrotendipes sp.	Chironominae	Diptera	1	
9/30/2011	2	CS	1	Maccaffertium mediopunctatum	Heptageniidae	Ephemeroptera	11	
9/30/2011	2	CS	1	Stenonema femoratum	Heptageniidae	Ephemeroptera	1	
9/30/2011	2	CS	1	Cheumatopsyche sp.	Hydropsychidae	Tricoptera	15	
9/30/2011	2	CS	1	Helicopsyche sp.	Helicopsychidae	Tricoptera	39	
9/30/2011	2	CS	1	Stylogomphus albistylus	Gomphidae	Odonata	33	
9/30/2011	2	CS	1	Tricorythodes sp.	Leptohyphidae	Ephemeroptera	5	
9/30/2011	2	CS	1	Stenelmis lateralis	Elmidae	Coleoptera	40	
9/30/2011	2	CS	1	Ancylidae	Ancylidae	Limnophila	2	
9/30/2011	2	CS	1	Oecetis sp.	Leptoceridae	Tricoptera	2	
9/30/2011	2	CS	1	Acarina sp.	Hydracarina	Arachnoidea	10	
9/30/2011	2	CS	1	Agnetina flavescens	Perlidae	Plecoptera	3	
9/30/2011	2	CS	1	Baetisca sp.	Baetiscidae	Ephemeroptera	1	
9/30/2011	2	CS	1	Cricotopus bicinctus	Orthocladiinae	Diptera	7	
9/30/2011	2	CS	1	Isonychia bicolor	Isonychiidae	Ephemeroptera	5	
9/30/2011	2	CS	1	Caenis sp.	Caenidae	Ephemeroptera	6	
9/30/2011	2	CS	1	Chironomidae Pupae	Chironomidae	Diptera	7	
9/30/2011	2	CS	1	Ablabesmyia sp.	Tanypodinae	Diptera	2	
9/30/2011	2	CS	1	Nigronia sp.	Corydalidae	Megaloptera	2	
9/30/2011	2	CS	1	Neoperla sp.	Perlidae	Plecoptera	3	L/R
9/30/2011	2	CS	1	Ectopria nervosa	Psephenidae	Coleoptera	1	L/R
9/30/2011	2	CS	1	Physella sp.	Physidae	Gastropoda	2	
9/30/2011	2	CS	1	Psephenus herricki	Psephenidae	Coleoptera	1	L/R
9/30/2011	2	CS	1	Psephenus herricki	Psephenidae	Coleoptera	61	
9/30/2011	2	CS	1	Maccaffertium mediopunctatum	Heptageniidae	Ephemeroptera	1	L/R
9/30/2011	2	CS	1	Isonychia bicolor	Isonychiidae	Ephemeroptera	3	L/R
9/30/2011	2	CS	1	Paratanytarsus sp.	Chironominae	Diptera	2	
9/30/2011	2	CS	1	Argia sp.	Coengrionidae	Odonata	7	L/R
9/30/2011	2	CS	1	Tabanus sp.	Tabanidae	Diptera	2	L/R
9/30/2011	2	CS	1	Ancylidae	Ancylidae	Limnophila	1	L/R
9/30/2011	2	CS	1	Rheotanytarsus sp.	Chironominae	Diptera	1	
9/30/2011	2	CS	1	Orconectes peruncus	Cambaridae	Decapoda	2	L/R

S1 Appendix. Raw benthic macroinvertebrate data from Crane Pond Creek, Iron County, Missouri, USA.

9/30/2011	2	CS	1	Orconectes luteus	Cambaridae	Decapoda	1	L/R
9/30/2011	2	CS	1	Chironomidae	Chironomidae	Diptera	9	
9/30/2011	2	CS	1	Tanytarsus sp.	Chironominae	Diptera	2	
9/30/2011	2	CS	1	Thienemanniella sp.	Orthocladiinae	Diptera	3	
9/30/2011	2	CS	1	Stenelmis sp.	Elmidae	Coleoptera	2	L/R
9/30/2011	2	CS	1	Maccaffertium sp.	Heptageniidae	Ephemeroptera	37	
9/30/2011	2	CS	1	Neoperla sp.	Perlidae	Plecoptera	22	
9/30/2011	2	CS	1	Polycentropus sp.	Polycentropodidae	Tricoptera	3	
9/30/2011	2	CS	1	Optioservus sp.	Elmidae	Coleoptera	13	
9/30/2011	2	CS	1	Centroptilum sp.	Baetidae	Ephemeroptera	1	
9/30/2011	2	CS	1	Corydalis sp.	Corydalidae	Megaloptera	2	L/R
9/30/2011	2	CS	1	Stenelmis sp.	Elmidae	Coleoptera	199	
9/30/2011	2	CS	1	Lirceus sp.	Asellidae	Isopoda	2	
9/30/2011	2	CS	1	Heptageniidae	Heptageniidae	Ephemeroptera	9	
9/30/2011	2	CS	1	Choroterpes sp.	Leptophlebiidae	Ephemeroptera	2	
9/30/2011	2	CS	1	Corydalis sp.	Corydalidae	Megaloptera	3	
9/30/2011	2	CS	1	Nigronia sp.	Corydalidae	Megaloptera	1	L/R
9/30/2011	2	CS	1	Petrophilia sp.	Pyralidae	Lepidoptera	1	
9/30/2011	2	CS	1	Argia sp.	Coengrionidae	Odonata	38	
9/30/2011	2	CS	2	Chironomidae Pupae	Chironomidae	Diptera	4	
9/30/2011	2	CS	2	Thienemanniella sp.	Orthocladiinae	Diptera	5	
9/30/2011	2	CS	2	Orthocladius sp. (yellow dome tooth)	Orthocladiinae	Diptera	1	
9/30/2011	2	CS	2	Nilotanypus sp.	Tanypodinae	Diptera	2	
9/30/2011	2	CS	2	Optioservus sandersoni	Elmidae	Coleoptera	1	
9/30/2011	2	CS	2	Polypedilum convictum	Chironominae	Diptera	5	
9/30/2011	2	CS	2	Rheocricotopus sp.	Orthocladiinae	Diptera	9	
9/30/2011	2	CS	2	Chaetocladius sp.	Orthocladiinae	Diptera	2	
9/30/2011	2	CS	2	Phaenopsectra sp.	Chironominae	Diptera	2	
9/30/2011	2	CS	2	Eukiefferiella sp.	Orthocladiinae	Diptera	1	
9/30/2011	2	CS	2	Cricotopus bicinctus	Orthocladiinae	Diptera	5	
9/30/2011	2	CS	2	Polypedilum (sp. A)	Chironominae	Diptera	1	
9/30/2011	2	CS	2	Rheotanytarsus sp.	Chironominae	Diptera	6	
9/30/2011	2	CS	2	Stylogomphus albistylus	Gomphidae	Odonata	2	L/R
9/30/2011	2	CS	2	Glossiphoniidae	Rhynchobdellida	Hirudinea	1	
9/30/2011	2	CS	2	Nigronia sp.	Corydalidae	Megaloptera	1	
9/30/2011	2	CS	2	Polycentropus sp.	Polycentropodidae	Tricoptera	5	
9/30/2011	2	CS	2	Physella sp.	Physidae	Gastropoda	1	

S1 Appendix. Raw benthic macroinvertebrate data from Crane Pond Creek, Iron County, Missouri, USA.

9/30/2011	2	CS	2	Maccaffertium sp.	Heptageniidae	Ephemeroptera	24	
9/30/2011	2	CS	2	Chimarra sp.	Philopotamidae	Tricoptera	1	
9/30/2011	2	CS	2	Helicopsyche sp.	Helicopsychidae	Tricoptera	15	
9/30/2011	2	CS	2	Tipula sp.	Tipulidae	Diptera	1	
9/30/2011	2	CS	2	Choroterpes sp.	Leptophlebiidae	Ephemeroptera	7	
9/30/2011	2	CS	2	Acarina sp.	Hydracarina	Arachnoidea	22	
9/30/2011	2	CS	2	Isonychia bicolor	Isonychiidae	Ephemeroptera	4	L/R
9/30/2011	2	CS	2	Metrobates sp.	Gerridae	Hemiptera	1	L/R
9/30/2011	2	CS	2	Agnetina flavescens	Perlidae	Plecoptera	3	L/R
9/30/2011	2	CS	2	Argia sp.	Coengrionidae	Odonata	2	L/R
9/30/2011	2	CS	2	Stenelmis sp.	Elmidae	Coleoptera	1	L/R
9/30/2011	2	CS	2	Psephenus herricki	Psephenidae	Coleoptera	1	L/R
9/30/2011	2	CS	2	Ectopria nervosa	Psephenidae	Coleoptera	1	L/R
9/30/2011	2	CS	2	Nigronia sp.	Corydalidae	Megaloptera	1	L/R
9/30/2011	2	CS	2	Orconectes peruncus	Cambaridae	Decapoda	1	L/R
9/30/2011	2	CS	2	Orconectes sp.	Cambaridae	Decapoda	1	L/R
9/30/2011	2	CS	2	Chironomidae	Chironomidae	Diptera	9	
9/30/2011	2	CS	2	Simulium sp.	Simuliidae	Diptera	7	
9/30/2011	2	CS	2	Corydalis sp.	Corydalidae	Megaloptera	6	L/R
9/30/2011	2	CS	2	Neoperla sp.	Perlidae	Plecoptera	17	
9/30/2011	2	CS	2	Centroptilum sp.	Baetidae	Ephemeroptera	7	
9/30/2011	2	CS	2	Stenelmis lateralis	Elmidae	Coleoptera	35	
9/30/2011	2	CS	2	Tricorythodes sp.	Leptohyphidae	Ephemeroptera	7	
9/30/2011	2	CS	2	Oecetis sp.	Leptoceridae	Tricoptera	2	
9/30/2011	2	CS	2	Maccaffertium mediopunctatum	Heptageniidae	Ephemeroptera	6	
9/30/2011	2	CS	2	Stylogomphus albistylus	Gomphidae	Odonata	41	
9/30/2011	2	CS	2	Cheumatopsyche sp.	Hydropsychidae	Tricoptera	20	
9/30/2011	2	CS	2	Petrophilia sp.	Pyralidae	Lepidoptera	2	
9/30/2011	2	CS	2	Argia sp.	Coengrionidae	Odonata	17	
9/30/2011	2	CS	2	Isonychia bicolor	Isonychiidae	Ephemeroptera	23	
9/30/2011	2	CS	2	Stenelmis sp.	Elmidae	Coleoptera	188	
9/30/2011	2	CS	2	Hydropsyche sp.	Hydropsychidae	Tricoptera	1	
9/30/2011	2	CS	2	Lirceus sp.	Asellidae	Isopoda	4	
9/30/2011	2	CS	2	Stenonema femoratum	Heptageniidae	Ephemeroptera	4	
9/30/2011	2	CS	2	Optioservus sp.	Elmidae	Coleoptera	9	
9/30/2011	2	CS	2	Corydalis sp.	Corydalidae	Megaloptera	5	
9/30/2011	2	CS	2	Caenis sp.	Caenidae	Ephemeroptera	2	

S1 Appendix. Raw benthic macroinvertebrate data from Crane Pond Creek, Iron County, Missouri, USA.

9/30/2011	2	CS	2	Hemerodromia sp.	Empididae	Diptera	2	
9/30/2011	2	CS	2	Agnetina flavescens	Perlidae	Plecoptera	10	
9/30/2011	2	CS	2	Psephenus herricki	Psephenidae	Coleoptera	58	
9/30/2011	2	CS	3	Argia sp.	Coengrionidae	Odonata	3	
9/30/2011	2	CS	3	Maccaffertium mediopunctatum	Heptageniidae	Ephemeroptera	13	
9/30/2011	2	CS	3	Stenonema femoratum	Heptageniidae	Ephemeroptera	3	
9/30/2011	2	CS	3	Optioservus sandersoni	Elmidae	Coleoptera	2	
9/30/2011	2	CS	3	Ancylidae	Ancylidae	Limnophila	3	
9/30/2011	2	CS	3	Tabanus sp.	Tabanidae	Diptera	3	L/R
9/30/2011	2	CS	3	Acentrella sp.	Baetidae	Ephemeroptera	5	
9/30/2011	2	CS	3	Isonychia bicolor	Isonychiidae	Ephemeroptera	125	
9/30/2011	2	CS	3	Simulium sp.	Simuliidae	Diptera	7	
9/30/2011	2	CS	3	Baetisca sp.	Baetiscidae	Ephemeroptera	3	
9/30/2011	2	CS	3	Maccaffertium sp.	Heptageniidae	Ephemeroptera	62	
9/30/2011	2	CS	3	Stenelmis sp.	Elmidae	Coleoptera	104	
9/30/2011	2	CS	3	Orconectes peruncus	Cambaridae	Decapoda	1	
9/30/2011	2	CS	3	Agnetina flavescens	Perlidae	Plecoptera	12	L/R
9/30/2011	2	CS	3	Psephenus herricki	Psephenidae	Coleoptera	3	L/R
9/30/2011	2	CS	3	Neoperla sp.	Perlidae	Plecoptera	2	L/R
9/30/2011	2	CS	3	Corydalis sp.	Corydalidae	Megaloptera	11	
9/30/2011	2	CS	3	Helicopsyche sp.	Helicopsychidae	Tricoptera	6	
9/30/2011	2	CS	3	Isonychia bicolor	Isonychiidae	Ephemeroptera	4	L/R
9/30/2011	2	CS	3	Stenonema femoratum	Heptageniidae	Ephemeroptera	1	L/R
9/30/2011	2	CS	3	Maccaffertium mediopunctatum	Heptageniidae	Ephemeroptera	1	L/R
9/30/2011	2	CS	3	Cheumatopsyche sp.	Hydropsychidae	Tricoptera	1	L/R
9/30/2011	2	CS	3	Orconectes peruncus	Cambaridae	Decapoda	11	L/R
9/30/2011	2	CS	3	Corydalis sp.	Corydalidae	Megaloptera	7	L/R
9/30/2011	2	CS	3	Lirceus sp.	Asellidae	Isopoda	4	
9/30/2011	2	CS	3	Rheotanytarsus sp.	Chironominae	Diptera	1	
9/30/2011	2	CS	3	Rheocricotopus sp.	Orthocladiinae	Diptera	1	
9/30/2011	2	CS	3	Thienemanniella sp.	Orthocladiinae	Diptera	1	
9/30/2011	2	CS	3	Polypedilum convictum	Chironominae	Diptera	10	
9/30/2011	2	CS	3	Thienemannimyia grp	Tanypodinae	Diptera	3	
9/30/2011	2	CS	3	Orconectes luteus	Cambaridae	Decapoda	1	
9/30/2011	2	CS	3	Chironomidae	Chironomidae	Diptera	48	
9/30/2011	2	CS	3	Optioservus sp.	Elmidae	Coleoptera	4	
9/30/2011	2	CS	3	Phaenopsectra sp.	Chironominae	Diptera	1	

S1 Appendix. Raw benthic macroinvertebrate data from Crane Pond Creek, Iron County, Missouri, USA.

9/30/2011	2	CS	3	Physella sp.	Physidae	Gastropoda	2	
9/30/2011	2	CS	3	Acarina sp.	Hydracarina	Arachnoidea	2	
9/30/2011	2	CS	3	Hydropsyche sp.	Hydropsychidae	Tricoptera	1	
9/30/2011	2	CS	3	Stenelmis lateralis	Elmidae	Coleoptera	31	
9/30/2011	2	CS	3	Polycentropus sp.	Polycentropodidae	Tricoptera	6	
9/30/2011	2	CS	3	Tabanus sp.	Tabanidae	Diptera	3	
9/30/2011	2	CS	3	Polypedilum (sp. A)	Chironominae	Diptera	5	
9/30/2011	2	CS	3	Centroptilum sp.	Baetidae	Ephemeroptera	5	
9/30/2011	2	CS	3	Cheumatopsyche sp.	Hydropsychidae	Tricoptera	100	
9/30/2011	2	CS	3	Agnetina flavescens	Perlidae	Plecoptera	7	
9/30/2011	2	CS	3	Acentrella sp.	Baetidae	Ephemeroptera	2	
9/30/2011	2	CS	3	Caenis sp.	Caenidae	Ephemeroptera	4	
9/30/2011	2	CS	3	Neoperla sp.	Perlidae	Plecoptera	16	
9/30/2011	2	CS	3	Tricorythodes sp.	Leptohyphidae	Ephemeroptera	8	
9/30/2011	2	CS	3	Chimarra sp.	Philopotamidae	Tricoptera	5	
9/30/2011	2	CS	3	Psephenus herricki	Psephenidae	Coleoptera	12	
9/30/2011	2	CS	3	Cricotopus bicinctus	Orthocladiinae	Diptera	3	
9/30/2011	2	CS	3	Chironomidae Pupae	Chironomidae	Diptera	6	
9/30/2011	2	CS	3	Chironomus sp.	Chironominae	Diptera	1	
9/30/2011	2	NF	1	Phaenopsectra sp.	Chironominae	Diptera	1	
9/30/2011	2	NF	1	Stylogomphus albistylus	Gomphidae	Odonata	9	
9/30/2011	2	NF	1	Ectopria nervosa	Psephenidae	Coleoptera	2	L/R
9/30/2011	2	NF	1	Ablabesmyia sp.	Tanypodinae	Diptera	1	
9/30/2011	2	NF	1	Bezzia sp.	Ceratopogonidae	Diptera	2	
9/30/2011	2	NF	1	Stenonema femoratum	Heptageniidae	Ephemeroptera	126	
9/30/2011	2	NF	1	Didymops sp.	Corduliidae	Odonata	2	
9/30/2011	2	NF	1	Caenis sp.	Caenidae	Ephemeroptera	19	
9/30/2011	2	NF	1	Argia sp.	Coengrionidae	Odonata	17	
9/30/2011	2	NF	1	Psephenus herricki	Psephenidae	Coleoptera	3	
9/30/2011	2	NF	1	Oecetis sp.	Leptoceridae	Tricoptera	1	
9/30/2011	2	NF	1	Parakiefferiella sp.	Orthocladiinae	Diptera	1	
9/30/2011	2	NF	1	Stenelmis sp.	Elmidae	Coleoptera	50	
9/30/2011	2	NF	1	Ectopria nervosa	Psephenidae	Coleoptera	5	
9/30/2011	2	NF	1	Paralauterborniella sp.	Chironominae	Diptera	2	
9/30/2011	2	NF	1	Thienemannimyia grp	Tanypodinae	Diptera	1	
9/30/2011	2	NF	1	Stenonema femoratum	Heptageniidae	Ephemeroptera	6	L/R
9/30/2011	2	NF	1	Paratanytarsus sp.	Chironominae	Diptera	4	

S1 Appendix. Raw benthic macroinvertebrate data from Crane Pond Creek, Iron County, Missouri, USA.

9/30/2011	2	NF	1	Chironomidae	Chironomidae	Diptera	30	
9/30/2011	2	NF	1	Thienemanniella sp.	Orthocladiinae	Diptera	2	
9/30/2011	2	NF	1	Ancylidae	Ancylidae	Limnophila	5	
9/30/2011	2	NF	1	Polypedilum (sp. A)	Chironominae	Diptera	1	
9/30/2011	2	NF	1	Stenochironomus sp.	Chironominae	Diptera	3	
9/30/2011	2	NF	1	Tanytarsus sp.	Chironominae	Diptera	1	
9/30/2011	2	NF	1	Argia sp.	Coengrionidae	Odonata	2	L/R
9/30/2011	2	NF	1	Dubiraphia sp.	Elmidae	Coleoptera	13	
9/30/2011	2	NF	1	Serratella sp.	Ephemerellidae	Ephemeroptera	7	
9/30/2011	2	NF	1	Trepobates becki	Gerridae	Hemiptera	1	
9/30/2011	2	NF	1	Corynoneura sp.	Orthocladiinae	Diptera	1	
9/30/2011	2	NF	1	Baetisca sp.	Baetiscidae	Ephemeroptera	2	
9/30/2011	2	NF	1	Gyraulus sp.	Planorbidae	Gastropoda	2	
9/30/2011	2	NF	1	Dicrotendipes sp.	Chironominae	Diptera	1	
9/30/2011	2	NF	1	Stenelmis lateralis	Elmidae	Coleoptera	3	
9/30/2011	2	NF	1	Polycentropus sp.	Polycentropodidae	Tricoptera	3	
9/30/2011	2	NF	1	Acarina sp.	Hydracarina	Arachnoidea	18	
9/30/2011	2	NF	1	Helicopsyche sp.	Helicopsychidae	Tricoptera	1	
9/30/2011	2	NF	1	Orconectes luteus	Cambaridae	Decapoda	1	
9/30/2011	2	NF	1	Diptera		Diptera	1	
9/30/2011	2	NF	1	Neoperla sp.	Perlidae	Plecoptera	2	
9/30/2011	2	NF	1	Microtendipes sp.	Chironominae	Diptera	1	
9/30/2011	2	NF	1	Orconectes luteus	Cambaridae	Decapoda	4	L/R
9/30/2011	2	NF	1	Chironomidae Pupae	Chironomidae	Diptera	7	
9/30/2011	2	NF	1	Lauterborniella sp.	Chironominae	Diptera	1	
9/30/2011	2	NF	1	Cladotanytarsus sp.	Chironominae	Diptera	1	
9/30/2011	2	NF	1	Tricorythodes sp.	Leptohyphidae	Ephemeroptera	2	
9/30/2011	2	NF	2	Ancylidae	Ancylidae	Limnophila	1	
9/30/2011	2	NF	2	Acarina sp.	Hydracarina	Arachnoidea	6	
9/30/2011	2	NF	2	Corbicula sp.	Corbiculidae	Veroida	1	
9/30/2011	2	NF	2	Psephenus herricki	Psephenidae	Coleoptera	4	
9/30/2011	2	NF	2	Tabanus sp.	Tabanidae	Diptera	2	
9/30/2011	2	NF	2	Polypedilum convictum	Chironominae	Diptera	1	
9/30/2011	2	NF	2	Serratella sp.	Ephemerellidae	Ephemeroptera	4	
9/30/2011	2	NF	2	Stenelmis lateralis	Elmidae	Coleoptera	1	
9/30/2011	2	NF	2	Dubiraphia sp.	Elmidae	Coleoptera	31	
9/30/2011	2	NF	2	Bezzia sp.	Ceratopogonidae	Diptera	2	

S1 Appendix. Raw benthic macroinvertebrate data from Crane Pond Creek, Iron County, Missouri, USA.

9/30/2011	2	NF	2	Orconectes luteus	Cambaridae	Decapoda	1	
9/30/2011	2	NF	2	Argia sp.	Coengrionidae	Odonata	8	
9/30/2011	2	NF	2	Procladius sp.	Tanypodinae	Diptera	4	
9/30/2011	2	NF	2	Psephenus herricki	Psephenidae	Coleoptera	1	L/R
9/30/2011	2	NF	2	Centroptilum sp.	Baetidae	Ephemeroptera	1	L/R
9/30/2011	2	NF	2	Stenochironomus sp.	Chironominae	Diptera	2	
9/30/2011	2	NF	2	Didymops sp.	Corduliidae	Odonata	1	
9/30/2011	2	NF	2	Baetisca sp.	Baetiscidae	Ephemeroptera	1	
9/30/2011	2	NF	2	Nigronia sp.	Corydalidae	Megaloptera	1	
9/30/2011	2	NF	2	Chironomidae	Chironomidae	Diptera	82	
9/30/2011	2	NF	2	Trepobates becki	Gerridae	Hemiptera	1	
9/30/2011	2	NF	2	Tanytarsus sp.	Chironominae	Diptera	7	
9/30/2011	2	NF	2	Stenonema femoratum	Heptageniidae	Ephemeroptera	7	L/R
9/30/2011	2	NF	2	Choroterpes sp.	Leptophlebiidae	Ephemeroptera	2	
9/30/2011	2	NF	2	Paralauterborniella sp.	Chironominae	Diptera	1	
9/30/2011	2	NF	2	Ablabesmyia sp.	Tanypodinae	Diptera	14	
9/30/2011	2	NF	2	Nanocladius sp.	Orthocladiinae	Diptera	1	
9/30/2011	2	NF	2	Microtendipes sp.	Chironominae	Diptera	4	
9/30/2011	2	NF	2	Orthocladius sp. (yellow dome tooth)	Orthocladiinae	Diptera	1	
9/30/2011	2	NF	2	Paracladopelma sp.	Chironominae	Diptera	1	
9/30/2011	2	NF	2	Parakiefferiella sp.	Orthocladiinae	Diptera	11	
9/30/2011	2	NF	2	Cladotanytarsus sp.	Chironominae	Diptera	7	
9/30/2011	2	NF	2	Tubificidae	Tubificidae	Tubificida	2	
9/30/2011	2	NF	2	Lumbriculidae	Lumbriculidae	Lumbriculida	1	
9/30/2011	2	NF	2	Thienemannimyia grp	Tanypodinae	Diptera	1	
9/30/2011	2	NF	2	Polycentropus sp.	Polycentropodidae	Tricoptera	1	
9/30/2011	2	NF	2	Dicrotendipes sp.	Chironominae	Diptera	1	
9/30/2011	2	NF	2	Stylogomphus albistylus	Gomphidae	Odonata	9	
9/30/2011	2	NF	2	Chironomidae Pupae	Chironomidae	Diptera	4	
9/30/2011	2	NF	2	Corynoneura sp.	Orthocladiinae	Diptera	1	
9/30/2011	2	NF	2	Stenelmis sp.	Elmidae	Coleoptera	15	
9/30/2011	2	NF	2	Paratanytarsus sp.	Chironominae	Diptera	1	
9/30/2011	2	NF	2	Caenis sp.	Caenidae	Ephemeroptera	59	
9/30/2011	2	NF	2	Gyraulus sp.	Planorbidae	Gastropoda	1	
9/30/2011	2	NF	2	Stenonema femoratum	Heptageniidae	Ephemeroptera	67	
9/30/2011	2	NF	2	Lauterborniella sp.	Chironominae	Diptera	1	
9/30/2011	2	NF	2	Centroptilum sp.	Baetidae	Ephemeroptera	4	

S1 Appendix. Raw benthic macroinvertebrate data from Crane Pond Creek, Iron County, Missouri, USA.

9/30/2011	2	NF	2	Phaenopsectra sp.	Chironominae	Diptera	3	
9/30/2011	2	NF	2	Optioservus sp.	Elmidae	Coleoptera	1	
9/30/2011	2	NF	2	Cryptochironomus sp.	Chironominae	Diptera	3	
9/30/2011	2	NF	2	Ectopria nervosa	Psephenidae	Coleoptera	11	
9/30/2011	2	NF	3	Thienemannimyia grp	Tanypodinae	Diptera	1	
9/30/2011	2	NF	3	Chironomidae Pupae	Chironomidae	Diptera	1	
9/30/2011	2	NF	3	Stenonema femoratum	Heptageniidae	Ephemeroptera	29	
9/30/2011	2	NF	3	Psephenus herricki	Psephenidae	Coleoptera	1	L/R
9/30/2011	2	NF	3	Stenelmis sp.	Elmidae	Coleoptera	1	L/R
9/30/2011	2	NF	3	Paratanytarsus sp.	Chironominae	Diptera	2	
9/30/2011	2	NF	3	Stenonema femoratum	Heptageniidae	Ephemeroptera	10	L/R
9/30/2011	2	NF	3	Phaenopsectra sp.	Chironominae	Diptera	1	
9/30/2011	2	NF	3	Chironomidae	Chironomidae	Diptera	78	
9/30/2011	2	NF	3	Stenelmis sp.	Elmidae	Coleoptera	8	
9/30/2011	2	NF	3	Parakiefferiella sp.	Orthocladiinae	Diptera	4	
9/30/2011	2	NF	3	Ablabesmyia sp.	Tanypodinae	Diptera	2	
9/30/2011	2	NF	3	Lauterborniella sp.	Chironominae	Diptera	1	
9/30/2011	2	NF	3	Chironomus sp.	Chironominae	Diptera	1	
9/30/2011	2	NF	3	Cladotanytarsus sp.	Chironominae	Diptera	33	
9/30/2011	2	NF	3	Cryptochironomus sp.	Chironominae	Diptera	2	
9/30/2011	2	NF	3	Choroterpes sp.	Leptophlebiidae	Ephemeroptera	2	L/R
9/30/2011	2	NF	3	Centroptilum sp.	Baetidae	Ephemeroptera	5	
9/30/2011	2	NF	3	Glossiphoniidae	Rhynchobdellida	Hirudinea	1	
9/30/2011	2	NF	3	Dubiraphia sp.	Elmidae	Coleoptera	24	
9/30/2011	2	NF	3	Choroterpes sp.	Leptophlebiidae	Ephemeroptera	6	
9/30/2011	2	NF	3	Ectopria nervosa	Psephenidae	Coleoptera	2	
9/30/2011	2	NF	3	Didymops sp.	Corduliidae	Odonata	1	
9/30/2011	2	NF	3	Gomphidae	Gomphidae	Odonata	1	
9/30/2011	2	NF	3	Serratella sp.	Ephemerellidae	Ephemeroptera	2	
9/30/2011	2	NF	3	Tanytarsus sp.	Chironominae	Diptera	12	
9/30/2011	2	NF	3	Acarina sp.	Hydracarina	Arachnoidea	7	
9/30/2011	2	NF	3	Bezzia sp.	Ceratopogonidae	Diptera	1	
9/30/2011	2	NF	3	Tabanus sp.	Tabanidae	Diptera	1	
9/30/2011	2	NF	3	Caenis sp.	Caenidae	Ephemeroptera	142	
9/30/2011	2	NF	3	Baetisca sp.	Baetiscidae	Ephemeroptera	3	
9/30/2011	2	NF	3	Stylogomphus albistylus	Gomphidae	Odonata	1	
9/30/2011	2	NF	3	Hexagenia sp.	Ephemeridae	Ephemeroptera	2	
